# Influences of the community and consumer nutrition environment on the food purchases and dietary behaviors of adolescents: A systematic review

**DOI:** 10.1111/obr.13569

**Published:** 2023-04-20

**Authors:** Sarah Shaw, Millie Barrett, Calum Shand, Cyrus Cooper, Sarah Crozier, Dianna Smith, Mary Barker, Christina Vogel

**Affiliations:** ^1^ MRC Lifecourse Epidemiology Centre University of Southampton Southampton SO16 6YD UK; ^2^ NIHR Southampton Biomedical Research Centre University of Southampton and University Hospital Southampton NHS Foundation Trust SO16 6YD UK; ^3^ Centre for Food Policy, City University of London Northampton Square London EC1V0HB UK; ^4^ NIHR Applied Research Collaboration Wessex, Southampton Science Park, Innovation Centre 2 Venture Road, Chilworth Southampton SO16 7NP UK; ^5^ Geography and Environmental Science University of Southampton Southampton SO17 1BJ UK; ^6^ School of Health Sciences Faculty of Environmental and Life Sciences University of Southampton Southampton SO17 1BJ UK

**Keywords:** adolescents, community nutrition environment, consumer nutrition environment, diet

## Abstract

Adolescence is a period of increased autonomy over decision‐making, including food choices, and increased exposure to influences outside the home, including the food environment. This review aims to synthesize the evidence for the influence of community nutrition environments, spatial access to food outlets, and consumer nutrition environments, environments inside food outlets, on adolescent food purchasing and dietary behaviors in high‐income countries. Six databases were searched for articles published before January 2023. Results were synthesized using a vote‐counting technique and effect direction plots that record the direction of the effect in relation to the anticipated relationship with health. Thirty‐four observational and two intervention studies met the inclusion criteria. In the 13 studies assessing adolescent exposure to healthy community nutrition environments, results did not show clear associations with dietary and purchasing outcomes. Thirty studies assessed adolescents' exposure to unhealthy community nutrition environments with the majority (n = 17/30, 57%) reporting results showing that greater exposure to food outlets classified as unhealthy was associated with less healthy food purchases and dietary intakes. Inconsistent results were observed across the seven studies investigating associations with the consumer environment. Further research in these areas, including more high‐quality intervention studies, may help to develop policy strategies to improve adolescents' dietary behaviors.

**Abbreviations**
PRISMA: Preferred Reporting Items for Systematic Reviews and Meta‐Analyses
GIS: Geographic Information System
GPS: Global Positioning System

## 1 INTRODUCTION

Adolescents have been shown to have poorer quality diets compared with other age groups, and dietary behaviors in this age group have been shown to worsen in the approach to adulthood.^1^, ^2^ Health behaviors established during adolescence have been shown to track into adulthood.^3^, ^4^ Consuming an unhealthy diet during this period of life, therefore, has the potential to negatively impact not only the immediate and future health of the individual but also the health of their future offspring.^5^ During adolescence, individuals become less dependent on family and experience increased exposure to, and influence from, environments outside of the home. Increased levels of independence also mean they have greater ability to make their own decisions about the food they eat.^6^

According to Glanz et al., the food environment consists of four different environment types, two of which are the community nutrition environment and the consumer nutrition environment.^7^ The community nutrition environment (henceforth referred to as community environment) refers to the location and accessibility of food outlets, while the consumer nutrition environment (henceforth referred to as consumer environment) refers to factors that consumers may encounter within food outlets such as food availability, price, promotions, and placement.^7^ Previous research has shown that adolescents consume the majority of their food in the home and at school;^8^, ^9^ however, when outside of their home and school environments, adolescents are more likely to consume foods that are high in fat, salt, and sugar.^10^, ^11^ Stimuli present in the community and consumer environments where food decisions are occurring are therefore likely to be playing a considerable role in influencing the independent food choices of this age group.

A number of systematic reviews have aimed to understand the influence these environments have on child and adolescent obesity, but few have examined their influence on the dietary behaviors of adolescents. Previous systematic reviews investigating the presence of food outlets around schools, mainly fast‐food outlets, convenience stores, and supermarkets, have shown little evidence for an association between outlet density or proximity and food consumption,^12^ and weak evidence of a link with body weight and obesity.^12^, ^13^ Systematic reviews investigating exposure to supermarkets and convenience stores beyond the school vicinity have also shown mixed results for an association with child and adolescent obesity.^14^, ^15^, ^16^ One systematic review, published in 2014, collated the evidence from studies investigating relationships between community and consumer environment exposures and the diets of children aged up to 18 years.^17^ The review findings showed there is moderate evidence for community and consumer environments affecting children's diets. However, it is not possible to distinguish the influence of these two nutrition environments on the diets of adolescents separately from their impact on younger children. Adolescents are likely to use these environments in different ways to that of young children because they have greater autonomy, independence of movement, and financial decision‐making. There is limited understanding about adolescents' independent food purchasing decisions, yet these autonomous food choices may play an important role in their overall dietary intake. Greater understanding of how community and consumer environments affect adolescents' food purchasing decisions could help to identify target points for future interventions to support this age group make more healthful food choices.

This systematic review aims to synthesize the evidence for relationships between community and consumer environment exposures and adolescents' diet and food purchasing behaviors. By doing so, this review will provide a better understanding of adolescents' interaction with these nutrition environments during this key life stage.

This systematic review aimed to answer two research questions:

1. How do the community and consumer nutrition environments influence adolescents' dietary behaviors?
2. How do the community and consumer nutrition environments influence adolescents' food purchasing behaviors?

## 2 METHODS

This systematic review was conducted in accordance with the 2020 guidelines from the Preferred Reporting Items for Systematic Reviews and Meta‐Analyses (PRISMA) group.^18^ The PRISMA checklist can be found in Supplementary Table 1. This systematic review was registered with the Prospective Register for Systematic Reviews (PROSPERO): CRD42019156500.

### 2.1 Search strategy

The search strategy was developed in consultation with a trained medical research librarian at the University of Southampton, UK. Six electronic databases were searched (Medline (Ovid), PsycINFO (EbscoHost), CINAHL (EbscoHost), Econlit (EbscoHost), Scopus, and GEOBASE). These databases were selected as they cover health, consumer, and geographic marketing/economic literature. The search was modified for each database to include controlled vocabulary but remained similar across databases. The search included a combination of medical subject headings (MeSH) and free‐text terms relating to “diet”, “food purchasing”, “adolescents”, “food outlets”, and “food environments”. The full search strategy can be found in Supplementary Table 2. Databases were searched for studies published in English between January 1995 and August 2021. The search was later updated to capture studies published between August 2021 and January 2023. The year 1995 was chosen as the cut‐point for the search strategy to correspond with the 2014 systematic review by Engler‐Stinger et al., which noted that most evidence in this field was collected after this time point.^17^ In 2005, Glanz and colleagues were among the first to describe the relationships between different types of food environments, diet, and health.^7^ The search strategy for this study is, therefore, likely to capture the majority of published literature in this area.

All titles and abstracts were screened independently by two authors (SS and MB) using Rayyan systematic review software^19^ against the study inclusion/exclusion criteria (Table 1). Observational and intervention studies were included if they involved adolescent participants (mean age between 11 and 18 years reflecting the age of adolescents in secondary education in the UK and other high‐income countries^20^, ^21^), were conducted in high‐income countries, included an exposure/intervention that investigated either the community or consumer environments, and had an outcome relating to food purchasing or dietary intake. If it was not clear whether a study should be included from reading the title and abstract, the full paper was assessed for eligibility.

**TABLE 1:** Inclusion/exclusion criteria for the systematic review.

	Inclusion	Exclusion
Population	Adolescents of secondary school age 11–18 years Mean age of sample falls within the age group of interest	Hospitalized adolescents Clinical populations
Setting	High‐income countries Real‐life environments	Lab settings Online food environments (Online delivery services, online supermarkets/food stores)
Study Design	Peer‐reviewed literature Empirical studies Observational studies Cross‐sectional & longitudinal studies Intervention studies including natural experiments Mixed‐methods studies (quantitative component only)	Gray literature, research thesis Qualitative literature Review and systematic review articles Editorials and commentaries Abstract only articles
Publication Dates	1995–January 3, 2023	
Exposure	Community nutrition environments as defined by Glanz et al. (spatial access to food outlets — e.g., number, density, proximity measured by GIS and GPS) Consumer nutrition environments as defined by Glanz et al. (price, availability, promotions, and placement) Objective measures of the community or consumer nutrition environment	Organizational nutrition environments (schools, home, work) Information environment (media, advertising) Consumer nutrition environment focused solely on calorie labeling Perceived/self‐report measures of the community or consumer nutrition environment
Outcomes	Food and beverage consumption Dietary indices and patterns Food frequency questionnaires 24‐hour recall Dietary screeners assessing consumption of specific food and beverage items Food and beverage purchasing Self‐reported purchases of food and beverages made by adolescents Observed purchases of food and beverages made by adolescents	Alcohol consumption or purchasing Store level sales data Household level purchasing data
Language	English language	

Abbreviations: GIS, Geographic Information System; GPS, Global Positioning System.

### 2.2 Data extraction and risk of bias assessment

Data extraction forms were created to capture relevant information to address the research questions. Two researchers reviewed each article included in the full‐text screening and subsequently completed data extraction (if applicable). During this stage, SS reviewed all papers, and MB and CS each reviewed 50% of the papers. When conflicts occurred, a third author was consulted, and agreement was reached. Details about the study design, setting, participant details, exposures/intervention, outcomes, results, and funding sources were extracted for each study. Forward and backward reference searching was conducted on the articles included in the full‐text screening stage to identify potentially relevant studies not included in the database search. When an article was considered to include inadequate detail to be included in the review, the authors were contacted to provide more information; however, none of the contacted authors responded to this request.

Concurrent with the data extraction process, each study was assessed for its risk of bias in relation to the research questions. Prior to the screening process, assessment criteria were developed specifically for this review to ensure criteria relevant to the review's research question were assessed, and were based on guidance from the Centre for Reviews and Dissemination.^22^ Risk of bias criteria were developed to assess elements of study design, participant recruitment and retention, exposure/intervention and outcome methodologies, statistical analyses, and handling of confounding. Separate criteria were created for observational (Supplementary Table 3) and intervention studies (Supplementary Table 4). A risk of bias score of +1 (low risk of bias/high quality), 0 (medium risk of bias/moderate quality), or −1 (high risk of bias/low quality) was allocated for each domain. Overall, risk of bias summaries were created for each study based on the number of “−1” ratings a study received. Studies with 5 or more, “−1” ratings were classed as having a high overall risk of bias. If the number of “−1” scores was 1 or less, the overall risk of bias was classified as low. Studies with “−1” ratings between these scores were considered to have moderate risk of bias. Reviewers compared their risk of bias ratings to ensure consistency. Any discrepancies were discussed in depth until a quality score was agreed. Final risk of bias scores for each study can be found in Supplementary Tables 5 & 6. No studies were excluded from the data synthesis based on the risk of bias rating in order to provide a complete overview of the current published research in this field.

### 2.3 Data synthesis

Because of the heterogeneity of the exposure and outcome variables used in studies included in this review, it was not possible to synthesize results using meta‐analysis. To provide a quantitative summary of the evidence for this field of research, results were synthesized using a “vote‐counting” technique based on the direction of the effect according to the expected association with health. As recommended by Cochrane for accurate vote‐counting, each study's effect estimates were categorized according to their direction in terms of showing a health benefit or harm, therefore, producing a standardized binary scoring metric.^23^ To enable this vote‐counting process, all food outlets considered to be part of the community environment in the included studies were classified as: 1) having a positive effect on diet/health, 2) having a negative effect on diet/health, or 3) a neutral effect on diet/health. These classifications were based on the ratings provided as part of two Australian‐based Delphi studies wherein participants classified the healthfulness of a wide range of food outlets.^24^, ^25^ The food outlets categorized as healthy (supermarkets) and unhealthy (fast‐food outlets, takeaways, convenience stores, petrol stores, specialty stores for discretionary foods, and variety stores) have been consistently categorized in a similar way in previous research conducted across high‐income countries^26^, ^27^, ^28^ and was, therefore, considered an appropriate approach to synthesizing existing evidence in this field. Food outlet definitions varied between studies, each outlet type was, therefore, classified according to the details provided in the text. For example, if a study had a category called “restaurants”, which they stated included fast‐food outlets, this group was categorized under fast‐food outlets for this review. Some studies assessed the exposure to a combination of healthy and unhealthy food environments as a single variable. For these studies, the combined exposure was classified as an unhealthy environment for data synthesis. This approach was adopted because previous research conducted with 839 women in the UK found that 99% lived in areas that were classified as having an unhealthy community environment when a range of food outlets were considered.^29^ For studies assessing the consumer environment, exposures relating to healthy food items (e.g., availability of fruit and vegetables) were classified as having a positive effect on diet/health, while factors relating to unhealthy food items (e.g., reduced price of fast food) were classified as having a negative effect on diet/health.

Results that were relevant to the review's research questions and had an exposure judged as having a positive or negative effect on diet/health were subsequently rated as either being “in the expected direction” or “not in the expected direction” for health benefit (Supplementary Tables 9 and 10). Expected directions could not be assigned to the studies with neutral exposures. Some studies had multiple results relevant to the review's research questions, and all results were rated separately. Results were classified as “in the expected direction” if:

- A food environment (community or consumer) classified to be healthy was associated with the purchase or consumption of healthy food, and
- A food environment (community or consumer) classified to be unhealthy was associated with the purchase or consumption of unhealthy food.

Results were classified as “not in the expected direction” if:

- A food environment (community or consumer) classified to be healthy was associated with the purchase or consumption of unhealthy food, and
- A food environment (community or consumer) classified to be unhealthy was associated with the purchase or consumption of healthy food.

Each result was further classified according to the significance level (significant *p* ≤ 0.05 or non‐significant *p* > 0.05).

These results were summarized visually using bar charts, with the details collated into effect direction plots, as recommended by Cochrane.^23^ Effect direction plots visualize the overall direction of the findings from each study. Following published instructions from Boon et al.,^30^ results that had similar exposures (community or consumer) and outcomes (diet or purchasing) were combined to demonstrate the overall direction of findings that were similar in nature. Arrows were used in the effect direction plots to represent the combined direction of results for each study. The method of combining results was based on the following criteria^30^, ^31^:

- When ≥70% of outcomes (a clear majority) reported similar directions, the arrows (▲ (positive) or ▼ (negative)) were used to represent the overall direction of effects.
- When <70% of outcomes reported similar direction, a two‐way arrow (↔) was used to represent inconsistent results.

Separate effect direction plots were created for community and consumer environment exposures. For studies that presented sub‐analysis results (e.g., boys vs girls) alongside results for the combined population, only the results for the combined population were included in the vote‐counting analyses and effect direction plots. In line with most recent guidance for generating effect direction plots, statistical significance was not included in the plots.^30^ The vote‐counting exercise was completed by the lead author (SS) and check by another author (MB or CS) who had complete the data extraction for that included study.

## 3 RESULTS

### 3.1 Search results

**FIGURE 1:**
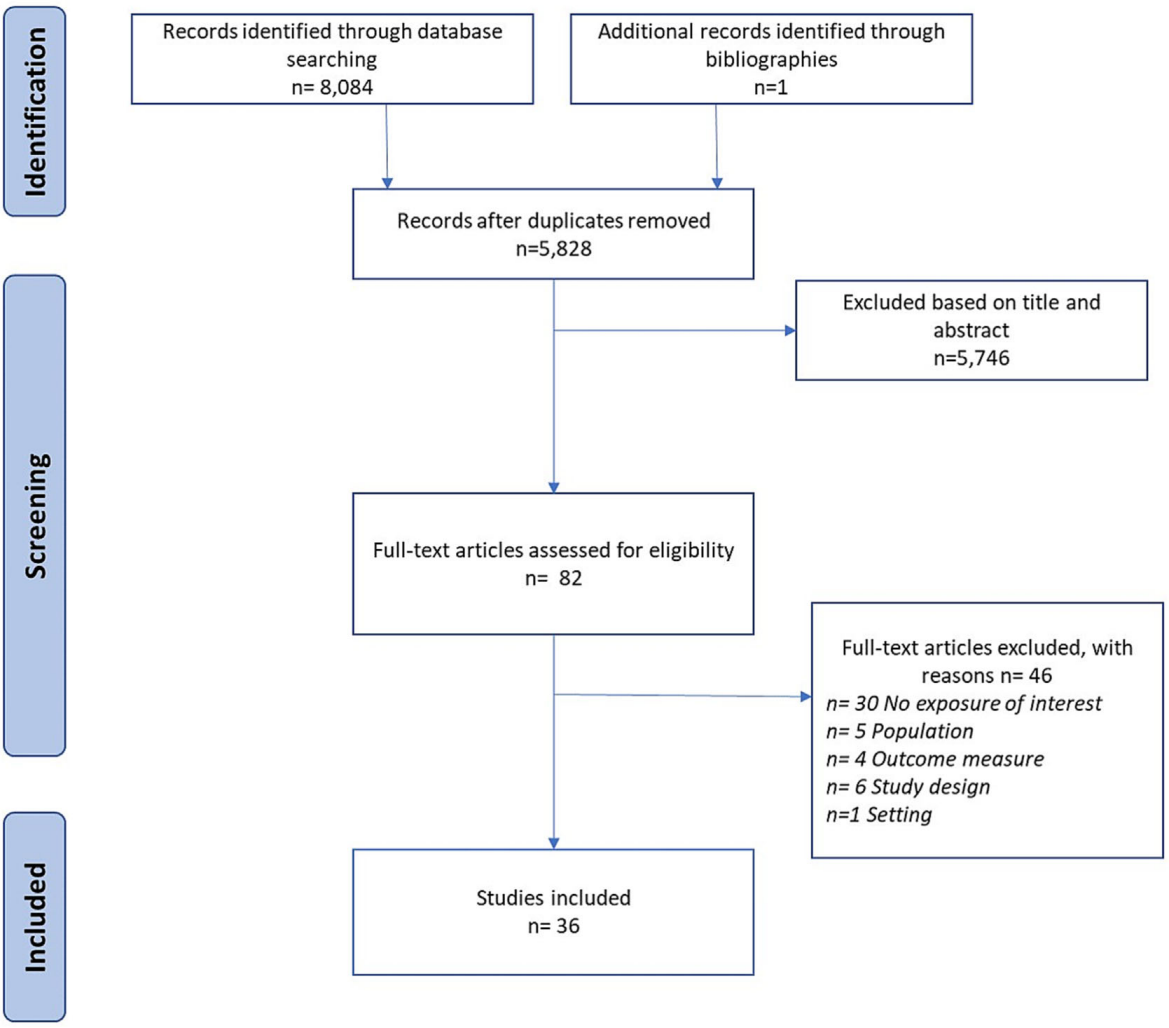
PRISMA diagram.

Figure 1 presents the PRISMA Diagram that outlines the study selection process. Following duplicate removal, 5,828 articles of potential relevance to the research questions were identified. After all titles and abstracts were screened, 82 full‐text articles were reviewed for eligibility. Thirty‐six articles met the inclusion criteria.

### 3.2 Study characteristics

Almost all studies meeting the inclusion criteria adopted an observational study design, with the majority using a cross‐sectional design (n = 31, 91%) and three having a longitudinal design (n = 3, 9%). Two intervention studies met the inclusion criteria.

The included studies were published between 2001 and 2022. Most studies included both boys and girls; one study only had girl participants and two studies recruited only boys. Almost half of all included studies were conducted in the USA (n = 17, 47%) and Canada (n = 8, 24%). Table 2 summarizes the exposures/interventions and outcomes for each study. More in‐depth details on study design, setting, participant demographics, exposures, and outcomes for each study can be found in Supplementary Tables 7 and 8.

**TABLE 2:** Exposures and outcomes for included studies.

Author, year, country	Exposure/intervention	Outcome
Intervention (Consumer)		
Lawman et al., 2015^32^ USA	12‐month CS intervention to increase availability of 4 new healthy foods (2 new products from 2 different groups: fresh F&V, canned/dried F&V, low fat diary, lean meats, whole grains) plus staff training and a marketing campaign consisting of window, door, and in‐store banners, shelf labels, and recipe cards.	Change in energy content (kcal) of foods purchased. Data collected through bag audits at store exit.
Shin et al., 2015^33^ USA	8‐month intervention aiming to increase the availability of healthy foods (healthy beverages, breakfasts, lunches, snacks, and takeaway foods) in CS and takeaways within 0.5 miles of the study recreation centers	Self‐reported change in frequency of healthful food purchases and unhealthful food purchases
Observational (Community)		
An and Sturm, 2012^34^;USA	Number of FF outlets, CS, small food stores, grocery stores, and large supermarkets within 0.5‐mile radial buffer of home and school	Self‐reported consumption of F&V, 100% juice, SSBs, high‐sugar food, and FF in previous day
Berge et al., 2014^35^; USA	1) Presence of FF outlets within 1200 m from home; 2) High density of FF outlets (5+) within 1600 m from home, 3) Presence of CS within 1200 m from home;4) Presence of supermarket within 2400 m from home; 5) Unsupportive built food environment (high density (5+) of FF outlets, presence of FF outlets and CS within 1200 m, and no nearby supermarket)	Mean consumption of FF and F&V per week
Clark et al., 2014^36^; New Zealand	1) Density of food outlets within 800 m or 1500 m radial buffer around schools; 2) Distance to nearest food outlet from school	Diet Quality Index. Higher scores reflecting greater adherence to healthy eating guidelines
Cutumisu et al., 2016^37^; Canada	Number of FF outlets within a 750 m radius street network distance buffer around school	Self‐reported “junk food” consumption at lunchtime during the previous school week
Davis and Carpenter, 2009^38^; USA	Presence (Yes/No) of a FF outlet within half a mile of school	Self‐reported consumption in the last 24 h of F&V, juice, SSBs, and fried potato. Self‐reported servings in the last 24 h of F&V, juice, SSBs, and fried potato
Forsyth et al., 2012^39^; USA	Number of FF outlets within 1600 m buffer from home and 800 m buffer from school	Frequency of eating from five categories of FF outlets: burgers and fries, fried chicken, Mexican, pizza, and sandwich restaurant
Godin et al., 2018^40^; Canada	Presence of 1 + restaurant/FF outlet, variety store, food store within 1 km circular buffer around school	Self‐reported consumption of SSBs during a usual school week (Monday–Friday)
Grier and Davis, 2013^41^; USA	Distance from school to nearest FF outlets	Self‐reported consumption of SSBs in previous day
Hager et al., 2017^42^; USA	Home located in an area with no supermarket (or healthy supermarket alternatives) within 0.4 km combined with low‐income SES factors. Home located in an area with >4 CS within 0.4 km	Self‐reported daily consumption of foods high in fat, salt and sugar
He et al., 2012a^43^; Canada	Density of “junk food” outlets: number of FF outlets and CS within 1 km buffer of home and school. Proximity of “junk food” outlet from home and school: shortest distance to nearest FF outlets and CS	FF purchasing when alone or with friends FF purchasing with parents CS purchasing when alone or with friends CS purchasing with parents
He et al., 2012b^44^; Canada	Distance from home/school to nearest CS, FF outlets, and supermarket. Number of FF outlets in 1 km buffer from home/school	Diet quality assessed using modified Healthy Eating Index‐2005 score
Jago et al., 2007^45^; USA	Distance from participants home to nearest FF outlets and small food stores	Consumption of fruit and fruit juice; low‐fat vegetables; high‐fat vegetables (fried potatoes, coleslaw, potato salad)
Kelly et al., 2019^46^; Ireland	High FF outlet density: 10% or more of food outlets in 1 km radius of school are FF outlets	Self‐reported daily consumption of F&V, sweets, SSBs, and chips
Laska et al., 2010^47^; USA	Distance to and density (number of stores within a specified buffer) of food outlets around the participants' homes and schools.	Self‐reported daily consumption of SSBs
Laxer and Janssen, 2013^48^; Canada	Density (per km^2^) of FF outlets in 1 km circular buffer from school	Non‐excessive consumption of FF = < 2 times per week Excessive FF consumption = 2 + times per week
Loh et al., 2022^49^; Australia	4 neighborhood typologies surrounding home identified through Latent Class Analysis: 1): Limited variety/low number of food outlets, 2) Some variety/low number of food outlets, 3) High variety/medium number of outlets, 4) High variety/high number of food outlets	Purchasing snack foods on journey to and from school (once a week or more, less than once a week)
Longacre et al., 2012^50^; USA	Number of FF outlets in participants town	Self‐reported consumption of FF in the previous 7 days (Yes/No)
Powell and Han, 2011^51^; USA	Availability of food outlets (FF outlets, full‐service restaurants, supermarkets, and grocery stores, CS) per 10,000 capita per 10 mile^2^	Number of days in previous week, when consumption of 7 food groups occurred: fruit and fruit juice, vegetables, meat, non‐meat protein, dairy, grains, sweets, or desserts
Sadler et al., 2016^52^; Canada	Time (minutes) exposed (within 50 m) to FF outlets, variety stores, pizza outlets, or ice cream shops on journeys to and from school	Self‐reported junk food purchasing during journey to or from school
Seliske et al., 2013^53^; Canada	Number of food retailers within 1 km of school	Regular purchasing of lunch from snack bar, FF outlet, or café on school days
Shareck et al., 2017^54^; UK	Total number of FF outlets and CS around home and/or school Proportion of all food outlets that are FF outlets and CS around home and/or school	Self‐reported weekly frequency of FF intake Daily SSB consumption
Shearer et al., 2015^55^; Canada	Average distance to each type of food outlet from home and school	Self‐reported caloric intake; diet quality; F&V consumption; frequency of FF consumption; frequency of ready‐made food consumption
Shier et al., 2016^56^; USA	Number FF outlets, CS, restaurants, small grocery stores, and supermarkets within 2‐mile radius of home address	Self‐reported weekly consumption of foods high in fat, salt, and sugar
Smith et al., 2013^57^; UK	Total number of food outlets within 400 m and 800 m road network buffer from school; Median distance to grocery store or takeaway within 400 m and 800 m buffer; Minimum distance to a grocery store or takeaway	Healthy diet score: consumption of breakfast, fruit, and vegetables. Unhealthy diet score: daily consumption of crisps and savory snacks, sweets and chocolate, biscuits; fried foods and fizzy drinks
Svastisalee et al., 2012^58^; Denmark	Number of supermarkets or FF outlets divided by total road segments within 300 m from school	Self‐reported frequency of F&V consumption
Svastisalee et al., 2015^59^; Denmark	Number of FF outlets within 500 m radius of school	Self‐reported weekly consumption of FF
Timperio et al., 2018^60^; Australia	Neighborhood typologies surrounding home identified through Latent Class Analysis. Typologies describe area consisting mainly of 1) a variety of outlets including staple/fresh food; 2) café, restaurants, takeaways, and CS; 3) very few outlets	Healthful dietary pattern: higher consumption of fruit, dried fruit, vegetables, reduced fat milk, and water as well as lower consumption of unhealthy items Energy‐dense pattern: higher consumption of energy dense sweet and savory food, and energy‐dense beverages
Trapp et al., 2022.^61^ Australia	Total number of 6 food outlet categories within 400 m, 800 m, and 1 km radial buffer of school	Frequency of purchasing snack foods (soft drinks, energy drinks, cakes/biscuits, chocolate, crisps/chips, hot chips, burgers, sausage rolls, pies)
van der Horst et al., 2008^62^;The Netherlands	Availability (total number) of FF outlets, supermarkets, CS, bakeries, and F&V and vegetable stores in 500 m radius from school; distance to nearest food store	Self‐reported liters of SSB consumed per day
Virtanen et al., 2015^63^; Finland	Shortest straight‐line distance to a FFO outlet or supermarket from school	Self‐reported purchasing of snacks (not main meals) from outside school
Observational (Consumer)		
Edmonds et al., 2001^64^; USA	Mean availability of F&V, and 100% fruit juice in census tract. Assessed by store audits.	Mean consumption of F&V, and 100% fruit juice for participants living in census tract
Gustafson et al., 2017^65^; USA	Combined Nutrition Environment Measure Survey (NEMS) scores assessing the quality, availability, and price of healthy foods in the three most frequently visited food outlets. Higher scores = healthier environments	Self‐reported daily intake of F&V, added sugar, and SSBs
Khan et al., 2012^66^; USA	Index of FF price computed for closest zip code using three food items in the American Chambers of Commerce Researchers Association	Self‐reported number of days in past week FF was consumed
Powell and Han, 2011^51^; USA	Index of FF price computed for closest zip code using three food items in the American Chambers of Commerce Researchers Association	Number of days in previous week, when consumption of seven food groups occurred: fruit and fruit juice, vegetables, meat, non‐meat protein, dairy, grains, sweets, or desserts
Sturm and Datar, 2011^67^; USA	Price indices (standardized) for F&V, dairy, and FF at metropolitan area	Consumption in the previous week of F&V, milk, soft drinks, and FF

Abbreviations: CS, convenience stores; FF, fast food; F&V, fruits and vegetables; SSB, sugar‐sweetened beverages.

### 3.3 Exposures/interventions

#### 3.3.1 Community nutrition environment

Thirty studies, all with observational designs, assessed the community environment. No intervention studies assessed the community environment.

Density, the number of food outlets in a specific geographic area, was assessed in 22 studies.^34^, ^35^, ^36^, ^37^, ^39^, ^42^, ^43^, ^44^, ^46^, ^47^, ^48^, ^49^, ^50^, ^51^, ^53^, ^54^, ^56^, ^58^, ^59^, ^60^, ^61^, ^62^ Proximity, the distance to food outlets, was assessed in 14 studies.^35^, ^36^, ^38^, ^40^, ^41^, ^42^, ^43^, ^44^, ^45^, ^47^, ^55^, ^57^, ^62^, ^63^ One study assessed the time spent exposed to unhealthy food outlets on the journey to and from school.^52^

Geographic Information System (GIS) methods were used to assess either density or proximity measures of the food environment in 28 studies. The methods used in these GIS studies varied. Both Euclidean and street network distances were applied, with buffers ranging from 300 m to 3200 m, around either the participants' homes and/or schools.^56^ Global Positioning System (GPS) methods were used in two studies to generate personalized activity spaces for study participants.^52^, ^55^ Ground‐truthing (on‐site, in‐person verification of food outlets) of fast‐food outlets in participants' towns was completed by one study,^50^ and another used zip‐code data to consider the number of food outlets present per 10,000 capita per 10 mile^2^.^51^

Food outlet data were gathered using government/local authority databases in 10 studies, business directories in 12 studies, and phone directories or online searches in seven studies. One study used food environment data collected by the Baltimore City Food Policy Initiative as part of a larger, citywide project.^42^ Few studies validated the presence of the food outlet exposures. Of the eight that did, three conducted physical ground‐truthing^42^, ^50^, ^58^ while five others used telephone calls, internet searches, and knowledge of local residents to verify the location or presence of food outlets.^36^, ^43^, ^44^, ^55^, ^59^

The majority of studies that assessed the community environment used absolute measures, such as the total number of a particular food outlet in a given area. Four studies considered relative measures that combine more than one element of the food environment (e.g., different food outlet types). One study created an “unsupportive built food environment” variable for areas that contained a high density (5+) of fast‐food outlets within 1,600 m from home combined with no nearby supermarket and at least one fast‐food outlet and convenience store within 1,200 m of home.^35^ Two studies assessed the proportion of fast‐food outlets^46^, ^54^ or convenience stores to the total number of food outlets around schools.^54^ Two studies used Latent Class Analysis to identify typologies that best described the combination of food outlets in the neighborhoods surrounding participants' homes.^49^, ^60^

#### 3.3.2 Consumer nutrition environment

Seven studies, five with observational and two with intervention designs, assessed aspects of the consumer environment in relation to adolescents' food purchases and dietary behaviors. Two observational studies assessed the availability of healthy food items in supermarkets, convenience stores, and restaurants. The Nutrition Environment Measure Survey (NEMS) tool was used in one of these studies to assess healthy food availability in the three food outlets most frequently visited by their adolescent participants.^65^ The other study calculated the mean fruit and vegetable availability for each participant's local area by conducting in‐store audits in one randomly selected supermarket and one convenience store per census tract to determine the presence and shelf space in meters of fruit and vegetables.^64^

Three observational studies assessed the price of food items in relation to adolescent food purchasing and dietary behaviors. Two of these studies created price indices for fast‐food in the participants' residential postcode areas by using the mean prices for three key indicator fast‐food items (a quarter‐pound burger with cheese, a thin crust regular cheese pizza, and a fried chicken thigh or drumstick from leading fast‐food outlet chains).^51^, ^66^ The third study created a price index using the United States Cost of Living Index data to reflect relative food prices for each participant's metropolitan area, which consisted of the average annual prices for fruit, vegetables, dairy, and fast‐food divided by the overall Cost of Living Index for the area.^67^

Both intervention studies manipulated the consumer environment by increasing the availability of healthy foods and introducing a range of marketing materials and activities, such as taste testing, to promote the healthy items.^32^, ^33^

### 3.4 Outcomes

Most (n = 28, 78%) of the included studies considered a dietary variable as the outcome of interest while eight (22%) studies used a purchasing variable as the outcome. The majority of studies using dietary outcomes assessed the consumption of individual food groups, such as fruit, vegetables, sugar‐sweetened beverages (SSBs), fast‐food, and foods high in fat, salt, and sugar through self‐report questionnaires. Five studies used composite measures to assess adolescents' overall dietary intake. Diet quality was assessed in three of these studies using different tools including the validated New Zealand Diet Quality Index for Adolescents,^36^ a modified version of the Healthy Eating Index 2005,^44^ and the International Dietary Quality Index.^55^ Two studies did not report validation details of the composite measures. One study derived a healthy diet score based on responses about breakfast and daily fruit and vegetable consumption, plus an unhealthy diet score based on the consumption of crisps and savory snacks, sweets or chocolate, biscuits, fried foods, and fizzy drinks.^57^ Another study used principal component analysis to create a healthful and an energy‐dense dietary pattern score using food frequency questionnaire data from participants.^60^

Eight studies, including the two intervention studies, assessed adolescent purchases. Each study used a different methodology and focused on the purchasing of different types of food. Seven studies collected self‐reported details about food purchasing; six used questionnaires^33^, ^43^, ^49^, ^53^, ^61^, ^63^ and one used daily activity diaries.^52^ The final study directly observed customer purchases at convenience stores and calculated the total energy content of the purchases made.^32^

### 3.5 Risk of bias

For the observational studies, the majority (n = 25, 74%) were considered to have an overall “moderate risk of bias” in relation to the research questions. Two observational studies (6%) were rated as having a “low risk of bias” and seven (22%) were rated as having a “high risk of bias”. The two intervention studies were considered to have a “high risk of bias’ in relation to the research question.

### 3.6 Key findings

**FIGURE 2:**
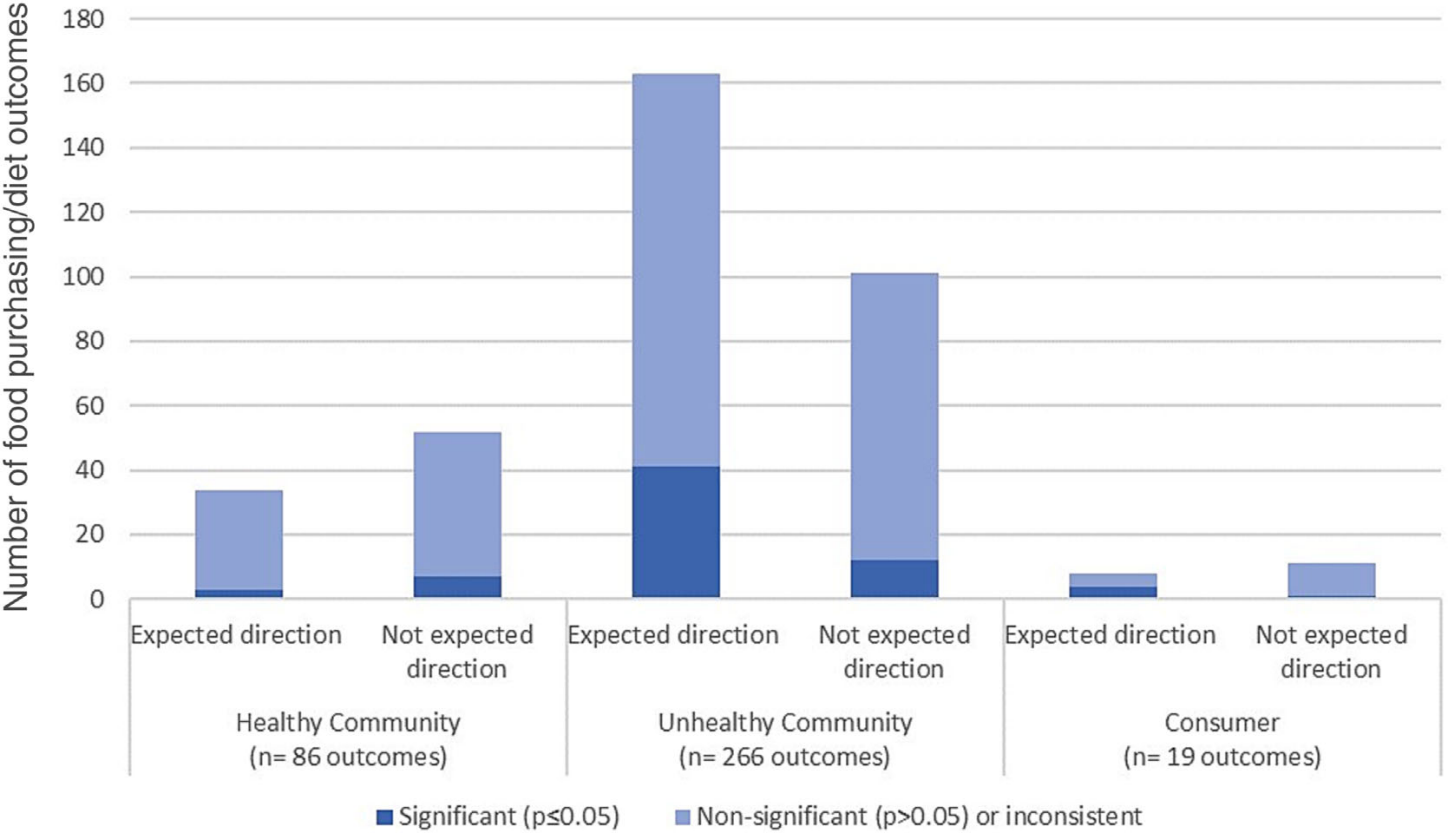
Graph showing the vote‐counting results for the associations between the community and consumer nutrition environments and diet‐related behaviors.

Figure 2 summarizes the vote‐counting results for the studies that considered healthy community environment exposures and unhealthy community environment exposures as well as total consumer environment exposures. In total, 352 food purchasing and diet outcomes were recorded from studies with community environment exposure and 19 outcomes from studies with consumer environment exposures. For the community environment exposures, the results are presented separately for healthy and unhealthy community environments; with the majority (76%) falling in the unhealthy community environment.

For vote‐counting results related to healthy community environment exposures, more than half (60%, n = 52/86) did not support the review hypothesis that exposure to healthy food outlets was associated with increased purchasing and consumption of healthy foods. A small majority of findings (62%, n = 165/266) relating to unhealthy community environments supported the review hypothesis that exposure to unhealthy food outlets was associated with increased purchasing and consumption of unhealthy foods. The majority of findings (82%, n = 287/352) relating to the community environment were not statistically significant.

For vote‐counting, the consumer environment results combined healthy and unhealthy consumer environments because the body of evidence investigating these exposures was small. Overall, 58% (n = 11/19) were not in the expected direction and did not support the review hypothesis that greater exposure to healthy environments in food outlets is associated with increased purchasing and consumption of healthy foods. Again, the majority (79%, n = 15/19) of findings relating to the consumer environment were not statistically significant (i.e., *p* > 0.05).

#### 3.6.1 Community nutrition environments

Table 3 shows the effect direction plot for the studies that considered the community environment as an exposure. Healthy community environments (i.e., exposure to supermarkets) were considered in 13 studies, 12 with dietary outcomes and one with purchasing outcomes. Of the studies assessing diet, n = 4/12 (33%) reported combined results not in the expected direction,^44^, ^55^, ^57^, ^62^ indicating that greater exposure to supermarkets and grocery stores was associated with poorer dietary outcomes in adolescents. Three studies (n = 3/12, 25%) reported results in the expected direction.^56^, ^58^ Five studies (n = 5/12, 42%) reported inconsistent results between exposure to healthy food outlets around home and/or school and adolescents' dietary intakes.^34^, ^36^, ^42^, ^47^ In the single study that assessed purchasing, overall findings from that study were in the unexpected direction suggesting that greater exposure to more healthful environments was associated with increased unhealthy snack purchases.^61^ Six of the studies assessing healthy community environments (6/13, 46%) were conducted in the USA, with two (n = 2/6, 33%) showing results in the expected direction^35^, ^56^ and four (n = 4/6, 67%) showing inconsistent findings.^34^, ^42^, ^47^, ^51^ In the two studies from Canada, both found results in the unexpected direction, showing greater access to supermarkets was associated with less healthy dietary behaviors.^44^, ^55^

**TABLE 3:** Effect direction plot for community nutrition environment results.

Author, year	Country	Study design	Sample size	Exposure to healthy food outlets	Exposure to unhealthy food outlets	Outcome	Risk of bias
An and Sturm, 2012^34^	USA	Obs (CS)	5236	↔^36^	↔^54^	Diet	Moderate
Berge et al., 2014^35^	USA	Obs (CS)	2682	▲^4^	↔^16^	Diet	Moderate
Clark et al., 2014^36^	New Zealand	Obs (CS)	664	↔^3^	▼^9^	Diet	Low
Cutumisu et al., 2016^37^	Canada	Obs (CS)	26655		▲	Diet	Moderate
Davis and Carpenter, 2009^38^	USA	Obs (CS)	529367		▲^10^	Diet	High
Forsyth et al., 2012^39^	USA	Obs (CS)	2724		▲^6^	Diet	High
Godin et al., 2018^40^	Canada	Obs (CS)	41829		▲^3^	Diet	Moderate
Grier and Davis, 2013^41^	USA	Obs (CS)	100000		▲	Diet	High
Hager et al., 2017^42^	USA	Obs (CS)	634	↔^3^	↔^8^	Diet	Low
He et al., 2012b^44^	Canada	Obs (CS)	580	▼^2^	▲^8^	Diet	Moderate
Jago et al., 2007^45^	USA	Obs (CS)	204		▼^5^	Diet	Moderate
Kelly et al., 2019^46^	Ireland	Obs (CS)	5344		↔^5^	Diet	Moderate
Laska et al., 2010^47^	USA	Obs (CS)	349	↔^2^	▲^3^	Diet	Moderate
Laxer and Janssen, 2013^48^	Canada	Obs (CS)	6099		▲^3^	Diet	Moderate
Longacre et al., 2012^50^	USA	Obs (CS)	1547		▲^2^	Diet	Moderate
Powell and Han, 2011^51^	USA	Obs (CS)	1134	↔^3^	↔^6^	Diet	Moderate
Shareck et al., 2017^54^	UK	Obs (CS)	3089		↔^12^	Diet	Moderate
Shearer et al., 2015^55^	Canada	Obs (CS)	315	▼^10^	▲^20^	Diet	Moderate
Shier et al., 2016^56^	USA	Obs (CS)	941	▲^6^	↔^18^	Diet	Moderate
Smith et al., 2013^57^	UK	Obs (LT)	524	▼^4^	▲^5^	Diet	Moderate
Svastisalee et al., 2012^58^	Denmark	Obs (CS)	6034	▲^6^	↔^24^	Diet	Moderate
Svastisalee et al., 2016^59^	Denmark	Obs (CS)	4642		↔^2^	Diet	Moderate
Timperio et al., 2018^60^	Australia	Obs (CS & LT)	439		▲^8^	Diet	Moderate
van der Horst et al., 2008^62^	The Netherlands	Obs (CS)	1174	▼	▲^5^	Diet	High
He et al., 2012a^43^	Canada	Obs (CS)	782		▲^5^	Purchase	Moderate
Loh et al., 2022^49^	Australia	Obs (CS)	410		▼^6^	Purchase	Moderate
Sadler et al., 2016^52^	Canada	Obs (CS)	511		▲	Purchase	High
Seliske et al., 2013^53^	Canada	Obs (CS)	6971		▲^6^	Purchase	Moderate
Trapp et al., 2021^61^	Australia	Ob (CS)	2389	▼^6^	↔^12^	Purchase	Moderate
Virtanen et al., 2015^63^	Finland	Obs (CS)	23182		▲^2^	Purchase	Moderate

Effect direction: ▲ Positive result; ▼ Negative result; ↔ Inconsistent results. Number of outcomes within each category is 1 unless indicated in subscript beside effect direction.

Reported effect direction for multiple outcomes: All outcomes report effect in the same direction OR where direction of effect varies across multiple outcomes: ≥70% of outcomes report similar direction. Inconsistent findings = if <70% of outcomes report consistent direction of effect (↔).

Thirty studies assessed adolescents' exposure to unhealthy food environments in relation to their dietary and food purchasing behaviors. Majority of these studies (n = 17/30, 57%) reported results in the expected direction suggesting greater exposure to food outlets classified as unhealthy (i.e., fast food outlets, convenience stores, variety stores, unhealthy specialty stores) was associated with unhealthier food purchases and dietary intakes.^37^, ^38^, ^39^, ^40^, ^41^, ^43^, ^44^, ^47^, ^48^, ^50^, ^52^, ^53^, ^55^, ^57^, ^58^, ^60^ Diet was the primary outcome in 24 of these 30 studies. When findings were combined for studies with diet outcomes, 54% (n = 13/24) of studies showed results supporting the study hypothesis.^37^, ^38^, ^39^, ^40^, ^41^, ^44^, ^47^, ^48^, ^50^, ^55^, ^57^, ^58^, ^60^ Inconsistent results were observed in 38% (n = 9/24) of studies with dietary outcomes.^34^, ^35^, ^36^, ^42^, ^46^, ^51^, ^54^, ^56^, ^59^, ^62^ Six studies investigated unhealthy community environment exposures in relation to food purchasing outcomes. The majority of studies with purchasing outcomes (n = 4/6, 67%) reported results in the expected direction, suggesting greater exposure to unhealthy outlets is associated with a larger number of unhealthy food purchases and fewer healthy food purchases among adolescents.^43^, ^52^, ^53^, ^63^ One study showed results in the unexpected direction^49^ and one show inconsistent findings.^61^ The majority of studies assessing unhealthy community environments were conducted in the USA and Canada. Findings from studies in the USA showed mixed findings with five (n = 5/11, 45%) showing results in the expected direction,^38^, ^39^, ^41^, ^47^, ^50^ five (n = 5/11, 45%) showing inconsistent findings,^34^, ^35^, ^42^, ^51^, ^56^ and 1 (n = 1/11, 9%) showing results in the unexpected direction.^45^ Findings from studies conducted in Canada (n = 8) were consistent; all showed results in the expected direction indicating greater access to unhealthy food outlets was associated with less healthy food purchasing and dietary behaviors in adolescents.^37^, ^40^, ^43^, ^44^, ^48^, ^52^, ^53^, ^55^

Cafes and restaurants were considered in five studies but not included in the effect direction plot because they were classified as food outlets that have an overall neutral effect on health.^24^ All of these studies considered diet variables as primary outcomes. Findings from these studies suggest that greater levels of exposure to cafes and restaurants associated with better dietary behaviors in adolescents (n = 15/25 outcomes, 60%).

#### 3.6.2 Consumer nutrition environment

Inconsistent results were observed in five studies investigating associations between the consumer environment and adolescent dietary behaviors and the two intervention studies that assessed purchasing outcomes (Table 4). Findings of the two studies focused on increasing the availability of healthy foods were inconsistent; one found increased consumption of fruit but decreased consumption of vegetables,^64^ and the other found decreased consumption of fruit, vegetables, and SSBs but increased consumption of added sugar.^65^ One study investigated the influence that the price of healthy foods had on intake and found a relationship in the unexpected direction between the price of fruit and vegetables and the consumption fruit and vegetables, whereby higher prices were associated with higher intake.^67^ In three studies investigating the price of unhealthy foods,^51^, ^66^, ^67^ one (33%) showed results in the expected direction^66^; the two other studies showed results in the unexpected direction suggesting higher prices on unhealthy food items were associated with greater consumption of those items.^51^, ^67^ No overall direction could be determined from the two intervention studies that aimed to improve the healthfulness of the in‐store consumer environment in convenience stores by increasing the availability of healthy foods and using signage and instore marketing strategies (taste tests, cooking demonstrations, and recipe cards) to promote these foods. One reported a non‐significant increase in the energy content of adolescent food purchases following the intervention.^32^ The second study reported a non‐significant decrease in the purchasing of unhealthy food items but also observed a decrease in purchasing of healthy food items (non‐significant) following the intervention.^33^

**TABLE 4:** Effect direction plot for consumer nutrition environment results.

Author, year	Country	Study design	Sample size	Exposure type	Healthy consumer environment	Unhealthy consumer environment	Outcome type	Risk of bias
Observational								
Edmonds et al., 2001^64^	USA	Obs (CS)	90	Availability	↔^6^		Diet	High
Gustafson et al., 2017^65^	USA	Obs (CS)	432	Availability	↔ ^3^		Diet	Moderate
Khan et al., 2012^66^	USA	Obs (LT)	11700	Price		▲	Diet	High
Powell and Han, 2011^51^	USA	Obs (CS)	1134	Price		▼^3^	Diet	Moderate
Sturm and Datar, 2011^67^	USA	Obs (CS)	6034	Price	▲	▼^2^	Diet	Moderate
Intervention								
Lawman et al., 2015^32^	USA	Intervention (repeated CS sample with no control) 12 months	999	Availability and social marketing campaign	▼		Purchases	High
Shin et al., 2015^33^	USA	Cluster randomized intervention with control 8 months	152	Availability and in‐store promotion	↔^2^		Purchases	High

Effect direction: ▲ Positive result; ▼ Negative result; ↔ Inconsistent results. Number of outcomes within each category is 1 unless indicated in subscript beside effect direction.

Reported effect direction for multiple outcomes: All outcomes report effect in the same direction a OR where direction of effect varies across multiple outcomes: ≥70% of outcomes report similar direction. Inconsistent findings = if <70% of outcomes report consistent direction of effect (↔).

## 4 DISCUSSION

### 4.1 Summary of findings

This systematic review synthesized findings from available scientific literature and determined the overall direction of the associations between the community and consumer environments and adolescents' food purchasing and dietary behaviors. In recognition that unhealthy food environments play a role in obesity and non‐communicable disease risk, and the importance of adolescence as a key period of life to establish healthy dietary behaviors, this review highlights the need for further high‐quality intervention research. The observational evidence available summarized in this review suggests that increased adolescent exposure to unhealthy food outlets, such as fast‐food outlets and convenience stores, is associated with greater purchase and consumption of unhealthy foods. Conversely, and in contrast to one of the review's two hypotheses, exposure to food outlets categorized as healthy was not associated with greater consumption of healthy foods. Testing the relationship between exposure to healthy outlets and food purchasing/dietary outcomes may have been limited because supermarkets, the only outlets included in this review that fell in the healthy category, sell both healthy and unhealthy foods. This finding illustrates that supermarkets represent more of a mixed exposure in terms of healthfulness.

The evidence assessing the role of the consumer environment in adolescent food purchasing and dietary behaviors is limited, and further research is needed to address this evidence gap. Among the observational studies that assessed the consumer environment, no clear associations between price, availability, and adolescents' dietary intake could be determined.^51^, ^64^, ^65^, ^66^, ^67^ Only two intervention studies, both with high risk of bias, have been conducted in this field. These two studies manipulated the in‐store environment of convenience stores to improve the availability and non‐price related promotion of healthy foods. No clear direction of effect was observed on the healthfulness of adolescent food purchases from these intervention studies. These studies are of particular interest as previous systematic reviews of literature among adults have shown that consumer environment factors, such as in‐store placement, price, and promotion strategies, have a stronger, more consistent influence on the food purchasing and dietary behaviors of adults.^31^, ^68^, ^69^, ^70^, ^71^ However, evidence for the influence of consumer environment factors on adolescents' purchasing patterns is less clear, which might suggest that adolescents' food purchases are motivated by different factors than those of adults. Adults are generally purchasing food items for the household, to be consumed at home, whereas adolescents tend to purchase foods for immediate consumption, often in the presence of their friends.^72^, ^73^ A greater understanding of how consumer environments are influencing adolescents' food purchasing and dietary behavior is critical given the findings for the community environment found in this systematic review. If exposure to both healthy and unhealthy food outlet types is associated with less healthy food‐related behaviors among adolescents, understanding the factors inside these outlets that encourage these unhealthy choices would be important to inform effective public health interventions targeting this population.

This systematic review classified food outlets according to their “healthfulness” ratings from previous research^24^, ^25^ and is in line with how these food outlets are often considered in the food environment literature in terms of health.^26^, ^27^, ^28^ This approach may be overly simplistic and result in some food outlets receiving a rating that does not reflect all the food items on sale. For example, supermarkets received a “healthy” rating; however, previous studies involving in‐store audits in supermarkets, assessing food availability, variety, price, and promotions have shown supermarkets have varying levels of overall “healthfulness”.^74^, ^75^ Additionally, other research has shown that ultra‐processed foods and foods high in fat, salt, and sugar are more frequently promoted by supermarkets in western countries than foods that are supportive of healthy eating guidelines.^76^, ^77^ This review did not find evidence to support one of the hypotheses that increased exposure to supermarkets is associated with better dietary behaviors among adolescents. Further research is required to provide accurate health ratings of food outlets, particularly supermarkets, and to understand how these are associated with adolescent food purchasing and diet.

Dietary outcomes have been assessed more often than food purchasing behaviors in studies assessing the influence of the community and consumer environments adolescents' diet‐related behaviors. Similar to findings from a previous systematic review, asking about consumption of single food items or short screener‐style questionnaires were the dietary data collection methods most often used in studies in this review.^78^ The use of such methods may not be capturing the complex nature of dietary intake. Only five of the 28 included studies assessing diet (18%) considered diet as a whole by using diet quality indices and composite dietary pattern scores.^36^, ^44^, ^55^, ^57^, ^60^ In addition, this review has highlighted the gap in research investigating adolescent food purchasing. The methods used varied, but only one such study collected information about specific food and drink purchases made by adolescents via bag audits when exiting the store^32^; these data were converted into an outcome measure that described the energy content of all purchases in the shopping bag. No studies collected data about how often food purchases were made by adolescents. As a result, it was not possible to comment on the overall healthfulness of adolescent food purchases. Such evidence gaps make it difficult to assess how significant adolescent‐determined food purchases are in relation to their overall dietary intake, or whether this contribution varies with age or family socioeconomic status. Future research should explore the use of novel technologies to provide insight into the types of food purchases made by adolescents, using adolescent store loyalty cards or ecological momentary assessment, for example.

All but two of the studies in this review assessed community environment measures using GIS technology to examine participants' use of the area around the home and/or school demonstrating the growing popularity of applying GIS methodology to this field of research.^79^ Methodological limitations of GIS have been highlighted previously, with concerns including the inability of GIS to account for daily movements of individuals leading to an overestimation of the importance of the home neighbourhood.^79^ These concerns have drawn into question how best to identify the spatial exposures that accurately represents the environments important in shaping behavior.^79^, ^80^ The use of GPS to assess individuals' exposure to the food environment is thought to address some of these concerns because individualized activity spaces that reflect daily movement can be created.^79^ Only two studies in this review used GPS methods. The lack of research using GPS to assess adolescents' community environment exposures highlights an area for future research.

### 4.2 Public health implications

Further high‐quality evidence is required to support the development of public health policies that encourages healthy eating behaviors among adolescents, especially their independent food choices outside of home and school. To combat childhood obesity, some local‐level governments have taken action towards creating healthier food environments by introducing zoning or planning limits on fast‐food outlets. Examples include in Detroit, USA, where zoning legislation has prohibited fast‐food outlets opening within 500 ft of schools,^81^ and Wicklow, Ireland, where “no fry zones” limit fast‐food outlets and takeaways opening within 400 m of school sites and playgrounds.^82^ A recent census of all 325 local governments in England found that roughly half had a policy specifically targeting takeaway food outlets.^83^ Similar to the examples from Ireland and USA, the majority of the policies in England that focused on health included exclusion zones around areas relating to children and families such as schools, parks, and leisure facilities. The implementation rates and the effectiveness of these policies, however, were not reviewed.^83^ While this is a logical starting point for such policies, further research is needed to understand how adolescents use their community nutrition environments and whether focusing on the traditional areas where children and families spend time is sufficient to have a positive role in altering adolescents' independent food behaviors. An expanded policy approach is likely to be particularly important for adolescents because of their increased levels of independence resulting in them being exposed to larger spatial areas outside of the home and school surrounds.

The UK government is the first country to introduce legislation limiting food retailers' marketing strategies in retail outlets. Since October 1, 2022, most retailers can no longer place foods high in fat, sugar, and salt in prominent store positions, including store entrances, aisle ends, and checkouts. The UK government has also announced intentions to limit volume‐based promotions (e.g., buy one get one free) of these items; the law is due to come into effect on October 1, 2023.^84^, ^85^ Given that existing evidence for how consumer environments affect adolescents' food choices is limited, there is little to help us anticipate the impact of these policies on this age group. Further research in this area and adolescent‐specific evaluation of these policies will identify if further support for adolescents is required to help them make healthy food decisions in their community and consumer environments.

### 4.3 Strengths and limitations of this review

A strength of the review is the adherence to PRISMA guidelines throughout. In addition, two reviewers independently conducted data extraction and quality assessment for each of the included studies to ensure consistency and rigor. This review searched six databases that cover topics from different disciplines considered relevant to the review research question. Gray literature was not searched as it was considered outside the scope of this review, meaning research published outside of academic journals was not included. This review is the first among reviews in this field to have summarized the direction of results and provide an overview of the findings. The scoring technique used in this review to rate outlets as healthy/unhealthy was based on ratings obtained from two Australian Delphi studies and may not accurately reflect all food outlets in other high‐income countries. The categories assigned to food outlets in this review, however, are aligned with previously published categorizations of food outlets in terms of their role in promoting healthy food choices across a range of high‐income countries^13^, ^26^, ^27^, ^28^ and offers a consistent approach to synthesizing existing evidence in this field. As discussed above, the approach taken to rate supermarkets as healthy environments may be problematic because these outlets typically sell a range of healthy and unhealthy items. Future research could explore more nuanced methods to categorize supermarket environments. As with many systematic reviews, publication bias may influence the studies included and, thus, affect the review's findings. Studies reporting significant findings may have been more successful in the publication process. Not being able to include results from unpublished studies may be skewing the overall direction of the evidence in this field; however, 82% of results considered in this review were not statistically significant. This review only included studies that have used an objective measure of community or consumer environment. While this may reduce subjectivity in assessing the exposure, previous research indicates that participants' perceptions of their environments can play an important role in determining their behaviors in or near those environments.^17^ This review did not include online and digital food environments, which have been growing in popularity in recent years.^86^ These types of online environments, particularly online food delivery services, may increase adolescents access and exposure to food outlets, particularly takeaway outlets, beyond those that are in their immediate geographic vicinity. Digital food environments may, therefore, be impacting adolescents' food purchasing and dietary behaviors in ways not considered in this review.

The studies included in this review were not suitable for including in a meta‐analysis because of the heterogeneity in study design. A direction‐based vote‐counting technique was, therefore, used to provide a quantitative summary of the evidence. As recommended by Cochrane, effect direction plots were also used to combine multiple findings from the same study, based on similar exposures and outcomes, to provide an overall direction of the study findings.^87^ This technique allows for a quantitative summary of findings in addition to a narrative review and provides the reader with an overall sense of the direction of the associations on the topic. This approach, however, is limited by the fact that it does not recognize study size and the magnitude of associations investigated.

## 5 CONCLUSIONS

Adolescents' exposure to food outlets may be playing an important role in their autonomous food choices. There is moderate evidence that adolescents' exposure to food outlets categorized as unhealthy is associated with having unhealthier dietary behaviors; there is also some evidence that increased exposure to healthy food outlets may also be related to poorer dietary choices among this age group. Further research is needed to understand better how adolescents use their community nutrition environments in order to determine if policies that focus on limiting exposure to unhealthy food outlets around key locations, such as schools, are having the desired outcome. The evidence base investigating how the consumer nutrition environment affects adolescents' food choices is very limited. Further high‐quality intervention research is needed to provide insight into how factors inside food outlets can be manipulated in order to promote healthier food‐related behaviors during this key period of development.

## AUTHOR CONTRIBUTIONS

The authors' responsibilities were as follows: SS and CV contributed to the conception and design of the research. SS conducted the literature search and screened the search returns. SS, MB and CS conducted data extraction and quality appraisal. CC, SC, DS, MB and CV advised on the systematic review process. All authors gave input to the interpretation of results, writing of the manuscript and approved the final copy.

## CONFLICT OF INTEREST STATEMENT

SS, MB, CS, CC SC, DS, MB have no conflicts of interests to declare. CV has a non‐financial research collaboration with a UK supermarket chain. The study described in this manuscript is not related to this relationship.

## Supporting information


**Supplementary Table 1:** Prisma 2020 Checklist
Supplementary Table 2 Full search strategy
Supplementary Table 3 Risk of Bias Assessment Criteria for Observational Studies
Supplementary Table 4 Risk of Bias Assessment Criteria for Intervention Studies
Supplementary Table 5 Risk of bias ratings for observational studies
Supplementary Table 6 Risk of bias ratings for intervention studies
**Supplementary Table 7:** Details of included observational studies
**Supplementary Table 8:** Details of included intervention studies
**Supplementary Table 9:** Vote‐counting results for observational studies
**Supplementary Table 10:** Vote‐counting results for intervention studies

## Data Availability

The data that support the findings of this review are available from the corresponding author upon reasonable request.
