# Supplementary material for: Influences of the community and consumer nutrition environment on the food purchases and dietary behaviors of adolescents: A systematic review
Source: Obes Rev. 2023 Apr 20;24(7):e13569. doi: 10.1111/obr.13569 (PMC10909420; doi:10.1111/obr.13569)
Supplement: Supplementary file 1 — Supplementary Table 1: Prisma 2020 Checklist Supplementary Table 2 Full search strategy Supplementary Table 3 Risk of Bias Assessment Criteria for Observational Studies Supplementary Table 4 Risk of Bias Assessment Criteria for Intervention Studies Supplementary Table 5 Risk of bias ratings for observational studies Supplementary Table 6 Risk of bias ratings for intervention studies Supplementary Table 7: Details of included observational studies Supplementary Table 8: Details of included intervention studies Supplementary Table 9: Vote‐counting results for observational studies Supplementary Table 10: Vote‐counting results for intervention studies [file OBR-24-e13569-s001.pdf]

Supplementary Material

Influences of the community and consumer nutrition environment on the food purchases and dietary behaviours of adolescents: a systematic review

Sarah Shaw[1,2], Millie Barrett[1,2], Calum Shand[1], Cyrus Cooper[1,2], Sarah Crozier[1,2,3], Dianna Smith[3,4], Mary Barker[1,2,5], Christina Vogel[1,2,3,6]

Affiliations

[1] *MRC Lifecourse Epidemiology Centre, University of Southampton, Southampton, United Kingdom, SO16 6YD*

[2] *NIHR Southampton Biomedical Research Centre, University of Southampton and University Hospital Southampton NHS Foundation Trust, United Kingdom, SO16 6YD*

[3] *NIHR Applied Research Collaboration Wessex, Southampton Science Park, Innovation Centre, 2 Venture Road, Chilworth, Southampton, United Kingdom, SO16 7NP*

[4] *Geography and Environmental Science, University of Southampton. Southampton, United Kingdom. SO17 1BJ*

[5] *School of Health Sciences, Faculty of Environmental and Life Sciences, University of Southampton, Southampton, United Kingdom, SO17 1BJ*

[6] *Centre for Food Policy, City, University of London, Northampton Square, London EC1V0HB*

Corresponding Author
Sarah Shaw
MRC Lifecourse Epidemiology Centre, University of Southampton
Southampton General Hospital
Tremona Road
Southampton, SO16 6YD, UK
Email: ss@mrc.soton.ac.uk

Supplementary Table 1: Prisma 2020 Checklist

Section and Topic	Item #	Checklist item	Location where item is reported
**TITLE**			
Title	1	Identify the report as a systematic review.	Page 1
**ABSTRACT**			
Abstract	2	See the PRISMA 2020 for Abstracts checklist.	Page 2
**INTRODUCTION**			
Rationale	3	Describe the rationale for the review in the context of existing knowledge.	Pages 4-5
Objectives	4	Provide an explicit statement of the objective(s) or question(s) the review addresses.	Page 5
**METHODS**			
Eligibility criteria	5	Specify the inclusion and exclusion criteria for the review and how studies were grouped for the syntheses.	Page 5<br>Table 1
Information sources	6	Specify all databases, registers, websites, organisations, reference lists and other sources searched or consulted to identify studies. Specify the date when each source was last searched or consulted.	Page 5<br>Sup Table 2
Search strategy	7	Present the full search strategies for all databases, registers and websites, including any filters and limits used.	Sup Table 2
Selection process	8	Specify the methods used to decide whether a study met the inclusion criteria of the review, including how many reviewers screened each record and each report retrieved, whether they worked independently, and if applicable, details of automation tools used in the process.	Pages 5-6
Data collection process	9	Specify the methods used to collect data from reports, including how many reviewers collected data from each report, whether they worked independently, any processes for obtaining or confirming data from study investigators, and if applicable, details of automation tools used in the process.	Page 6
Data items	10a	List and define all outcomes for which data were sought. Specify whether all results that were compatible with each outcome domain in each study were sought (e.g. for all measures, time points, analyses), and if not, the methods used to decide which results to collect.	Page 7
	10b	List and define all other variables for which data were sought (e.g. participant and intervention characteristics, funding sources). Describe any assumptions made about any missing or unclear information.	Page 7
Study risk of bias assessment	11	Specify the methods used to assess risk of bias in the included studies, including details of the tool(s) used, how many reviewers assessed each study and whether they worked independently, and if applicable, details of automation tools used in the process.	Page 6
Effect measures	12	Specify for each outcome the effect measure(s) (e.g. risk ratio, mean difference) used in the synthesis or presentation of results.	Supp table 7-8
Synthesis methods	13a	Describe the processes used to decide which studies were eligible for each synthesis (e.g. tabulating the study intervention characteristics and comparing against the planned groups for each synthesis (item #5)).	Page 5
	13b	Describe any methods required to prepare the data for presentation or synthesis, such as handling of missing summary statistics, or data conversions.	Page 6-7

Section and Topic	Item #	Checklist item	Location where item is reported
	13c	Describe any methods used to tabulate or visually display results of individual studies and syntheses.	Page 7-8
	13d	Describe any methods used to synthesize results and provide a rationale for the choice(s). If meta-analysis was performed, describe the model(s), method(s) to identify the presence and extent of statistical heterogeneity, and software package(s) used.	Page 7-8
	13e	Describe any methods used to explore possible causes of heterogeneity among study results (e.g. subgroup analysis, meta-regression).	N/A
	13f	Describe any sensitivity analyses conducted to assess robustness of the synthesized results.	N/A
Reporting bias assessment	14	Describe any methods used to assess risk of bias due to missing results in a synthesis (arising from reporting biases).	N/A
Certainty assessment	15	Describe any methods used to assess certainty (or confidence) in the body of evidence for an outcome.	N/A
**RESULTS**			
Study selection	16a	Describe the results of the search and selection process, from the number of records identified in the search to the number of studies included in the review, ideally using a flow diagram.	Page 8 Figure 1
	16b	Cite studies that might appear to meet the inclusion criteria, but which were excluded, and explain why they were excluded.	
Study characteristics	17	Cite each included study and present its characteristics.	Supp table 7-8
Risk of bias in studies	18	Present assessments of risk of bias for each included study.	Supple table 3-4
Results of individual studies	19	For all outcomes, present, for each study: (a) summary statistics for each group (where appropriate) and (b) an effect estimate and its precision (e.g. confidence/credible interval), ideally using structured tables or plots.	Supp table 7-8
Results of syntheses	20a	For each synthesis, briefly summarise the characteristics and risk of bias among contributing studies.	Page 8-10
	20b	Present results of all statistical syntheses conducted. If meta-analysis was done, present for each the summary estimate and its precision (e.g. confidence/credible interval) and measures of statistical heterogeneity. If comparing groups, describe the direction of the effect.	Pages-11-12
	20c	Present results of all investigations of possible causes of heterogeneity among study results.	
	20d	Present results of all sensitivity analyses conducted to assess the robustness of the synthesized results.	N/A
Reporting biases	21	Present assessments of risk of bias due to missing results (arising from reporting biases) for each synthesis assessed.	N/A
Certainty of evidence	22	Present assessments of certainty (or confidence) in the body of evidence for each outcome assessed.	N/A
**DISCUSSION**			
Discussion	23a	Provide a general interpretation of the results in the context of other evidence.	Page 13
	23b	Discuss any limitations of the evidence included in the review.	Page 13-14
	23c	Discuss any limitations of the review processes used.	Page 15

Section and Topic	Item #	Checklist item	Location where item is reported
	23d	Discuss implications of the results for practice, policy, and future research.	
**OTHER INFORMATION**			
Registration and protocol	24a	Provide registration information for the review, including register name and registration number, or state that the review was not registered.	Page 5
	24b	Indicate where the review protocol can be accessed, or state that a protocol was not prepared.	Page 5
	24c	Describe and explain any amendments to information provided at registration or in the protocol.	n/a
Support	25	Describe sources of financial or non-financial support for the review, and the role of the funders or sponsors in the review.	
Competing interests	26	Declare any competing interests of review authors.	
Availability of data, code and other materials	27	Report which of the following are publicly available and where they can be found: template data collection forms; data extracted from included studies; data used for all analyses; analytic code; any other materials used in the review.	

From: Page MJ, McKenzie JE, Bossuyt PM, Boutron I, Hoffmann TC, Mulrow CD, et al. The PRISMA 2020 statement: an updated guideline for reporting systematic reviews. BMJ 2021;372:n71. doi: 10.1136/bmj.n71

For more information, visit: http://www.prisma-statement.org/

Supplementary Table 2 Full search strategy

Database	Search Terms
Medline (Ovid)	 1. adolescent/<br>2. (teen* or adolescen* or young people or youth).mp. [mp=title, abstract, original title, name of substance word, subject heading word, floating sub-heading word, keyword heading word, organism supplementary concept word, protocol supplementary concept word, rare disease supplementary concept word, unique identifier, synonyms]<br>3. 1 or 2<br>4. Built Environment/<br>5. exp Food Services/<br>6. ("food environment" or "nutrition environment" or "community environment" or "consumer environment" or "store environment").mp. [mp=title, abstract, original title, name of substance word, subject heading word, floating sub-heading word, keyword heading word, organism supplementary concept word, protocol supplementary concept word, rare disease supplementary concept word, unique identifier, synonyms]<br>7. exp Marketing/<br>8. exp Geographic Information Systems/<br>9. "global positioning system".mp. [mp=title, abstract, original title, name of substance word, subject heading word, floating sub-heading word, keyword heading word, organism supplementary concept word, protocol supplementary concept word, rare disease supplementary concept word, unique identifier, synonyms]<br>10. 4 or 5 or 6 or 7 or 8 or 9<br>11. 3 and 10<br>12. exp Food/<br>13. exp Beverage/<br>14. exp diet/ or exp feeding behavior/<br>15. (food or eat* or diet* or drink* or beverage* or "diet* quality").mp. [mp=title, abstract, original title, name of substance word, subject heading word, floating sub-heading word, keyword heading word, organism supplementary concept word, protocol supplementary concept word, rare disease supplementary concept word, unique identifier, synonyms]<br>16. "food choice".mp. [mp=title, abstract, original title, name of substance word, subject heading word, floating sub-heading word, keyword heading word, organism supplementary concept word, protocol supplementary concept word, rare disease supplementary concept word, unique identifier, synonyms]<br>17. 12 or 13 or 14 or 15 or 16<br>18. Consumer Behavior/<br>19. (purchas* or buy* or sale* or shop*).mp. [mp=title, abstract, original title, name of substance word, subject heading word, floating sub-heading word, keyword heading word, organism supplementary concept word, protocol supplementary concept word, rare disease supplementary concept word, unique identifier, synonyms]<br>20. 18 or 19<br>21. 17 or 20

	22. 11 and 21<br>23. limit 22 to (english language and yr=1995-2021)<br><br>**UPDATED ON 3RD January 2023
PsycInfo (EbscoHost)	DE "Adolescent Behavior"<br><br>teen* OR "young people" OR youth OR adolescen*<br><br>S1 OR S2<br><br>DE "Drinking Behavior" OR DE "Eating Behavior" OR DE "Food" OR DE "Fast Food"<br><br>DE "Beverages (Nonalcoholic)" OR DE "Energy Drink"<br><br>food* OR eat* OR diet* OR drink* OR beverage* OR "food choice" OR "diet quality"<br><br>DE "Consumer Behavior" OR DE "Shopping"<br><br>"consumer behavi*"<br><br>purchas* OR buy* OR sale* OR shop*<br><br>S4 OR S5 OR S6 OR S7 OR S8 OR S9<br><br>DE "Built Environment"<br><br>DE "Hospitality Industry"<br><br>"food environment" OR "local food environment" OR "nutrition environment" OR "community environment" OR "consumer environment" OR "store environment"<br><br>DE "Retailing" OR DE "Marketing"<br><br>S11 OR S12 OR S13 OR S14<br><br>S3 AND S10 AND S15<br><br>**Limiters** - Publication Year: 1995-2021<br><br>**UPDATED ON 3RD January 2023
CINAHL(EbscoHost)	(MH "Adolescence")<br><br>teen* OR "young people" OR youth OR adolescen*<br><br>S1 OR S2<br><br>(MH "Eating Behavior+")<br><br>(MH "Drinking Behavior+")<br><br>(MH "Food and Beverages+")<br><br>food* OR eat* OR diet* OR drink* OR beverage* OR "food choice" OR "diet quality"

	purchas* OR shop* OR buy* OR sales OR "food purchasing"<br><br>S4 OR S5 OR S6 OR S7 OR S8<br><br>(MH "Built Environment")<br><br>(MH "Food Services+")<br><br>"food environment" OR "local food environment" OR "nutrition environment" OR "community environment" OR "consumer environment" OR "store environment"<br><br>S10 OR S11 OR S12<br><br>S3 AND S9 AND S13<br><br>Published Date: 19950101-20210831<br><br>**UPDATED ON 3RD January 2023
Econlit (EbscoHost)	teen* OR "young people" OR youth OR adolescen*<br><br>food* OR eat* OR diet* OR drink* OR beverage* OR nutri* OR "diet quality" OR "Food choice"<br><br>"consumer behavi*" OR purchas* OR buy* OR sale* OR shop* OR "food purchas*"<br><br>"food environment" OR "local food environment" OR "nutrition environment" OR "community environment" OR "consumer environment" OR "store environment"<br><br>S2 OR S3<br><br>S1 AND S4 AND S5<br><br>**UPDATED ON 3RD January 2023
Scopus<br><br>(search not saved on database- string entered as one item in advanced search)	(TITLE-ABS-KEY (adolescen* OR adolescen* OR youth OR "young people" OR teen*) AND TITLE-ABS-KEY (food* OR drink* OR beverage* OR diet* OR "diet quality" OR feed* OR "Food Consumption" OR eat* OR purchas* OR buy* OR shop* OR sale* OR "Food purchas*") AND TITLE-ABS-KEY ("food environment" OR "local food environment" OR "nutrition environment" OR "community environment" OR "consumer environment" OR "store environment") AND PUBYEAR > 1994)<br><br>**UPDATED ON 3RD January 2023
GEOBASE<br><br>(search not saved on database- string entered as two separate lines	(adolescen* OR youth OR “young people” OR teen*)<br>AND<br>("food environment" OR "local food environment" OR "nutrition environment" OR "community environment" OR "consumer environment" OR "store environment")

Supplementary Table 3 Risk of Bias Assessment Criteria for Observational Studies

This tool has been made specifically for this systematic review. The criteria assessed are based on the advice provided by the CRD guidance and the NIH Quality Assessment Tool for Observational Cohort and Cross-Sectional Studies https://www.nhlbi.nih.gov/health-topics/study-quality-assessment-tools

Criteria	Low<br>Risk of bias<br>(1)	Moderate<br>Risk of bias<br>(0)	High<br>Risk of bias<br>(-1)
**Was the study design appropriate to address the research question?**<br>*1)* Participants use of store-reported or assumed? (Consumer)<br>Activity spaces based on assumed food outlet exposure or actual exposure e.g. GPS (community)<br>*2)* Is the study longitudinal?<br>*3)* How was recruitment carried out? Convenience sample (-1), purposively sampling (0), random sample (1)	Study design appropriate for the outcome and setting chosen to minimise bias. Rigorous study design for all 3 of the criteria listed	Study design not obviously inappropriate; setting is possibly restricting but reflected in the scope of the objectives and the conclusions drawn. Rigorous study design for 2 criteria listed	Study design is ambiguously described **OR** obviously bias inducing or unsuitable for the objectives and stated conclusions.<br>Rigorous study design for <2 criteria listed
**Was the study population clearly defined?**<br>• If you were to repeat this study would you know who to choose?<br>• Age/geographic location/SES/sex	Inclusion/exclusion criteria for study participants is clearly defined using 3 or more useful descriptors **AND** good descriptive variables are provided for participants	Inclusion/exclusion criteria for study participants is clearly defined using 3 or more useful descriptors **OR** good descriptive variables are provided for participants	Little or no information given
**Was the participation rate of eligible persons at least 50%?**	Yes, over 50% of the eligible population participated	N/A	No, less than 50% of the eligible population participated **OR**<br>No details provided about participation rate
**Was the sample size appropriate?**<br>• Number of participants in final sample used for analysis	Large study size (over 1000) **OR** sample size calculation provided and met	Average study size (100-1000) when no sample size calculation is present	Extremely ambiguous, not given, or small study size (under 100)
**What proportion of the cohort was followed-up?**	% FU is high (over 90%)	% FU is low to average (70-90%) **OR** cross sectional study	% FU is not given, unclear, **OR** very low (below 70%)
**Adequate information on non-participants?**<br>• Cross-sectional- Did only a % of the cohort participate? If so, were these people different to those that did participate?<br>• Or, is the study sample representative of the wider population?	Information on drop outs is given completely and has little or no potential for bias **OR** cross-sectional with good comparison with general population (at least 3 variables)	Some information is given on drop-outs and no potential for bias **OR** complete information is given but suggests a medium potential for bias **OR** cross sectional with comparison with general population (using only 1 or 2 variables)	No information provided **OR** whatever information is given suggests a high potential for bias
**Was the exposure measurement of community or consumer**	Detailed description of the exposure **AND (one of the following)** reliability/validity	Detailed description of exposure provided **AND** an appropriate data collection	Inadequately explained **OR** obviously unsuitable data collection method

**environment is appropriate?**<br><br>• Has the tool been validated in this population?<br>• validation in same age group published or validation data provided in the manuscript<br>• Have outlet types been defined? (classification codes or other method?)	of the tool that is used to collect the data is reported **OR** there has been an objective measure of the exposure e.g. ground trothing to verify presence of stores	tool has been used or a reliability measure has not been conducted	
**Were the measurements of dietary outcomes/purchasing data reliably ascertained?**<br>• F&V intake<br>• Consumption/purchase of high fat/ high sugar foods?<br>• Consumption/purchase of healthier food options<br>• Validated methods used<br>• Modification of tools	The study reports a clear description of the diet/purchasing outcome(s) **AND** self-reported data collection instruments have been validated **OR** objective measure of purchasing have been used (includes receipt collection/bag checks)	Adequate description of the study outcomes **AND** the outcome measures appear suitable (validated in another group)<br><br>Or some measure of reliability has been reported?	Inadequately explained **OR** obviously unsuitable. Unreliable methods including questionnaires that have not been piloted or validated, and methods that increase the likelihood of socially acceptable responses
**Were point estimates and measures of variability reported for primary outcome measures?**	All dietary/purchasing outcomes are reported with measures of variability such as SD, SE, or CI	Some variability measures are reported	No variability measures are reported
**Were the analytical methods appropriate?**<br>Clustering needs to be considered when students from multiple school were included in study. These students are more likely like each other than they are like students from another school	Statistical tests used are rigorous, and appropriate for the data set. Regression (or similar technique) used which gives a valid measure of association (e.g. odds ratios, hazard ratios or relative risk)	Tables of means & differences given with statistical tests (e.g. t-tests) **OR** some regression but without clear/valid measure of association	Statistical methods are limited and only report descriptive stats **OR** tests used are not appropriate for the dataset
**Did the analysis adjust for important confounding factors?**<br>• Educational attainment/SES<br>• BMI/weight<br>• Gender<br>• Age	The statistical model used adjusted for most of the important confounding factors. Justifications are provided for why some may not be included as adjustments	Most controlled for in tables or fewer if one or more is adjusted for in regression	Important adjustment were not made (whether they were measured or not is irrelevant)
**Funding and Conflict of Interests (CoIs)**	CoIs declaration (none) **AND** non-industry funded study	Details about 1 aspect only provided. CoIs declaration (none) **OR** non-industry funded study	Conflicting funding **AND/OR** conflicts of interest are present

Supplementary Table 4 Risk of Bias Assessment Criteria for Intervention Studies

This tool has been made specifically for this systematic review. The criteria assessed are based on the advice provided by the CRD guidance and the NIH Quality Assessment Tool for Controlled Intervention Studies https://www.nhlbi.nih.gov/health-topics/study-quality-assessment-tools

Criteria	Low Risk of Bias (+1)	Medium Risk of Bias (0)	High Risk of bias (-1)
**Was the study design appropriate?**	Randomised controlled trial	Quasi-experimental studies that include a control group	Experimental studies that do not use a control group
**Were the study inclusion/exclusion criteria clearly defined?**<br>• If you were to repeat this study would you know who to choose?<br>• Age/geographic location/SES/sex	Inclusion criteria are very clear, at least 3 criteria defined	Inclusion criteria reported, but not very specific	Selection criteria are not reported
**What was the approach participant recruitment?**	Participants were recruited from multiple schools/ youth centers attempting to provided representative sample of participants **OR** participants were recruited (in store) over a variety (at least 3) of days and time points **OR** two recruitment strategies were used	Some attempt has been made to ensure recruitment of participants is representative	No details provided **OR** recruitment only conducted at one-time point in one location
**Was the sample size appropriate?**	Power calculation is reported **AND** sample size meets the requirements of the power calculation	Power calculation is not reported, but text states that recruitment was based on a power calculation **OR** sample size is very large (>1000)	No power calculation, unclear whether sample size is adequate O**R** powered for different measure.
**Was assignment to treatment groups truly random?**	Random numbers table **OR** computer randomisation	Randomised by methods other than random number table **OR** randomised in large clusters.	Not randomised **OR** inadequate randomisation methods such as birth date used **OR** randomisation methods not described
**Were groups similar at baseline?**	Appropriate statistical tests (chi-square and/or t-tests) used to analyse differences between groups at baseline, and found that there were no significant differences	Groups are similar, but there are some differences that are explained and judged to be acceptable	No test for differences at baseline, or there were significant differences between groups **OR** No control group
**Were outcome assessors blinded to the intervention allocation?**	Assessors (statisticians, anyone taking measurements, and the participants in self-reported questionnaires) are unaware of treatment allocation	N/A	At least one assessor is aware of the treatment allocation **OR** blinding not discussed
**Were participants blinded to the intervention allocation?**	A good control was used so that participants were not	It was not possible to blind the participant to the intervention	No control used

	aware whether they were receiving the intervention.		
**What proportion of participants were lost to follow-up?**	Loss to follow up of less than 10% **AND** similar loss in all groups	More drop outs than expected (10%-30%). Similar loss between groups.	High dropout rate (>30%) **AND/OR** large difference in follow up between groups.
**Were there differences between completers and non-completers?**	Statistical tests used **AND** no clear differences between groups **OR** no drop outs	Differences generally reported **OR** slight differences between groups with explanation	Not reported
**What are the reasons for drop outs?**	Reasons for drop out are clearly reported, and specific to participants and/or treatment group **OR** no drop outs.	Reasons generally reported	Reasons not reported
**Was the intervention executed in the same way across all participants/ clusters?**	Good description of intervention and how it was implement. The intervention was delivered in a way that was not variable	The intervention was probably delivered consistently for all participants (did not change over time)	The intervention was likely have been delivered differently across the trial. Intervention was based on the needs of each individual store.
**Were dietary outcomes/purchase data reliably ascertained?**	The study reports a clear description of the diet/purchasing outcome(s) **AND** self-reported data collection instruments have been validated **OR** objective measure of purchasing have been used (includes receipt collection/bag checks)	Adequate description of the study outcomes **AND** the outcome measures appear suitable (validated in another group)	Inadequately explained **OR** obviously unsuitable. Unreliable methods including questionnaires that have not been piloted or validated, and methods that increase the likelihood of socially acceptable responses
**Were point estimates and measures of variability reported for primary outcome measures?**	All dietary outcomes are reported with measures of variability such as SD, SE, or CI	Some variability measures are reported	No variability measures are reported
**Were the analytical methods appropriate?**	Statistical tests used are rigorous, and appropriate for the data set. (Regression modelling, ANOVA, etc)	Statistical methods are effective, but not as rigorous as they could be.	Statistical methods are limited, and only report descriptive stats **OR** tests used are not appropriate for the dataset.
**Did the analysis adjust for confounding?**<br>• Educational attainment or SES<br>• BMI/weight<br>• Gender<br>• Age	The statistical model used adjusts for all relevant confounding factors	The model adjusts for most confounders	The analysis adjusts for very few or no confounders
**Funding and Conflict of Interests**	CoIs declaration (none) **AND** non-industry funded study	Details about 1 aspect only provided. CoIs declaration (none) **OR** non-industry funded study	Conflicting funding **AND/OR** conflicts of interest are present

Supplementary Table 5 Risk of bias ratings for observational studies

Author, year	Study Design	Population	Participation Rate	Sample Size	Follow-up	Non participants	Exposure	Outcome	Variability	Statistical Methods	Confounders	CoI and funding	Overall risk of bias rating
An, 2012	-1	0	-1	1	0	-1	0	-1	1	0	1	1	Moderate
Berge, 2014	-1	0	-1	1	0	-1	0	0	1	1	1	1	Moderate
Clark, 2014	-1	1	1	0	0	1	0	1	1	1	1	0	Low
Cutumisu, 2016	-1	0	-1	1	0	1	0	-1	1	1	1	1	Moderate
Davis, 2009	-1	0	-1	1	0	-1	-1	-1	1	1	1	0	High
Edmond, 2001	-1	0	-1	-1	0	-1	-1	-1	0	-1	-1	0	High
Forsyth, 2012	-1	0	1	1	0	-1	1	0	-1	-1	1	-1	High
Godin, 2018	-1	0	1	1	0	-1	0	0	1	0	-1	1	Moderate
Grier and Davis, 2013	-1	1	-1	1	0	-1	0	-1	1	0	-1	-1	High
Gustafson, 2017	-1	1	-1	0	0	-1	0	0	1	0	0	1	Moderate
Hager, 2017	-1	1	1	0	0	1	0	1	1	0	1	1	Low
He, 2012	-1	0	-1	0	0	-1	1	0	1	1	1	1	Moderate
He, 2012	-1	1	-1	0	0	-1	1	0	1	1	1	1	Moderate
Jago, 2007	-1	0	-1	0	0	0	0	0	1	1	1	1	Moderate
Kelly, 2019	0	0	-1	1	0	-1	0	1	1	0	1	1	Moderate
Khan, 2012	-1	0	-1	1	-1	-1	-1	-1	1	1	1	0	High
Laska, 2010	-1	1	-1	0	0	0	0	0	1	1	1	1	Moderate
Laxer, 2013	-1	1	-1	1	0	-1	0	0	1	1	1	0	Moderate
Loh, 2022	-1	0	-1	0	0	-1	0	-1	1	1	1	1	Moderate
Longacre, 2012	-1	0	1	1	0	-1	0	-1	1	1	1	0	Moderate
Powell, 2011	-1	0	-1	1	0	-1	0	0	1	1	1	0	Moderate
Sadler, 2016	-1	-1	-1	0	0	-1	0	-1	1	1	0	1	High
Seliske, 2013	-1	1	-1	1	0	-1	0	-1	1	1	1	1	Moderate
Shareck, 2017	-1	1	-1	1	0	-1	0	0	1	1	1	1	Moderate
Shearer, 2015	0	-1	-1	0	0	-1	0	0	-1	0	0	0	Moderate
Shier, 2016	-1	1	1	0	0	-1	0	0	1	1	1	-1	Moderate
Smith, 2013	0	1	-1	0	-1	-1	0	-1	1	1	1	1	Moderate
Sturm, 2011	-1	0	-1	1	0	-1	0	0	1	1	1	1	Moderate
Svastusalee, 2012	-1	0	1	1	0	-1	1	1	1	1	1	1	Moderate
Svastisalee, 2015	-1	0	1	1	0	-1	1	0	1	1	0	1	Moderate
Timperio, 2018	0	1	-1	0	-1	-1	0	0	1	1	1	1	Moderate
Trapp, 2021	-1	0	-1	1	0	-1	0	-1	1	1	1	1	Moderate
van der Horst, 2008	-1	0	-1	1	0	-1	0	-1	-1	1	1	1	High
Virtanen, 2015	-1	0	1	1	0	-1	-1	-1	1	1	1	1	Moderate

Supplementary Table 6 Risk of bias ratings for intervention studies

Author, year	Study Design	Population	Participation Rate	Sample Size	Randomisation	Group differences	Assessor Bliding	Participant blinding	Follow-up	Completers	Drop outs	Intervention delivery	Outcomes	Variability	Statistical Methods	Confounders	CoI and funding	Overall risk of bias rating
Lawman, 2015	-1	1	-1	-1	-1	-1	-1	-1	-1	-1	-1	-1	0	-1	0	-1	0	High
Shin, 2015	1	1	0	-1	1	0	-1	0	-1	1	-1	-1	-1	-1	-1	-1	1	High

Supplementary Table 7: Details of included observational studies

General Information				Population					Exposure					Outcome				Statistical Methods						
Author, year. Country	Setting	Study Name	Study Design	Sample Size	Age	Sex/ Gender	Socio-economic status	Race/ Ethnicity	Community or Consumer	Exposure Details	Assessment method & Data Source	Food outlet type and definition (if provided)	Exposure Type	Purchasing/ Diet	Outcome details	Assessment method	Outcome Type	Statistical Model	Individual level covariates	Area-level covariates	Clustering level	Mutual Adjustment for multiple food outlets	Sub-group analyses	Food environment interaction
An, 2012.<br><br>USA	Households in California sampled using landline and mobile phone random digit dial sampling frame	California Health Interview Survey (2005 & 2007 waves)	Cross sectional	5236	Mean 14.5 yrs. (SD 1.7)	Boys 51%; Girls 49%	Parent education:<br>< High school 21%;<br>High school 39%;<br>College or above 40%	White 39%;<br>Hispanic 31%;<br>Asian 11%;<br>Black 8%;<br>Other 19%	Community	Number of fast-food outlets, convenience store, small food stores, medium and large supermarkets within 0.5 mile buffer of home and school	GIS circular buffers surrounding home and school using radius of 0.5 miles<br><br>Industry database of business and consumer lists, InfoUSA 2006	FFOs= menus containing items such as hotdogs, burgers, pizza, fried chicken, subs or tacos (NAICS 72221105-6<br><br>CS= NAICS 44512001;<br><br>Small food stores= annual sales <$1 million.<br><br>Midsize grocery stores= annual sales $1-5 million;<br><br>Large supermarkets= ann	Continuous	Diet	Self-reported consumption in previous day of:<br>1) fruit,<br>2) vegetables,<br>3)100% juice,<br>4) soda,<br>5) high-sugar food<br>6) fast-food<br><br>(portion sizes were self-defined)	Administered questionnaire<br><br>No details of validation of dietary questions	Continuous	Negative binomial regression	Gender; age; ethnicity; household size; household income; parents BMI; survey wave	None	N/A	N/A	N/A	n/a
Berge, 2014.<br><br>USA	20 public middle and high schools in Minneapolis/ St.Pauls Metropolitan area of Minnesota	EAT2010	Cross sectional	2682	Mean 14.5 yrs (SD 2.0)	Girls 53%; Boys 47%	Low SES 29%;<br>Low-middle SES 23%;<br>Middle SES 33%;<br>Upper middle SES 6%;<br>High SES 3%	White 19%;<br>Black 29%;<br>Asian 20%;<br>Hispanic 17%;<br>Native American 3%;<br>0ther 12%	Community	1) Presence of fast-food outlets within 1200m from home;<br><br>2) High density of fast-food outlets (5+) within 1600m from home,<br><br>3) Presence of convenience store within 1200m from home;<br><br>4) Presence of supermarket within 2400m from home;<br><br>5) Unsupportive bui	Car accessible road network buffers using ArcGIS (1200m, 1600m or 2400m depending on exposure type).<br><br>Commercial database- ESRI Business AnALYST 2010	FFO=NAICS codes 722110, 722211, 722212, and722213;<br><br>CS=NAICS codes 44512, 44711, and44719;<br><br>Supermarkets= A local chain for supermarkets and super center	Binary (for presence and density variables)<br><br>Continuous (0-4) (neighbourhood summary score)	Diet	1) Mean consumption of fast-food per week<br><br>2) Mean consumption of fruit and vegetables per week	Fruit and vegetable consumption: 149 item Youth Adolescent FFQ;<br><br>Fast-food measured using question about consumption in last month for 5 categories of fast-food<br><br>No validation details but test-retest reliability presented	Continuous	Linear regression	Age; ethnicity; SES	None	Analysis conducted at individual household level (individual household buffers) therefore no clustering considered	Unsupportive built food environment: high density of FF + presence of FF and convenience store within 1200m + no nearby (within 2400m supermarket	Separate analysis for boys vs girls	N/A
Clark, 2014.<br><br>New Zealand	11 secondary schools in Otago region, situated less than 1 hour drive from Dunedin	n/a	Cross sectional	664	Mean 15.8 yrs. (Range 15-18 yrs.)	Boys 56%; Girls 44%	Deprivation Level: 1(least deprived) 42%;<br>2 20%;<br>3 17%;<br>4 15%;<br>5 (most deprived) 6%	NZ European 90%;<br>Pacific 1%;<br>M?ori 9%	Community	1) Density of food outlets within an 800m or 1500m radius around schools;<br><br>2) Distance to nearest food outlet from school	ArcGIS using 800m or 1500m radial buffers around school.<br><br>3 data sources: 1) list of Otago tobacco retailers; 2) Local authority food graded outlet list (Dunedin City Council);<br>3) Manual auditing using local knowledge and internet sources	Convenience store= Dairies/ convenience stores, petrol stations and small supermarket;<br><br>Cafes and restaurants= prepared and presented food and in outlet eating. Waiting required for receiving food;<br><br>Supermarkets= packaged food, no in-outlet eating;<br><br>Take	Binary (high vs low)	Diet	Diet Quality Index. Higher scores reflecting greater adherence to healthy eating guidelines	Self-report FFQ consisting of 72 items used to derived validated New Zealand Diet Quality Index for Adolescents	Continuous (1-100)	Gaussian family generalised estimating equation	Age; ethnicity; BMI	Healthy school environment; neighbourhood deprivation score	Schools used as clustering units	None	Separate analysis for boys vs girls	N/A
Cutumisu, 2016.<br><br>Canada	374 public secondary schools in Quebec	Quebec Health Survey of High School Students	Cross sectional	26655	?13ys 28%;<br>?14 yrs. 72%	Boys 51%; Girls 49%	School deprivation: Less deprived 72%;<br>More deprived 28%	None reported	Community	Number of FFOs within a 750m radius street network distance buffer around school	NEMS (store, restaurant and CS tools) conducted by research assistants in Fall and Spring after questionnaire data collection	Fast-food outlets= mobile food stands, take-out restaurants and quick service restaurants	Binary (Low 0-1, High 2+)	Diet	Junk food consumption at lunchtime (JCL) during the previous school week (Monday-Friday)	Self-report through non-validated administered questionnaire	Binary (Low 0-1, High 2+)	Multi-level logistic regression	Sex; age; family structure status; student perceived health status; parental education	N/A	Schools used as clustering units	N/A	N/A	N/A

General Information				Population					Exposure					Outcome				Statistical Methods						
Author, year. Country	Setting	Study Name	Study Design	Sample Size	Age	Sex/ Gender	Socio-economic status	Race/ Ethnicity	Community or Consumer	Exposure Details	Assessment method & Data Source	Food outlet type and definition (if provided)	Exposure Type	Purchasing/ Diet	Outcome details	Assessment method	Outcome Type	Statistical Model	Individual level covariates	Area-level covariates	Clustering level	Mutual Adjustment for multiple food outlets	Sub-group analyses	Food environment interaction
Davis, 2009.<br><br>USA	Middle and high schools across the state of California	Californian Healthy Kids Survey	Cross sectional	529367	<=12yrs 21%; 13 yrs. 11%; 14yrs 24%; 15yrs 12%; 16yrs 23%; 17+ yrs. 9%	Boys 47%; Girl 53%	None reported	White 31%; Asian 10%; Black 4%; Hawaiian 2%; Hispanic 31%; Multiple 14%; Other 8%	Community	Presence of at least one fast-food outlet within half a mile of school	0.5 mile radius around school<br><br>Commercial Database- Microsoft streets & Technomic	Fast-food outlet: defined as top limited service restaurants on Technomic	Binary (Yes/No)	Diet	1) Consumption in the last 24hrs of: vegetables; fruit; juice; soda; fried potato<br><br>2) Servings in the last 24hrs of: vegetables; fruit; juice; soda;	Self-report questionnaire<br><br>Not validation details	Binary for consumption (Yes/No)<br><br>Continuous for servings data	Logit Models<br><br>Negative binomial models	Sex; school grade; age; ethnicity; physical exercise	School location type (urban, suburban, rural)	Schools used as clustering units	N/A	N/A	N/A
Edmond, 2001.<br><br>USA	Boy scout-groups with at least 80% African American members, in Houston area, Texas	N/A	Cross sectional	90 (analysis conducted at the area level n=11)	11-14 yrs.	Boys 100%	None reported	Black 100%	Consumer	Mean availability of fruit, vegetables and 100% fruit juice in census tract	Store audits- checklist of availability (Yes/No) and shelf space measure using measuring tape	Supermarkets, convenience stores, petrol station, and restaurants	Continuous	Diet	Mean consumption of 1) fruit; 2) vegetables; 3)100% fruit juice for participants living in census tract	Interviewer-led 24 hour diet recalls collected at two time-points, one week apart	Continuous	Pearson's correlation	N/A	Median family income for census tract	N/A	N/A	N/A	N/A
Forsyth, 2012.<br><br>USA	20 public middle and high schools in Minneapolis/St. Pauls	EAT2010	Cross sectional	2724	Mean 14.5 yrs. (SD 2.0)	Girls 53%	Low SES 39%; Low-middle 21%; Middle 17%; Upper middle 12%; High 7%	White 19%; Black 29%; Asian 20%; Hispanic 17%; Other 15%	Community	Number of fast-food outlets within 1) 1600m buffer from home; 2) 800m buffer from school	GIS street network buffer with 150m on either side of road<br><br>Commercial business registries were used- ESRI Business Analyst and the North American Industrial Classification System codes	FFO= NAICS codes 722110, 722211, 722212 and 722213	Continuous	Diet	Frequency of eating from five categories of fast-food outlets: burgers and fries, fried chicken, Mexican, pizza and sandwich restaurant	Administered questionnaire<br><br>No validation details	Continuous	Multivariate analysis<br><br>No further details provided	Gender; school grade; ethnicity; SES	N/A	N/A	N/A	Boys vs girls	N/A
Godin, 2018.<br><br>Canada	Secondary schools in 2 provinces of Canada, Alberta and Ontario	COMPASS study: a prospective cohort study	Cross sectional	41829	Grades 9-12 (14-17 yrs.)	Girls 50%	Mean neighbourhood annual income: Low 8%; Medium 70%; High 23%	White 75%; Aboriginal 4%; Asian 5%, Black 4%; Latin 2%; Other 11%	Community	Presence of 1+ restaurant/ variety store/ food store within 1km circular buffer around school	1km circular buffer around school<br><br>Desktop Mapping Technology Inc.- private location mapping service	Restaurants= fast-food outlets and sit-down restaurants<br><br>Variety Stores= establishments which sell low cost food and non-food items<br><br>Food stores= supermarkets and specialised food stores	Binary (Yes/No)	Diet	Consumption of SSB during a usual school week (Mon-Fri)<br><br>Composite score which includes consumption of soft drinks, sweetened coffee and tea and energy drinks	Self-report questionnaires<br><br>No validation details	Continuous (Range 0-15)	Hierarchical Poisson regression models	None adjusted for in results recorded	None adjusted for in results recorded	Schools used as clustering units	N/A	N/A	N/A
Grier and Davis, 2013.<br><br>USA	Middle and high schools in California	Healthy Kids Survey. State-wide survey required by law for grades 7,9 and 11	Cross sectional	100,000	<=12 yrs. 21%; 13 yrs. 11% ; 14 yrs. 24%; 15 yrs. 12%; 16 yrs. 23% ; 17 yrs.+ 9%	Girls 53%	34% eligible for free school meals	White 40%; Asian 13%; Black 7%; Hispanic 38%; Other 18%	Community	Distance from school to nearest fast-food outlet	Euclidean distance<br><br>Microsoft Streets and Trips. Latitude and longitude coordinate data from 2003	Fast-food outlets as classified by Technomic Inc (a food industry consulting firm)	Continuous	Diet	Consumption of SSB in previous day	Self-report using the Healthy Kids Survey questionnaire 2003-2005<br><br>No validation details	Binary (Yes/No)	Hierarchical Logit Model	N/A	N/A	Not clear details about clustering type and structure of data	N/A	N/A	N/A
Gustafson, 2017.<br><br>USA	Four counties in rural eastern Kentucky and four counties in rural eastern North Carolina	Go Big and Bring it Home Program. Baseline data	Cross sectional	432	Mean 15 yrs.<br><br>Range 14-16 yrs.	Girls 41%	Not reported	White 62%; Black 26%; Other 12%	Consumer	Combined NEMS scores assessing the quality, availability and price of healthy foods in the 3 most frequently visited food outlet. Higher scores=healthier environments<br><br>NEMS (store, restaurant and CS tools) conducted by research assistants in Fall and Spri	NEMS (store, restaurant and CS tools) conducted by research assistants in Fall and Spring after questionnaire data collection	Restaurants; supermarkets; convenience stores used most frequently by participants	Continuous	Diet	Daily intake of fruit and vegetables (cups)<br><br>Daily intake of added sugars (tsp)<br><br>Daily intake of SSBs (tsp)	Self-report using the NHANES 2009-2010 questions for fruits, vegetables, added sugars (derived from foods such as pizza, tomato	Continuous	Linear regression models	Ethnicity; age; sex	N/A	N/A	N/A	N/A	N/A

General Information				Population					Exposure					Outcome				Statistical Methods						
Author, year. Country	Setting	Study Name	Study Design	Sample Size	Age	Sex/ Gender	Socio-economic status	Race/ Ethnicity	Community or Consumer	Exposure Details	Assessment method & Data Source	Food outlet type and definition (if provided)	Exposure Type	Purchasing/ Diet	Outcome details	Assessment method	Outcome Type	Statistical Model	Individual level covariates	Area-level covariates	Clustering level	Mutual Adjustment for multiple food outlets	Sub-group analyses	Food environment interaction
Hager, 2017.<br><br>USA	Secondary schools in large urban area in Baltimore City, Maryland	N/A	Cross sectional	634	Mean 12.1 yrs. (SD 0.7)<br><br>Range 10.1-14.7 yrs.	Girls 100%	Neighbourhood SES indicators:<br><br>1) Median household income $35298<br><br>2) 14% unemployed<br><br>3) 18% family poverty	Black 90%	Community	Home located in food desert: no supermarket (or healthy supermarket alternatives) within 0.4km of home combined with low-income SES factors<br><br>Food swamp:>4 CS and corner shops within 0.4km of home	0.40km Euclidean distance buffer<br><br>Baltimore City Food Environment Map	Supermarkets; convenience stores= smaller stores which typically stock snacks, sodas and candy and have limited amounts of fresh or frozen foods	Binary (Yes/No)	Diet	Consumption of snacks and desserts (servings per day)	Self-report using the validated Youth/Adolescent FFQ (YAQ)	Continuous	Bivariate ANOVA models<br><br>Multivariate linear regression	Age; BMI	Neighbourhood SES (for food swamp analysis only)	Not accounted for as all schools were part of the same school system and served the same menu of food daily	Living in both a food desert and food swamp was considered	N/A	N/A
He, 2012 (a).<br><br>Canada	Purposive heterogeneous sample of elementary schools in London, Ontario	N/A	Cross sectional	782	Range 11-13 yrs. (grades 7-8)	Girls 51%	Father's education:<br>High school 34%<br>University 57%<br>Graduate school 10%	Not reported	Community	Junk food density: number of fast-food outlets and convenience stores within 1km buffer of home and school<br><br>Junk food proximity: shortest distance from home and school to nearest fast-food outlet and convenience store	GIS 1km straight line buffer from central point of home/school postcode<br><br>Local business directories validated through use of telephone calls, field surveys and inspections of aerial photographs and geocoded to the building address	Fast-food outlets=restaurants were ready to eat foods were ordered at the counter and paid in advance<br><br>Convenience stores= classified as small variety stores	Density: Categorical (none, 1-2, 3+)<br><br>Proximity: Binary (<=1km or >1km)	Purchasing	1) Fast-food purchasing when alone or with friends<br><br>2) Fast-food purchasing with parents<br><br>3) Convenience store purchasing when alone or with friends<br><br>4) Convenience store purchasing with parents	Non-validated self-report questionnaire	Binary (<1 time per week; 1+ times per week)	Logistic regression models<br><br>Hierarchical non-linear regression models	Gender; grade; father's education level	N/A	Schools used as clustering units (only conducted for analyses with 1km school buffer	N/A	N/A	N/A
He, 2012 (b).<br><br>Canada	Purposive heterogeneous sample of elementary schools in London, Ontario	N/A	Cross sectional	580	11yrs 1%;<br>12yrs 34%;<br>13yrs 49%;<br>14 yrs. 16%	Girls 52%	Family income:<br>Low 9%; Medium 22%;<br>High 32%;<br>Missing 37%	White 75%;<br>Latino 7%;<br>Asians 5%;<br>Other 14%	Community	Proximity: distance from home/school to nearest convenience store, fast-food outlet and supermarket<br><br>Density: Number of fast-food outlets in 1km buffer from home/school	Distance: closest distance using road/path network<br>Buffer: 1km Euclidean distance<br><br>Local business directories validated through use of telephone calls, field surveys and inspections of aerial photographs and geocoded to the building address	Fast food outlets; Convenience Stores; Supermarkets	Binary	Diet	Diet Quality assessed using modified HEI-2005 score. Score range 0-80	Dietary data collected using self-administered validated Block Kids 2004 FFQ	Continuous	Generalised linear mixed models	School grade; gender	Neighbourhood distress score for census area base on prevalence of low educational attainment, lone parenthood, unemployment and incidence of low income	Schools used as clustering units	N/A	N/A	N/A
Jag, 2007.<br><br>USA	38 Boy Scout troops within the greater Houston area, Texas	N/A	Cross sectional	204	Mean 12.8 yrs.<br>(SD 1.1)	Boys 100%	Parental education:<br>High school 7%;<br>Tech College 21%; College 34%;<br>Postgraduate 38%	White 70%;<br>Other 30%	Community	Distance from participants home to nearest fast-food outlet and small food stores.	Euclidean distance using GIS<br><br>City council public health records. Food outlets identified using NAICS 2002 codes	FFO= NAICS code 722211<br><br>Small food stores= CS (445120) and drug stores (446110)<br><br>[Other food outlets types were considered but results are not presented]	Continuous	Diet	Consumption of 1) fruit and fruit juice<br>2) low fat vegetables<br>3) high fat vegetables (French fries, coleslaw, potato salad)	Validated Cullen FFQ which assesses consumption of 4 juices, 17 fruits and 17 vegetables	Continuous	Linear regression models	BMI percentile; age; ethnicity; parental education; self-efficacy; home availability	N/A	Clustering at Boy Scout troop level	N/A	N/A	N/A

General Information				Population					Exposure					Outcome				Statistical Methods						
**Author, year. Country**	**Setting**	**Study Name**	**Study Design**	**Sample Size**	**Age**	**Sex/ Gender**	**Socio-economic status**	**Race/ Ethnicity**	**Community or Consumer**	**Exposure Details**	**Assessment method & Data Source**	**Food outlet type and definition (if provided)**	**Exposure Type**	**Purchasing/ Diet**	**Outcome details**	**Assessment method**	**Outcome Type**	**Statistical Model**	**Individual level covariates**	**Area-level covariates**	**Clustering level**	**Mutual Adjustment for multiple food outlets**	**Sub-group analyses**	**Food environment interaction**
Kelly, 2019.<br><br>Ireland	63 randomly selected secondary schools	HBSC Ireland	Cross sectional	5344	<14 yrs. 25%; 14 yrs. 24%; 15yrs 21%; 16+yrs 30%	Girls 44%	Social Class: Low 14%; Middle 40%; High 46%	Not reported	Community	Fast-food outlet density: 10% or more of food outlets in 1km radius of school are fast-food outlets	GIS straight line 1km buffer around schools. Food premises were categorised based on the type of food sold using a combination of websites, Google street view and Golden pages.<br><br>Geodirectory: commercial address database from Irish post system.	FFO<br><br>(other food outlets were considered in the final variable but these are not described	Binary (Yes/No)	Diet	Daily consumption of fruit, vegetables, sweets, SSBs and chips	Self-report validated questions: On how many days a week do you usually eat...<br>(Fruit, vegetables, sweets, SSB and chips). Response options ranged from 'Never' to 'Every day more than once a day'	Binary (Yes/No)	Logistic regression	Gender; age; social class	School level covariates: population density, disadvantaged school, internal external food environment	N/A	Considered in primary outcome	N/A	N/A
Khan, 2012.<br><br>USA	Nationwide<br><br>67% Urban residential areas; 13% suburban areas; 19% rural areas	Early Childhood Longitudinal Study	Longitudinal (Baseline 2004; Follow-up 2007)	11,700	Grade 5 (10-11 yrs.) and grade 8 (13-14yrs)	50% female	Parental Income: <$20,000 11%; >$20,000-<=$75,000 51%; >$75,000 38%	White 67%; Black 9%; Hispanic 15%; Mixed 2%;<br>Other 67%	Consumer	Index of fast food price computed for closest zip code using 3 food items in the American Chambers of Commerce Researchers Association	Index calculated using data for McDonalds Quarter Pounder with cheese, a thin crust regular cheese pizza at Pizza Hut/ Pizza Inn and Fried Chicken at KFC or Church's.<br><br>Dun and Bradstreet business directory.	N/A	Continuous	Diet	Self-report number of days in past week fast food was consumed	Question assessed consumption of a meal or snack from a fast food restaurant such as McDonald's, Pizza Hut, Burger King, Kentucky Fried Chicken, Taco Bell, Wendy's and other similar establishments. No validation details.	Numerical categorical	Individual level random effects model	Age; gender; race; mother's education level; family income; urbanicity	Fast-food outlet density	Clustering accounted for a home zip-code level	N/A	N/A	Considered both price (consumer) and fast food outlet density (community) in the same model
Laska, 2010.<br><br>USA	Minneapolis/St. Paul metropolitan area	Identifying Determinants of Eating and Activity Study (IDEA study	Cross sectional	349 adolescent/ parent pairs	Mean 15.4 yrs. (SD 1.7)<br><br>Range 10.8-17.7 yrs.	Male 49%	Household income: Mean $76790 (SD$18006). Range $31,691-$147,640<br><br>Parents education: College level or above 64%	Not reported	Community	Distance to and density of fast-food outlets, convenience stores, restaurants and supermarkets around the participants' homes and schools	Distance: distance from the participant's home or school to food outlets along a GIS street network<br><br>Density: Total number food outlets in GIS street network buffer distance (800m, 1600m and 3000m) divided by land area<br><br>Dun and Bradstreet business directo	FFO; any restaurants; CS; supermarket; any retail facilities (Identified using NAICS codes-specific codes not provided)	Continuous	Diet	Daily consumption of SSBs	Telephone administered 24hr dietary recalls using multiple pass approach. 2 or 3 days of dietary data collected.	Continuous	Multi-level regression models	Gender; age; parent education	School-level SES; area-level SES	Schools used as clustering units	N/A	N/A	N/A

General Information				Population					Exposure					Outcome				Statistical Methods						
Author, year. Country	Setting	Study Name	Study Design	Sample Size	Age	Sex/ Gender	Socio-economic status	Race/ Ethnicity	Community or Consumer	Exposure Details	Assessment method & Data Source	Food outlet type and definition (if provided)	Exposure Type	Purchasing/ Diet	Outcome details	Assessment method	Outcome Type	Statistical Model	Individual level covariates	Area-level covariates	Clustering level	Mutual Adjustment for multiple food outlets	Sub-group analyses	Food environment interaction
Laxer, 2013.<br><br>Canada	Nationwide study with school students living in urban areas (population of at least 10,000) and within 1km circular distance from school	Canadian Health Behaviour in School-Aged Children Survey (2009-2010)	Cross sectional	6099	Range 11-15 years<br><br>11yrs: 17%<br>12yrs: 24%<br>13yrs: 22%<br>14yrs: 18%<br>15+yrs: 20%	Male 50%	Perceived Individual SES:<br>Low 10%<br>Low-med 34%<br>Med-high 32%<br>High 24%<br><br>Neighbourhood SES (average family income, unemployment and education):<br>Low 10%<br>Low-med 24%<br>Med-high 35%<br>High 32%	White 71%	Community	Density (per km2) of fast-food outlets in 1km circular buffer from school	GIS 1km circular straight line buffer<br><br>Data from online Yellow Pages. Name search for food retailers that composed the top 75% of the top 200 chain food retailers in Canada	Fast-food outlet= The top 75% of top 200 chain food retailers in Canada in 2009	Categorical<br><br>(Low 0.01-0.37 restaurants per km2; Moderate 0.38-0.86 per km2; High 0.87+ per km2)	Diet	Non-excessive consumption of fast food =< 2 times per week<br><br>Excessive fast food consumption= 2+ times per week	Self-report questionnaire<br><br>No validation details	Binary (Excessive/ non-excessive)	Multilevel Logistic regression	Age; gender; race; individual SES	Neighbourhood SES	Schools used as clustering units	N/A	N/A	N/A
Loh, 2022.<br><br>Australia	Secondary schools in SA1 area of Melbourne, Australia	Neighbourhood Activity in Youth (NEArbY)<br><br>Data collected August 2014-December 2015	Cross sectional	410	Mean 15.5 yrs. (SD 1.5)	Female 59%	Mean Index of Relative Socioeconomic Disadvantage score = 995 (SD 101.4)<br><br>Range: 380-1137	Not reported	Community	Neighbourhood typologies surrounding home identified through Latent Class Analysis:<br><br>Typology 1: Limited variety/ low number of food outlets<br><br>Typology 2: Some variety/ low number of food outlets<br><br>Typology 3: High variety/ medium number of outlets<br><br>Typolo	1km street network GIS boundary around home<br><br>Outlet locations taken from company websites for supermarkets and FFO. Other retailers were located using local government food registries or phone directories<br><br>No validation details provided.	1) Supermarkets<br>2)Green grocers<br>3)Butchers, poultry and seafood<br>4)Bakeries<br>5)Convenience stores<br>6)Fast-food outlets and major takeaways<br>7)Cafes and restaurants	Categorical (4 food environment typologies)<br><br>Typology 4 (high variety/high number of outlets used as reference group)	Purchasing	Purchasing snack foods on<br>1) journey to school<br>2) journey home from school	Self-report questionnaire using 2 questions:<br>1) how often students bought snack foods to eat on the way to school<br>2) how often students bought snack foods to eat on the way home from school	Separate binary variables for purchases made on journey to and from school.<br><br>1) Once a week or more<br>2) less than once a week	Multilevel logistic regression models	Age, sex	Neighbourhood disadvantage	School used as clustering units	Taken into consideration with typologies	N/A	N/A
Longacre, 2012<br><br>USA	32 New Hampshire and Vermont communities		Cross sectional	1547	Mean 14.4 yrs. (SD 1.0)<br><br>Range 12-18 yrs.	Female 50%	Household income:<br>Low 16%;<br>Medium 40%;<br>High 43%;<br>Missing 2%	White 94%;<br>Other 6%	Community	Number of fast-food outlets in participants town	Two-step process:<br>1) Inventory of in-town fast-food outlets using Google Earth and Yahoo! Yellow Pages<br><br>2) Ground truthing of in-town street networks to verify presence and location of fast-food outlets<br><br>No details of how 'in-town' was defined<br><br>Google	Fast-food outlets= chains with 4 or more restaurants	Categorical<br>(None; 1-4; 5)	Diet	Consumption of fast-food in the previous 7 days (Yes/No)	Self-report questionnaire asking the number of times FF was consumed in the previous 7 days. Example outlets provided- McDonalds, Burger King, KFC, or Dunkin' Donuts.	Binary (Yes/No)	Poisson regression	Age; gender; race; possession of a driver's licence; household income; single-parent status; motor vehicle access	Distance from town centre to nearest urban area; town median household income	Schools used as clustering units	N/A	N/A	N/A

General Information				Population					Exposure					Outcome				Statistical Methods						
**Author, year. Country**	**Setting**	**Study Name**	**Study Design**	**Sample Size**	**Age**	**Sex/ Gender**	**Socio-economic status**	**Race/ Ethnicity**	**Community or Consumer**	**Exposure Details**	**Assessment method & Data Source**	**Food outlet type and definition (if provided)**	**Exposure Type**	**Purchasing/ Diet**	**Outcome details**	**Assessment method**	**Outcome Type**	**Statistical Model**	**Individual level covariates**	**Area-level covariates**	**Clustering level**	**Mutual Adjustment for multiple food outlets**	**Sub-group analyses**	**Food environment interaction**
Powell, 2011<br><br>USA	Nationwide	Child Development Supplement of the Panel Study of Income Dynamics	Cross sectional	1134	Mean 14.8 yrs. (SD 1.9)	Male 51%	Mother's education:<br><br>< high school 14%;<br>college 27%;<br>>college 22%;<br>Missing 8%	White 63%;<br>Black 17%;<br>Hispanic 13%;<br>Other 6%	Community and Consumer	Availability of food outlets (Fast-food outlets, full-service restaurants, supermarkets and convenience stores per 10,000 capita per 10 square miles<br><br>Index of fast food price computed for closest zip code using 3 food items in the American Chambers of Co	Availability calculated per 10,000 capita per 10 square miles using Census 2000 zip code level population and land area estimates<br><br>Price index calculated using data for McDonalds Quarter Pounder with cheese, a thin crust regular cheese pizza at Pizza Hut	FFO= standard industrial classification code level for categories of FFO, stands, chain and independent pizzerias but not coffee or ice cream, shops<br><br>Full service restaurants= eating places excluding FFO, coffee & ice cream shops, soft drink and soda foun	Continuous	Diet	Number of days, during previous 7 days, when consumption of 7 food groups occurred:<br>1) fruit and fruit juice;<br>2) vegetables;<br>3) meat;<br>4) non meat protein;<br>5) dairy;<br>6) grains;<br>7) sweets or desserts	Audio computer-assisted self-interview	Continuous	Multivariate regression	Gender; ethnicity; age; martial status of family head; mother's education; mother's work status; family income	Zip code urbanisation level; median household income for zip code	Clustering at zip code level	N/A	N/A	N/A
Sadler, 2016<br><br>Canada	25 elementary schools in south western Ontario	Spatial Temporal Environment and Activity Monitoring (STEAM) Project	Cross sectional (data collected over 2 weeks but analysed in cross sectional manner)	511 (4588 journeys)	Range 9-13 yrs.	Girls 58%	Not reported	Not reported	Community	Exposure to junk food outlets on journeys to and from school defined as the number of minutes the participant was exposed (within 50m) to FFO, variety stores, pizza places or ice-cream shops	GPS data collection of journeys to and from school over 2 week period (1 in Spring and 1 in Autumn). 50m GIS buffer placed around GPS track.<br><br>Analysis conducted at the individual journey level<br><br>Middlesex-London public health inspector's food outlets data	Junk food exposure= FFO, variety stores, pizza places or ice-cream shops. No definitions provided.	Continuous<br>(0-17 mins. Variable truncated at 17mins to remove outliers)	Purchasing	Junk food purchasing during journey to or from school	Daily activity diaries indicating if a junk food purchase had been made on the way to or from school and the location of purchase each day	Binary (Yes/No)	Multilevel logistic regression	N/A	N/A	Clustered within individual adolescents	N/A	N/A	N/A
Seliske, 2013.<br><br>Canada	158 schools from nationwide sample excluding New Brunswick and Prince Edward Island regions	Canadian Health Behaviour in School-Aged Children Survey (HBSC) (2009-2010)	Cross sectional	6971	13yrs 1%;<br>14 yrs. 34%;<br>15 yrs. 47%<br>16 yrs. 19%	Boys 49%	Family Affluence Scale:<br><br>Low 8%;<br>Moderate 36%;<br>High 56%	Not reported	Community	Number of food retailers within 1km of school	2 different methods considered:<br><br>Circular buffer with 1km radius from school<br><br>1km linked road network from school<br><br>The Yellow Pages Online Directory	CS= yellow pages search term 'convenience stores'<br><br>FFO= Top 75% of the top 200 chain food retailers for Canada in 2009 were searched for (name of 16 FFO searched)<br><br>Coffee/donut shops= Top 75% of the top 200 chain food retailers for Canada	Categorical<br><br>(None; 1-2; 3-4; 5+)	Purchasing	Regular purchasing of lunch from snack bar, fast food outlet or café on school days	Self-report questionnaire. Question: "Where do you usually eat your lunch or mid-day meal on school days?" Students who chose the response snack-bar, FFO or café were classified as regularly purchasing their lunch from food retailer<br><br>No validation detai	Binary (Yes/No)	Multilevel Logistic regression	Age; sex; family affluence	School level confounders: cafeterias; vending machines; school snack shops	Schools used as clustering units	N/A	N/A	N/A

General Information				Population					Exposure					Outcome				Statistical Methods						
**Author, year. Country**	**Setting**	**Study Name**	**Study Design**	**Sample Size**	**Age**	**Sex/ Gender**	**Socio-economic status**	**Race/ Ethnicity**	**Community or Consumer**	**Exposure Details**	**Assessment method & Data Source**	**Food outlet type and definition (if provided)**	**Exposure Type**	**Purchasing/ Diet**	**Outcome details**	**Assessment method**	**Outcome Type**	**Statistical Model**	**Individual level covariates**	**Area-level covariates**	**Clustering level**	**Mutual Adjustment for multiple food outlets**	**Sub-group analyses**	**Food environment interaction**
Shareck, 2017.<br><br>UK	25 randomly selected secondary schools in 4 boroughs of East London	Olympic Regeneration in East London (ORiEL) Study	Cross sectional	3089	Mean 14.1 yrs.<br>Range 13-15 yrs.	Girls 43%	Free school meals 33%	White 17%; South Asian 23%; Black 22%; Other 38%	Community	Absolute availability (total number) of fast-food outlets and convenience stores around home and/or school<br><br>Relative availability (proportion of all food outlets) of fast-food outlets and convenience stores around home and/or school	800m pedestrian road network buffer around home and school location<br><br>Local authority registers	FFO= chains and independent outlets<br><br>CS=mini F&V markets, newsagents, tobacconists and confectioners	Continuous	Diet	Weekly frequency of fast-food intake<br><br>Daily sugar sweetened beverage (SSB) consumption	Questionnaire adapted from previous studies<br><br>'How often do you eat takeaways or fast food at home?'<br><br>'How often do you eat takeaways or fast food away from home?'. Examples of typical sources of fast food were given (Pizza Hut, Burger King, Subway, McDon	Binary<br><br>Fast food: <=2-3 d/week and <2-3d/week<br><br>SSB: >=1time/day and <1 time/day	Generalised linear models with Poisson distribution	Age; sex; ethnicity; free school meals	N/A	Not considered as school level clustering was found to be minimal	Considered in relative measure	N/A	N/A
Shearer, 2015.<br><br>Canada	Random sample of 6 schools in Halifax Regional School Board in Nova Scotia stratified by SES and urbanicity	N/A	Cross sectional	315	Range 12-16 years	Not reported	Not reported	Not reported	Community	Average distance to fast-food outlets, restaurants and convenience stores from home and school	GIS 1km road network buffers from home<br><br>GPS based single activity spaces polygons with 50m dissolved buffers<br><br>Distance to each food outlet was calculated. Average calculated for each type of food outlet.<br><br>DMTI Enhanced Points of Interest database of busi	FFO= chains with a focus on take-away meals;<br><br>Restaurants= specific SIC codes not provided;<br><br>Grocery stores= specific SIC codes not provided;<br><br>CS= specific SIC codes not provided	Continuous	Diet	Caloric Intake<br><br>Diet quality<br><br>Fruit and vegetable consumption<br><br>Frequency of fast food consumption<br><br>Frequency of ready-made food consumption	Harvard Youth Assessment Questionnaire- validated food frequency tool.<br><br>Diet quality index derived for each participant	Continuous	Partial Correlations	N/A	Residential SES; Neighbourhood type	N/A	N/A	N/A	N/A
Shier, 2016.<br><br>USA	Purposively selected army installations in all 4 census regions of USA. Selected to represent the majority of army active duty families with eligible children	Military Teenagers Environments Exercise and Nutrition Study (M-Teens)	Cross sectional	941	Mean 13.2 yrs.	Not reported	Household income: <=$50,000 44%	White 41%; Black 21%; Hispanic 25%; Other 14%	Community	Number of fast-food outlets convenience stores, restaurants, small and large supermarkets) within 2 mile radius of home address	2 mile GIS radius from home address. No validation details<br><br>Business directory, InfoUSA 2012	FFO=limited service restaurants (NAICS 722513);<br><br>Restaurants=NAICS 7225511<br><br>CS= NAICS 445120<br><br>Small grocery stores= annual sales <= $2 mil (NAICS 445100)<br><br>Supermarkets=annual sales >$2 mil (NAICS 445100)	Continuous	Diet	Weekly consumption of salty snacks (low-fat or non fat chips; regular chips; other salty snacks)<br><br>Weekly consumption of sweets (candy; doughnuts or other pastries; cookies, brownies, pies and cakes; low fat or non-fat frozen desserts; regular ice cream a	Modified version of the Beverage and Snack Questionnaire which asks about frequency of consumption of fruits, vegetables, soda, and types of salty snacks and sweets among other beverages and foods during the past 7 days	Continuous	Multiple linear regression model	Age; gender; ethnicity; parent education; household income; marital status; number of children in household; army living status; region; importance of proximity to food outlets	N/A	Clustering at army installation level	N/A	N/A	N/A

General Information				Population					Exposure					Outcome				Statistical Methods						
Author, year. Country	Setting	Study Name	Study Design	Sample Size	Age	Sex/ Gender	Socio-economic status	Race/ Ethnicity	Community or Consumer	Exposure Details	Assessment method & Data Source	Food outlet type and definition (if provided)	Exposure Type	Purchasing/ Diet	Outcome details	Assessment method	Outcome Type	Statistical Model	Individual level covariates	Area-level covariates	Clustering level	Mutual Adjustment for multiple food outlets	Sub-group analyses	Food environment interaction
Smith, 2013.<br><br>UK	29 randomly selected secondary schools from 3 boroughs of London with at least 1 food outlets withing 800m of school	Research with East London Adolescents: Community Health Survey (RELACHS)	Longitudinal	524<br><br>*participant details are for follow up	Mean 16.1 yrs. (SD 0.3)	Boys 42%	Free school meal eligibility: 45%	Not reported	Community	Count of total outlets within 400m and 800m road network buffer from school<br><br>Median distance to supermarket or fast-food outlet within 400m and 800m buffer<br><br>Minimum distance to supermarket or fast-food outlet	GIS 400m and 800m road network buffer from school<br><br>Telephone directories. Yellow pages, 2005	Takeaways=FFO. Classified by phone directory listings<br><br>Grocers/supermarkets/CS= Classified by phone directory listings	Continuous	Diet	Healthy diet score<br><br>Unhealthy diet score	Self-report questionnaire<br><br>Healthy diet: Responses to 3 questions: weekly breakfast consumption, daily portions of fruit and veg. Score ranging 0-13<br><br>Unhealthy diet score: Responses to 5 questions about the daily consumption of: crisps and savoury snack	Continuous	Generalised linear model conducted for statistically significant bivariate relationships between measures of the food environment and diet scores	Age; gender; free school meal eligibility; baseline diet score	N/A	Schools used as clustering units	N/A	N/A	N/A
Sturm, 2011.<br><br>USA	Nationally representative cohort of kindergarten children recruited from over 1000 schools in the USA during the 1998–99 school year.<br><br>Data presented from 2004 data collection	Early Childhood Longitudinal Study - Kindergarten Class (ECLS-K)	Cross sectional	4896	Mean 134.3 months (SD 4.6)	Girls 51%	Family income: Low 15%; Medium 46%; High 39%	White 65%; Black 8% Hispanic 18%; Asian 6%	Consumer	Price indices (standardised) based on the mean annual prices for fruit, vegetables, dairy and fast-food at metropolitan area and divided for the Cost of Living index for the metropolitan area.	Price data taken from Cost of Living Index (COLI) database	N/A	Continuous	Diet	Consumption in the previous week of<br>1) fruit and vegetables;<br>2) milk;<br>3) soft drinks;<br>4) fast food	Administered food consumption questionnaire to capture foods eaten in the past week in and out of school<br><br>No validation details	Continuous	Negative binomial regression model	Age; family income; gender mother's education; ethnicity; TV time; physical activity; free school meals; school location; school type; parent-child interaction	N/A	Schools used as clustering units	N/A	N/A	N/A
Svastisalee, 2012.<br><br>Denmark	80 randomly selected Danish secondary schools	Danish Health Behaviour in School-aged Children Study (HBSC)	Cross sectional	6034	Range 11-15 yrs.	Boys 48%	Family Social class:<br>High 23%<br>Middle 41<br>Low 19%<br>Unknown 17%	No details provided	Community	Food outlet concentration: number of food outlets (Supermarkets or fast-food outlets) divided by total road segments within 300m from school	GIS 300m radial buffer around school<br><br>Danish Tax Registry. Ground truthing conducted with good agreement	Supermarkets= All retail, discount, large chain, small independent and grocers<br><br>FFO=FFO and grill bars (own unique NACE code- not specified)	Binary<br><br>(Low/High)<br><br>High exposure to fast food outlets= 2 or more outlets per km road network<br><br>High exposure to supermarkets= 1 or more supermarkets per km road network	Diet	Frequency of fruit and vegetable consumption	Data collected through validated questionnaire	Binary (Infrequent/frequent)<br><br>Infrequent fruit consumption: < once per day<br><br>Infrequent vegetable consumption: < once per day	Multilevel Logistic regression<br><br>All results stratified by family social class.	Age; sex	N/A	Schools used as clustering units	In analysis considering just one food outlet type as an exposure the other type of food outlet (supermarket/ fast food) was considered as a covariate.<br><br>To consider joint exposure to supermarkets and fast-food outlets, the following categories were created	N/A	N/A

General Information				Population					Exposure					Outcome				Statistical Methods						
Author, year. Country	Setting	Study Name	Study Design	Sample Size	Age	Sex/ Gender	Socio-economic status	Race/ Ethnicity	Community or Consumer	Exposure Details	Assessment method & Data Source	Food outlet type and definition (if provided)	Exposure Type	Purchasing/ Diet	Outcome details	Assessment method	Outcome Type	Statistical Model	Individual level covariates	Area-level covariates	Clustering level	Mutual Adjustment for multiple food outlets	Sub-group analyses	Food environment interaction
Svastisalee, 2015.<br><br>Denmark	Randomly selected schools	Health Behaviour in School-aged Children (Denmark)	Cross sectional	4642	Range 11-15 yrs.	Girls 50%	Family Social class:<br>High 33%<br>Middle 36%<br>Low 16%<br>Unknown 17%	Not reported	Community	Number of fast-food outlets within 500m radius of school	GIS 500m radial buffers from school<br><br>Smiley Registry- database maintained by Danish Veterinary and Food Administration	FFO= no definition provided	Categorical<br>(0,1 or 2+ outlets)	Diet	Weekly consumption of fast-food	Self-report non-validated questionnaire: How many times a week do you usually eat fast-food? [Students were prompted with examples of food items such as burgers, sausages, pizza and shawarma (never,<once/week, once/week, 2–4d/week, 5–6 d/week, once/d, >o	Binary (>=1 time per week / <1 time per week	Logistic Multilevel Regression	Sex; school grade; family social class; language; school travel time	School leaving policy; school canteen	Schools used as clustering units	N/A	Boy vs girls	N/A
Timperio, 2018.<br><br>Australia	Randomly selected primary schools located in either the lowest, middle or highest SEP quintile	Health, Eating and Play Study (HEAPS)	Longitudinal (cross-sectional results also presented)	439 (173 in longitudinal analysis)	Baseline<br><br>10-12 years<br>Mean 11.2 yrs. (SD 0.6)	Girls 52%	Highest maternal education:<br>Tertiary 41%;<br>High school or vocational qualification: 39%	No details provided	Community	Neighbourhood typologies surrounding home identified through Latent Class Analysis:<br><br>1) Variety of outlets including staple/fresh food<br><br>2)Cafe/restaurants, fast-food outlets and convenience stores<br><br>3) Very few outlets	Latent class analysis conducted using the data on food outlets present/not present in GIS 800m road network buffer around home.<br><br>Prime safe- compulsory register for butchers, poultry and seafood retailers; Local government food premises register (90% of	Cafes/restaurants= no specific definition;<br><br>FFO= 8 most common chains in Victoria;<br>Bakeries/cake stores= no specific definition;<br><br>Major supermarkets/grocery stores= no specific definition;<br>Greengrocers= no specific definition;<br><br>CS= no specific definiti	Categorical<br><br>1) Variety of outlets including staple/fresh food<br><br>2)Cafe/restaurants/take aways and CS<br><br>3) Very few outlets	Diet	Healthful dietary pattern higher consumption of fruit, dried fruit, vegetables, reduced fat milk and water as well as lower consumption of unhealthy items (Higher scores indicated more healthy diet)<br><br>Energy-dense pattern- higher consumption of energy den	Principal Component Analysis conducted using FFQ data provided by parents on their child's food consumption over the previous week	Continuous	Linear mixed models	Gender; maternal education	Social economic position	Schools used as clustering units<br><br>Within family clustering also accounted for as some participants were from the same family	Considered in main exposure variables	N/A	N/A
Trapp, 2021.<br><br>Australia	17 Perth-metropolitan secondary schools	AMPED UP: An energy drink study	Cross sectional	2389	Range 12-17 yrs.	Girls 56%	Family Affluence Scale:<br><br>Low 21%;<br>Moderate 41%;<br>High 38%	Not reported	Community	Number of food outlets in GIS radial buffers around school at 400m, 800m and 1km distances.<br><br>Food outlets included:<br>1) FFO<br>2) Top 4 FFO chains<br>3) Supermarkets<br>4) Convenience stores<br>5) Other snack/beverage store<br>6) F&V outlet	Food outlet data sourced from Perth local governments	1) FFO= no definition provided<br><br>2) Top 4 FFO chains: 4 of the top FFO chains based on markets research including McDonalds, KFC, Hungry Jacks, Red Rooster.<br><br>3) Supermarkets= no definition provided<br><br>4) Convenience stores= no definition provided<br><br>5) Other	Continuous	Purchasing	Frequency of discretionary food purchasing	Self-report question: how often do you purchase snacks (e.g. soft drinks, energy drinks, cakes/biscuits, chocolate, crisps/chips, hot chips, burgers, sausage rolls, pies) from food outlets near your school?<br><br>Participants could choose from 12 responses ran	Ordinal categorical:<br>-everyday<br>- weekly<br>-monthly or less<br>- never	Mixed-effect ordinal logistic regression	Sex, age, family affluence scale	N/A	School level clustering accounted for	N/A	N/A	N/A

General Information				Population					Exposure					Outcome				Statistical Methods						
Author, year. Country	Setting	Study Name	Study Design	Sample Size	Age	Sex/ Gender	Socio-economic status	Race/ Ethnicity	Community or Consumer	Exposure Details	Assessment method & Data Source	Food outlet type and definition (if provided)	Exposure Type	Purchasing/ Diet	Outcome details	Assessment method	Outcome Type	Statistical Model	Individual level covariates	Area-level covariates	Clustering level	Mutual Adjustment for multiple food outlets	Sub-group analyses	Food environment interaction
van der Horst, 2008.<br><br>The Netherlands	15 secondary schools in Rotterdam, stratified by city region	Environmental Determinants of Obesity in Rotterdam School children (ENDO RSE)	Cross sectional	1174	Mean 14.1yrs (SD1.2)<br><br>Range 12-15 yrs.	Boys 54%	Not reported	Dutch and Western Immigrants 49%	Community	Availability (total number) of fast-food outlets, supermarkets, convenience stores, bakeries and fruit and vegetable stores in 500m straight line buffer from school<br><br>Distance to nearest food store	GIS 500M straight line buffers around school<br><br>Distance calculated using walking street network route<br><br>Business directory, Locatus. No ground truthing conducted	FFO= no definition provided;<br><br>Supermarkets= no definition provided;<br><br>Small food stores= small supermarkets, ethnic food stores, news agencies, stores at petrol stations;<br><br>Bakeries= no definition provided;<br><br>F&V stores= no definition provided;	Categorical<br><br>Convenience stores, bakeries, fruit and vegetable stores based on tertiles (low, medium, high)<br><br>Fast-food outlets based on median (high, low)<br><br>Supermarkets (present vs not)	Diet	Litres of soft drinks consumed per day	Self-report questionnaire using 2 questions:<br>1)How many days a week do you usually drink sugar sweetened beverages?<br>2) If you drink SSB, how many glasses, cans, and/or bottles do you drink on average per day?<br><br>Soft drinks defined as carbonated drinks,	Continuous	Multilevel linear regression	Age; gender; ethnicity; school type (traditional/vocational)	N/A	Clustering accounted for at school and class level	N/A	N/A	N/A
Virtanen, 2015.<br><br>Finland	Voluntary national sample of secondary school students in Finland	Finish school Health Promotion Study	Cross sectional	23182	Mean 15.4 yrs. (SD 0.63)	Girls 52%	Individual level SES:<br>High 55%;<br>Intermediate 24%;<br>Low 20%	Not reported	Community	Shortest distance to a FFO or supermarket from school	Euclidean distance from each school.<br><br>89% of outlets identified from Statistics Finland, remaining from manual internet search		Categorical (>500m, 101-500m, <=100m	Purchasing	Purchasing snacks from outside school	Self-complete questionnaire<br><br>No definition provided for snack<br><br>No validation details	Binary (Yes/No)	Logistic regression	Age; individual SES; pocket money; after school employment; school SES	N/A	Schools used as clustering units	N/A	N/A	N/A

Supplementary Table 8: Details of included intervention studies

General Information				Population					Intervention			Outcome				Statistical Methods					
**Author, year. Country**	**Setting**	**Study Name**	**Study Design**	**Sample Size**	**Age**	**Sex/ Gender**	**Socio-economic status**	**Race/ Ethnicity**	**Community or Consumer**	**Intervention Details**	**Food outlet type and definition (if provided)**	**Purchasing/ Diet**	**Outcome details**	**Assessment method**	**Outcome Type**	**Statistical Model**	**Individual level covariates**	**Area-level covariates**	**Clustering level**	**Mutual Adjustment for multiple food outlets**	**Sub-group analyses**
Lawman, 2015<br><br>USA	Deprived neighbourhoods, Philadelphia	Healthy Corner Store Initiative	Repeated cross-sectional intervention study with no control group	Baseline: 1370<br><br>Follow-up: 999	13-18 yrs	Girls: 41%<br><br>* For entire study sample not just adolescent participants	No details provided	No details provided	Consumer	Introduction of 4 new healthy foods (availability) (2 new products from 2 different groups: fresh F&V, canned / dried F&V, low fat diary, lean meats, whole grains) plus marketing campaign consisting of window, door and in-store banners, shelf labels and recipe cards. Staff also participated in training focusing on healthy product procurement, promotions and pricing.<br><br>Intervention implemented over 12 month period	Convenience stores= businesses <2000 sq ft that primarily sold food, had < 4 aisles and had only 1 cash register	Purchasing	Change in energy (kcal)in foods purchased at BL and FU	Researcher conducted CPA when exiting store	Continuous	Multi-level modelling	N/A	N/A	Store level clustering		
Shin, 2015<br><br>USA	14 randomly selected recreational centres based in Baltimore City	Baltimore Healthy Eating Zones	Cluster Randomised intevention with control	152	10-14 yrs	Girls: 57.1% (control) , 59.6%(intervention)	No details provided	100% African American	Consumer	Intervention aiming to increase the available and promote healthy food choices in convenience stores and takeaways within 0.5 miles of the study recreation centres. Three food outlets were recruited for each intervention centre<br><br>Food outlets were incentivised to stock healthy, affordable foods in 5 phases which focus on single healthy eating component; healthy beverages, healthy breakfast, cooking at home/ healthy lunch, healthy snacks and selecting more healthy options at takeaways. Changes to availability were accompanied by in-store promotions such as taste-testing, cooking demonstrations. giveaways, shelf labels and points of purchase healthy communications.<br><br>Interventions conducted over 8 month period.	Food outlets were located within 0.5 miles of the recreation centre. No definitions provided for convenience stores or takeaways	Purchasing	Change in frequency of: healthful food purchases and unhealthful food purchases	Data collected by Youth Impact Questionnaire. No validation details provided.	Continuous	Wilcoxon rank-sum test or paired t-test	None adjusted for in analysis of interest	N/A	No details		

Supplementary Table 9: Vote counting results for observational studies

Author, year	Food Outlet Type	Classification	Boundary type	Metric Type	Intensity Metric	Outcome	Food type	Sex	Result	Direction allocated for
An, 2012	Fast Food Outlets	Unhealthy	School	Buffer: count in 0.5 mile	Count/density	Diet	Fruit	All	IRR 1.007; SE 0.006	-non sig
An, 2012	Fast Food Outlets	Unhealthy	Home	Buffer: count in 0.5 mile	Count/density	Diet	Fruit	All	IRR 0.991; SE 0.007	+ non sig
An, 2012	Fast Food Outlets	Unhealthy	School and home	Buffer: count in 0.5 mile	Count/density	Diet	Fruit	All	IRR 1.00; SE 0.005	incon
An, 2012	Fast Food Outlets	Unhealthy	School	Buffer: count in 0.5 mile	Count/density	Diet	Vegetables	All	IRR 1.017; SE 0.008; p<0.05	- sig
An, 2012	Fast Food Outlets	Unhealthy	Home	Buffer: count in 0.5 mile	Count/density	Diet	Vegetables	All	IRR 1.004; SE 0.008	-non sig
An, 2012	Fast Food Outlets	Unhealthy	School and home	Buffer: count in 0.5 mile	Count/density	Diet	Vegetables	All	IRR 1.012; SE 0.006; p<0.05	- sig
An, 2012	Fast Food Outlets	Unhealthy	School	Buffer: count in 0.5 mile	Count/density	Diet	100% Juice	All	IRR 0.994; SE 0.007	+ non sig
An, 2012	Fast Food Outlets	Unhealthy	Home	Buffer: count in 0.5 mile	Count/density	Diet	100% Juice	All	IRR 1.000; SE 0.009	incon
An, 2012	Fast Food Outlets	Unhealthy	School and home	Buffer: count in 0.5 mile	Count/density	Diet	100% Juice	All	IRR 0.997; SE 0.007	+ non sig
An, 2012	Fast Food Outlets	Unhealthy	School	Buffer: count in 0.5 mile	Count/density	Diet	Soda	All	IRR 0.989; SE 0.011	-non sig
An, 2012	Fast Food Outlets	Unhealthy	Home	Buffer: count in 0.5 mile	Count/density	Diet	Soda	All	IRR 1.005; SE 0.009	+ non sig
An, 2012	Fast Food Outlets	Unhealthy	School and home	Buffer: count in 0.5 mile	Count/density	Diet	Soda	All	IRR 0.997; SE 0.008	-non sig
An, 2012	Fast Food Outlets	Unhealthy	School	Buffer: count in 0.5 mile	Count/density	Diet	High-sugar food	All	IRR 1.029; SE 0.016	+ sig
An, 2012	Fast Food Outlets	Unhealthy	Home	Buffer: count in 0.5 mile	Count/density	Diet	High-sugar food	All	IRR 0.983; SE 0.011	-non sig
An, 2012	Fast Food Outlets	Unhealthy	School and home	Buffer: count in 0.5 mile	Count/density	Diet	High-sugar food	All	IRR 1.007; SE 0.020	+ non sig
An, 2012	Fast Food Outlets	Unhealthy	School	Buffer: count in 0.5 mile	Count/density	Diet	Fast Food	All	IRR 0.993; SE 0.012	-non sig
An, 2012	Fast Food Outlets	Unhealthy	Home	Buffer: count in 0.5 mile	Count/density	Diet	Fast Food	All	IRR 1.006; SE 0.012	+ non sig

An, 2012	Fast Food Outlets	Unhealthy	School and home	Buffer: count in 0.5 mile	Count/density	Diet	Fast Food	All	IRR 0.995; SE 0.008	-non sig
An, 2012	Convenience stores	Unhealthy	School	Buffer: count in 0.5 mile	Count/density	Diet	Fruit	All	IRR 1.000; SE 0.021	incon
An, 2012	Convenience stores	Unhealthy	Home	Buffer: count in 0.5 mile	Count/density	Diet	Fruit	All	IRR 1.006; SE 0.020	-non sig
An, 2012	Convenience stores	Unhealthy	School and home	Buffer: count in 0.5 mile	Count/density	Diet	Fruit	All	IRR 1.008; SE 0.015	-non sig
An, 2012	Convenience stores	Unhealthy	School	Buffer: count in 0.5 mile	Count/density	Diet	Vegetables	All	IRR 0.987; SE 0.026	+ non sig
An, 2012	Convenience stores	Unhealthy	Home	Buffer: count in 0.5 mile	Count/density	Diet	Vegetables	All	IRR 0.988; SE 0.022	+ non sig
An, 2012	Convenience stores	Unhealthy	School and home	Buffer: count in 0.5 mile	Count/density	Diet	Vegetables	All	IRR 0.986; SE 0.017	+ non sig
An, 2012	Convenience stores	Unhealthy	School	Buffer: count in 0.5 mile	Count/density	Diet	100% Juice	All	IRR 0.930; SE 0.032, p<0.05	+ sig
An, 2012	Convenience stores	Unhealthy	Home	Buffer: count in 0.5 mile	Count/density	Diet	100% Juice	All	IRR 0.979; SE 0.027	+ non sig
An, 2012	Convenience stores	Unhealthy	School and home	Buffer: count in 0.5 mile	Count/density	Diet	100% Juice	All	IRR 0.961; SE 0.021	+ non sig
An, 2012	Convenience stores	Unhealthy	School	Buffer: count in 0.5 mile	Count/density	Diet	Soda	All	IRR 0.984; SE 0.039	-non sig
An, 2012	Convenience stores	Unhealthy	Home	Buffer: count in 0.5 mile	Count/density	Diet	Soda	All	IRR 0.936; SE 0.031, p<0.05	- sig
An, 2012	Convenience stores	Unhealthy	School and home	Buffer: count in 0.5 mile	Count/density	Diet	Soda	All	IRR 0.952; SE 0.024,p<0.05	- sig
An, 2012	Convenience stores	Unhealthy	School	Buffer: count in 0.5 mile	Count/density	Diet	High-sugar food	All	IRR 1.051; SE 0.055	+ non sig
An, 2012	Convenience stores	Unhealthy	Home	Buffer: count in 0.5 mile	Count/density	Diet	High-sugar food	All	IRR 0.986; SE 0.043	-non sig
An, 2012	Convenience stores	Unhealthy	School and home	Buffer: count in 0.5 mile	Count/density	Diet	High-sugar food	All	IRR 1.025; SE 0.041	+ sig
An, 2012	Convenience stores	Unhealthy	School	Buffer: count in 0.5 mile	Count/density	Diet	Fast Food	All	IRR 1.005; SE 0.032	+ non sig
An, 2012	Convenience stores	Unhealthy	Home	Buffer: count in 0.5 mile	Count/density	Diet	Fast Food	All	IRR 0.935; SE 0.031, p<0.05	- sig

An, 2012	Convenience stores	Unhealthy	School and home	Buffer: count in 0.5 mile	Count/density	Diet	Fast Food	All	IRR 0.965; SE 0.023	-non sig
An, 2012	Small Food Stores	Unhealthy	School	Buffer: count in 0.5 mile	Count/density	Diet	Fruit	All	IRR 0.996; SE 0.007	+ non sig
An, 2012	Small Food Stores	Unhealthy	Home	Buffer: count in 0.5 mile	Count/density	Diet	Fruit	All	IRR 0.992; SE 0.006	+ non sig
An, 2012	Small Food Stores	Unhealthy	School and home	Buffer: count in 0.5 mile	Count/density	Diet	Fruit	All	IRR 0.995; SE 0.004	+ non sig
An, 2012	Small Food Stores	Unhealthy	School	Buffer: count in 0.5 mile	Count/density	Diet	Vegetables	All	IRR 1.002; SE 0.010	-non sig
An, 2012	Small Food Stores	Unhealthy	Home	Buffer: count in 0.5 mile	Count/density	Diet	Vegetables	All	IRR 0.998; SE 0.005	+ non sig
An, 2012	Small Food Stores	Unhealthy	School and home	Buffer: count in 0.5 mile	Count/density	Diet	Vegetables	All	IRR 1.001; SE 0.004	-non sig
An, 2012	Small Food Stores	Unhealthy	School	Buffer: count in 0.5 mile	Count/density	Diet	100% Juice	All	IRR 0.988; SE 0.010	+ non sig
An, 2012	Small Food Stores	Unhealthy	Home	Buffer: count in 0.5 mile	Count/density	Diet	100% Juice	All	IRR 1.004; SE 0.006	-non sig
An, 2012	Small Food Stores	Unhealthy	School and home	Buffer: count in 0.5 mile	Count/density	Diet	100% Juice	All	IRR 0.999; SE 0.005	+ non sig
An, 2012	Small Food Stores	Unhealthy	School	Buffer: count in 0.5 mile	Count/density	Diet	Soda	All	IRR 1.002; SE 0.009	+ non sig
An, 2012	Small Food Stores	Unhealthy	Home	Buffer: count in 0.5 mile	Count/density	Diet	Soda	All	IRR 1.009; SE 0.006	+ non sig
An, 2012	Small Food Stores	Unhealthy	School and home	Buffer: count in 0.5 mile	Count/density	Diet	Soda	All	IRR 1.004; SE 0.005	+ non sig
An, 2012	Small Food Stores	Unhealthy	School	Buffer: count in 0.5 mile	Count/density	Diet	High-sugar food	All	IRR 1.013; SE 0.015	+ non sig
An, 2012	Small Food Stores	Unhealthy	Home	Buffer: count in 0.5 mile	Count/density	Diet	High-sugar food	All	IRR 1.008; SE 0.008	+ non sig
An, 2012	Small Food Stores	Unhealthy	School and home	Buffer: count in 0.5 mile	Count/density	Diet	High-sugar food	All	IRR 1.008; SE 0.007	+ non sig
An, 2012	Small Food Stores	Unhealthy	School	Buffer: count in 0.5 mile	Count/density	Diet	Fast Food	All	IRR 1.010; SE 0.009	+ non sig
An, 2012	Small Food Stores	Unhealthy	Home	Buffer: count in 0.5 mile	Count/density	Diet	Fast Food	All	IRR 1.017; SE 0.005, p<0.01	+ sig

An, 2012	Small Food Stores	Unhealthy	School and home	Buffer: count in 0.5 mile	Count/density	Diet	Fast Food	All	IRR 1.009; SE 0.005	+ non sig
An, 2012	Grocery stores	Healthy	School	Buffer: count in 0.5 mile	Count/density	Diet	Fruit	All	IRR 0.962; SE 0.028	- non sig
An, 2012	Grocery stores	Healthy	Home	Buffer: count in 0.5 mile	Count/density	Diet	Fruit	All	IRR 0.982; SE 0.028	- non sig
An, 2012	Grocery stores	Healthy	School and home	Buffer: count in 0.5 mile	Count/density	Diet	Fruit	All	IRR 0.980; SE 0.019	- non sig
An, 2012	Grocery stores	Healthy	School	Buffer: count in 0.5 mile	Count/density	Diet	Vegetables	All	IRR 0.995; SE 0.029	- non sig
An, 2012	Grocery stores	Healthy	Home	Buffer: count in 0.5 mile	Count/density	Diet	Vegetables	All	IRR 0.971; SE 0.028	- non sig
An, 2012	Grocery stores	Healthy	School and home	Buffer: count in 0.5 mile	Count/density	Diet	Vegetables	All	IRR 0.988; SE 0.020	- non sig
An, 2012	Grocery stores	Healthy	School	Buffer: count in 0.5 mile	Count/density	Diet	100% Juice	All	IRR 0.947; SE 0.036	- non sig
An, 2012	Grocery stores	Healthy	Home	Buffer: count in 0.5 mile	Count/density	Diet	100% Juice	All	IRR 0.966; SE 0.038	- non sig
An, 2012	Grocery stores	Healthy	School and home	Buffer: count in 0.5 mile	Count/density	Diet	100% Juice	All	IRR 0.9688; SE 0.028	- non sig
An, 2012	Grocery stores	Healthy	School	Buffer: count in 0.5 mile	Count/density	Diet	Soda	All	IRR 1.023; SE 0.036	- non sig
An, 2012	Grocery stores	Healthy	Home	Buffer: count in 0.5 mile	Count/density	Diet	Soda	All	IRR 1.088; SE 0.031, $p<0.01$	- sig
An, 2012	Grocery stores	Healthy	School and home	Buffer: count in 0.5 mile	Count/density	Diet	Soda	All	IRR 1.050; SE 0.025	- non sig
An, 2012	Grocery stores	Healthy	School	Buffer: count in 0.5 mile	Count/density	Diet	High-sugar food	All	IRR 0.960; SE 0.047	+ non sig
An, 2012	Grocery stores	Healthy	Home	Buffer: count in 0.5 mile	Count/density	Diet	High-sugar food	All	IRR 1.019; SE 0.048	- non sig
An, 2012	Grocery stores	Healthy	School and home	Buffer: count in 0.5 mile	Count/density	Diet	High-sugar food	All	IRR 0.988; SE 0.037	+ non sig
An, 2012	Grocery stores	Healthy	School	Buffer: count in 0.5 mile	Count/density	Diet	Fast Food	All	IRR 1.042; SE 0.043	- non sig
An, 2012	Grocery stores	Healthy	Home	Buffer: count in 0.5 mile	Count/density	Diet	Fast Food	All	IRR 1.083; SE 0.034, $p<0.05$	- sig

An, 2012	Grocery stores	Healthy	School and home	Buffer: count in 0.5 mile	Count/density	Diet	Fast Food	All	IRR 1.050; SE 0.029	- non sig
An, 2012	Supermarket (Large)	Healthy	School	Buffer: count in 0.5 mile	Count/density	Diet	Fruit	All	IRR 1.020; SE 0.021	+ non sig
An, 2012	Supermarket (Large)	Healthy	Home	Buffer: count in 0.5 mile	Count/density	Diet	Fruit	All	IRR 1.016; SE 0.025	+ non sig
An, 2012	Supermarket (Large)	Healthy	School and home	Buffer: count in 0.5 mile	Count/density	Diet	Fruit	All	IRR 0.016; SE 0.017	- non sig
An, 2012	Supermarket (Large)	Healthy	School	Buffer: count in 0.5 mile	Count/density	Diet	Vegetables	All	IRR 1.001; SE 0.026	+ non sig
An, 2012	Supermarket (Large)	Healthy	Home	Buffer: count in 0.5 mile	Count/density	Diet	Vegetables	All	IRR 1.026; SE 0.024	+ non sig
An, 2012	Supermarket (Large)	Healthy	School and home	Buffer: count in 0.5 mile	Count/density	Diet	Vegetables	All	IRR 1.008; SE 0.018	+ non sig
An, 2012	Supermarket (Large)	Healthy	School	Buffer: count in 0.5 mile	Count/density	Diet	100% Juice	All	IRR 1.006; SE 0.029	+ non sig
An, 2012	Supermarket (Large)	Healthy	Home	Buffer: count in 0.5 mile	Count/density	Diet	100% Juice	All	IRR 1.011; SE 0.030	+ non sig
An, 2012	Supermarket (Large)	Healthy	School and home	Buffer: count in 0.5 mile	Count/density	Diet	100% Juice	All	IRR 1.000; SE 0.022	incon
An, 2012	Supermarket (Large)	Healthy	School	Buffer: count in 0.5 mile	Count/density	Diet	Soda	All	IRR 1.038; SE 0.039	- non sig
An, 2012	Supermarket (Large)	Healthy	Home	Buffer: count in 0.5 mile	Count/density	Diet	Soda	All	IRR 0.988; SE 0.032	+ non sig
An, 2012	Supermarket (Large)	Healthy	School and home	Buffer: count in 0.5 mile	Count/density	Diet	Soda	All	IRR 1.001; SE 0.026	- non sig
An, 2012	Supermarket (Large)	Healthy	School	Buffer: count in 0.5 mile	Count/density	Diet	High-sugar food	All	IRR 1.033; SE 0.040	- non sig
An, 2012	Supermarket (Large)	Healthy	Home	Buffer: count in 0.5 mile	Count/density	Diet	High-sugar food	All	IRR 0.928; SE 0.045	+ non sig
An, 2012	Supermarket (Large)	Healthy	School and home	Buffer: count in 0.5 mile	Count/density	Diet	High-sugar food	All	IRR 0.967; SE 0.032	+ non sig
An, 2012	Supermarket (Large)	Healthy	School	Buffer: count in 0.5 mile	Count/density	Diet	Fast Food	All	IRR 1.060; SE 0.036	- non sig
An, 2012	Supermarket (Large)	Healthy	Home	Buffer: count in 0.5 mile	Count/density	Diet	Fast Food	All	IRR 0.930; SE 0.036; $p<0.05$	+ sig

An, 2012	Supermarket (Large)	Healthy	School and home	Buffer: count in 0.5 mile	Count/density	Diet	Fast Food	All	IRR 0.993; SE 0.026	- non sig
Berge, 2014	Fast Food Outlets	Unhealthy	Home	Buffer: presence/abse	Proximity: pres	Diet	F&V	Boys	? 0.25; SE 0.16;	-non sig
Berge, 2014	Fast Food Outlets	Unhealthy	Home	Buffer: presence/abse	Proximity: pres	Diet	F&V	Girls	? -0.04; se 0.14	+ non sig
Berge, 2014	Supermarket	Healthy	Home	Buffer: presence/abse	Proximity: pres	Diet	F&V	Boys	? 0.14; SE 0.13;	+ non sig
Berge, 2014	Supermarket	Healthy	Home	Buffer: presence/abse	Proximity: pres	Diet	F&V	Girls	? 0.20; SE 0.12;	+ non sig
Berge, 2014	Convenience store:	Unhealthy	Home	Buffer: presence/abse	Proximity: pres	Diet	F&V	Boys	? 0.46, SE 0.20;	-non sig
Berge, 2014	Convenience store:	Unhealthy	Home	Buffer: presence/abse	Proximity: pres	Diet	F&V	Girls	? 0.023; SE 0.1	-non sig
Berge, 2014	Fast Food Outlets	Unhealthy	Home	Density: >5 outlets in	Count/density	Diet	F&V	Boys	? 0.13, SE 0.12;	-non sig
Berge, 2014	Fast Food Outlets	Unhealthy	Home	Density: >5 outlets in	Count/density	Diet	F&V	Girls	? -0.21; se 0.12	+ non sig
Berge, 2014	Unsupportive Envi	Unhealthy	Home	High density of FF+ FF and	Healthful envir	Diet	F&V	Boys	? 0.09; SE 0.06,	-non sig
Berge, 2014	Unsupportive Envi	Unhealthy	Home	High density of FF+ FF and	Healthful envir	Diet	F&V	Girls	? -0.06; SE 0.06	+ non sig
Berge, 2014	Fast Food Outlets	Unhealthy	Home	Buffer: presence/abse	Proximity: pres	Diet	Fast Food	Boys	? 0.65; SE 0.27;	+ non sig
Berge, 2014	Fast Food Outlets	Unhealthy	Home	Buffer: presence/abse	Proximity: pres	Diet	Fast Food	Girls	? -0.09; SE 0.25	-non sig
Berge, 2014	Supermarket	Healthy	Home	Buffer: presence/abse	Proximity: pres	Diet	Fast Food	Boys	? 0.06; SE 0.23;	- non sig
Berge, 2014	Supermarket	Healthy	Home	Buffer: presence/abse	Proximity: pres	Diet	Fast Food	Girls	? -0.23; SE 0.33	+ non sig
Berge, 2014	Convenience store:	Unhealthy	Home	Buffer: presence/abse	Proximity: pres	Diet	Fast Food	Boys	? 0.10; SE 0.36	+ non sig
Berge, 2014	Convenience store:	Unhealthy	Home	Buffer: presence/abse	Proximity: pres	Diet	Fast Food	Girls	? -0.15; SE 0.33	-non sig
Berge, 2014	Fast Food Outlets	Unhealthy	Home	Density: >5 outlets in	Count/density	Diet	Fast Food	Boys	? 0.08; SE 0.23;	+ non sig

Berge, 2014	Fast Food Outlets	Unhealthy	Home	Density: >5 outlets in	Count/density	Diet	Fast Food	Girls	? -0.28; SE 0.22	-non sig
Berge, 2014	Unsupportive Envi	Unhealthy	Home	High density of FF+ FF and	Healthful envir	Diet	Fast Food	Boys	? 0.13; SE0.11;	+ non sig
Berge, 2014	Unsupportive Envi	Unhealthy	Home	High density of FF+ FF and	Healthful envir	Diet	Fast Food	Girls	? -0.05; SE 0.11	-non sig
Clark, 2014	All food outlets	Total/Unhealthy	School	Count/Density : within 800m	Count/density	Diet	Diet Quality Inc	All	? 1.36 (95CI -1.	-non sig
Clark, 2014	Convenience stores	Unhealthy	School	Count/Density : within 800m	Count/density	Diet	Diet Quality Inc	All	? 1.36 (95CI -1.	-non sig
Clark, 2014	Restaurants	Neutral	School	Count/Density : within 800m	Count/density	Diet	Diet Quality Inc	All	? 1.51 (95CI -0.	n/a
Clark, 2014	Supermarket	Healthy	School	Count/Density : within 800m	Count/density	Diet	Diet Quality Inc	All	? 2.01 (95CI -1.	+ non sig
Clark, 2014	Fast Food Outlets	Unhealthy	School	Count/Density : within 800m	Count/density	Diet	Diet Quality Inc	All	? 1.51 (95ci -0.	-non sig
Clark, 2014	All food outlets	Total/Unhealthy	School	Count/Density : within	Count/density	Diet	Diet Quality Inc	All	? 2.51 (95CI -0.	-non sig
Clark, 2014	Convenience stores	Unhealthy	School	Count/Density : within	Count/density	Diet	Diet Quality Inc	All	? 2.12 (95CI -0.	-non sig
Clark, 2014	Restaurants	Neutral	School	Count/Density : within	Count/density	Diet	Diet Quality Inc	All	?2.41 (95CI -0.3	n/a
Clark, 2014	Supermarket	Healthy	School	Count/Density : within	Count/density	Diet	Diet Quality Inc	All	? 1.51 (95ci -0.	+ non sig
Clark, 2014	Fast Food Outlets	Unhealthy	School	Count/Density : within	Count/density	Diet	Diet Quality Inc	All	? 1.75 (95CI -1.	-non sig
Clark, 2014	All food outlets	Total/Unhealthy	School	Distance: from school to	Proximity	Diet	Diet Quality Inc	All	? -1.01 (95ci -1.	-non sig
Clark, 2014	Convenience stores	Unhealthy	School	Distance: from school to	Proximity	Diet	Diet Quality Inc	All	? -0.70 (-1.32, -	+ sig
Clark, 2014	Restaurants	Neutral	School	Distance: from school to	Distance	Diet	Diet Quality Inc	All	? 0.00 (95CI -0.	n/a
Clark, 2014	Supermarket	Healthy	School	Distance: from school to	Proximity	Diet	Diet Quality Inc	All	? 0.00 (95CI -0.	incon
Clark, 2014	Fast Food Outlets	Unhealthy	School	Distance: from school to	Count/density	Diet	Diet Quality Inc	All	? -0.56 (95ci -1.	+ non sig

Cutumisu, 2016	Fast Food Outlets	Unhealthy	School	High vs Low number FF	Count/density:	Diet	Junk Food cons	All	OR 1.50, CI95%	+ sig
Davis, 2009	Fast Food Outlets	Unhealthy	School	Presence (vs none) of FF	Proximity: pres	Diet	Vegetables (Ye:	All	AOR 0.97 (0.93,	+ non sig
Davis, 2009	Fast Food Outlets	Unhealthy	School	Presence (vs none) of FF	Proximity: pres	Diet	Fruit (Yes/No)	All	AOR 0.97 (0.93,	+ non sig
Davis, 2009	Fast Food Outlets	Unhealthy	School	Presence (vs none) of FF	Proximity: pres	Diet	Juice (Yes/ No)	All	AOR 0.97 (0.94,	+ non sig
Davis, 2009	Fast Food Outlets	Unhealthy	School	Presence (vs none) of FF	Proximity: pres	Diet	Soda (Yes/No)	All	AOR 1.05 (1.00,	+ sig
Davis, 2009	Fast Food Outlets	Unhealthy	School	Presence (vs none) of FF	Proximity: pres	Diet	Fried Potato (Y	All	AOR 1.01 (0.98,	+ non sig
Davis, 2009	Fast Food Outlets	Unhealthy	School	Presence (vs none) of FF	Proximity: pres	Diet	no. servings of	All	? -0.02 (-0.03, (	+ sig
Davis, 2009	Fast Food Outlets	Unhealthy	School	Presence (vs none) of FF	Proximity: pres	Diet	no. servings of	All	? -0.02 (-0.04, (	+ sig
Davis, 2009	Fast Food Outlets	Unhealthy	School	Presence (vs none) of FF	Proximity: pres	Diet	no.servings of j	All	? -0.02 (-0.03, (	+ sig
Davis, 2009	Fast Food Outlets	Unhealthy	School	Presence (vs none) of FF	Proximity: pres	Diet	no. servings of	All	? 0.02 (-0.01, 0.	+ non sig
Davis, 2009	Fast Food Outlets	Unhealthy	School	Presence (vs none) of FF	Proximity: pres	Diet	no. servings of	All	? 0.00 (-0.02, 0.	+ non sig
Edmonds, 2001	Grocery stores	Consumer	n/a	Availability of fruit, veg and	Consumer: Availability	Diet	Fruit	Boys	r -0.17; p=0.64	- non sig
Edmonds, 2001	Grocery stores	Consumer	n/a	Availability of fruit, veg and	Consumer: Availability	Diet	Vegetables	Boys	r 0.02; p=0.51	+ non sig
Edmonds, 2001	Grocery stores	Consumer	n/a	Availability of fruit, veg and	Consumer: Availability	Diet	100% Juice	Boys	r 0.48; p=0.14	+ non sig
Edmonds, 2001	Restaurants	Consumer	n/a	Availability of fruit, veg and	Consumer: Availability	Diet	Fruit	Boys	r -0.33; p=0.39	- non sig
Edmonds, 2001	Restaurants	Consumer	n/a	Availability of fruit, veg and	Consumer: Availability	Diet	Vegetables	Boys	r 0.72; p=0.03	+ sig
Edmonds, 2001	Restaurants	Consumer	n/a	Availability of fruit, veg and	Consumer: Availability	Diet	100% Juice	Boys	r 0.70; p=0.04	+ sig
Forsyth, 2012	Fast Food Outlets	Unhealthy	Home	Count/Density : within	Count/Density	Diet	Fast food consumption	Boys	? 0.059	+ non sig

Forsyth, 2012	Fast Food Outlets	Unhealthy	Home	Count/Density : within	Count/Density	Diet	Fast food consumption	Girls		? 0.632	+ non sig
Forsyth, 2012	Fast Food Outlets	Unhealthy	Home	Count/Density : High (11+)	Count/Density	Diet	Fast food consumption	Boys		? 0.044; p<0.05	+ sig
Forsyth, 2012	Fast Food Outlets	Unhealthy	Home	Count/Density : High (11+)	Count/Density	Diet	Fast food consumption	Girls		? 0.712	+ non sig
Forsyth, 2012	Fast Food Outlets	Unhealthy	School	Count/Density : within 800m	Count/Density	Diet	Fast food consumption	Boys		? 0.644	+ non sig
Forsyth, 2012	Fast Food Outlets	Unhealthy	School	Count/Density : within 800m	Count/Density	Diet	Fast food consumption	Girls		? 0.299	+ non sig
Godin, 2018	Fast Food Outlets	Unhealthy	School	Buffer: presence/abse	Proximity: pres	Diet	SSB consumption	All		Exponentiated	+ non sig
Godin, 2018	Variety Stores	Unhealthy	School	Buffer: presence/abse	Proximity: pres	Diet	SSB consumption	All		Exponentiated	+ non sig
Godin, 2018	All food outlets	Total/Unhealthy	School	Buffer: presence/abse	Proximity: pres	Diet	SSB consumption	All		Exponentiated	+ non sig
Grier and Davis, 2013	Fast Food Outlets	Unhealthy	School	Proximity: Distance from	Proximity	Diet	SSB consumption	All		? 0.01 (OR 1.00	+ non sig
Gustafson, 2017	NEMS scores of 3 top visited food	Consumer	n/a	Consumer Food NEMS	Consumer: Availability	Diet	Daily consumption	All		? -0.001 (95%C	- non sig
Gustafson, 2017	NEMS scores of 3 top visited food	Consumer	n/a	Consumer Food NEMS	Consumer: Availability	Diet	Daily consumption	All		?-0.002 (95%CI	+ non sig
Gustafson, 2017	NEMS scores of 3 top visited food	Consumer	n/a	Consumer Food NEMS	Consumer: Availability	Diet	Daily consumption	All		? 0.0002 (95%C	- non sig
Hager, 2017	Supermarket	Healthy	Home	Living in food desert: no	Proximity: pres	Diet	Fruit	Girls		Difference of 0.05 in mean	- non sig
Hager, 2017	Supermarket	Healthy	Home	Living in food desert: no	Proximity: pres	Diet	Vegetables	Girls		Difference of 0.13 in mean	- non sig
Hager, 2017	Convenience stores and corner	Unhealthy	Home	Living in a food swamp:	Density	Diet	Fruit	Girls		Difference of 0.12 in mean	-non sig
Hager, 2017	Convenience stores and corner	Unhealthy	Home	1-3 stores within 0.4km	Density	Diet	Fruit	Girls		Difference of - 0.03 in mean	+ non sig
Hager, 2017	Unsupportive Environment	Unhealthy	Home	Living in food swamp and	Proximity and Density	Diet	Fruit	Girls		Difference of 0.08 in mean	-non sig
Hager, 2017	Convenience stores and corner	Unhealthy	Home	Living in a food swamp:	Density	Diet	Vegetables	Girls		Difference of 0.24 in mean	-non sig

Hager, 2017	Convenience stores and corner	Unhealthy	Home	1-3 stores within 0.4km	Density	Diet	Vegetables	Girls	Difference of 0.13 in mean	-non sig
Hager, 2017	Unsupportive Environment	Unhealthy	Home	Living in food swamp and	Proximity and Density	Diet	Vegetables	Girls	Difference of 0.24 in mean	-non sig
Hager, 2017	Supermarket	Healthy	Home	Living in food desert: no	Proximity	Diet	Snacks and Dessets	Girls	? 0.05, 95%CI - 0.09, 0.19,	+ non sig
Hager, 2017	Convenience stores and corner	Unhealthy	Home	Living in a food swamp:	Density	Diet	Snacks and Dessets	Girls	? 0.11, 95%CI - 0.001, 0.22;	+ non sig
Hager, 2017	Unsupportive Environment	Unhealthy	Home	Living in food swamp and	Proximity and Density	Diet	Snacks and Dessets	Girls	? 0.13, 95%CI 0.01, 0.12;	+ non sig
He, 2012a	Fast Food Outlets	Unhealthy	Home	1-2 fast food outlets vs	Density	Purchasing	Self purchasing of	All	OR 1.6, 95%CI 1.1, 2.3;	+ sig
He, 2012a	Fast Food Outlets	Unhealthy	Home	3 + fast food outlets vs	Density	Purchasing	Self purchasing of	All	OR 1.7, 95%CI 1.1, 2.6;	+ sig
He, 2012a	Fast Food Outlets	Unhealthy	Home	Closest FF outlet <=1km	Proximity	Purchasing	Self purchasing of	All	OR 1.5, 95%CI 1.1-2.1;	+ sig
He, 2012a	Convenience stores	Unhealthy	Home	Closest convenience	Proximity	Purchasing	Self purchasing	All	OR 2.5, 95%CI 1.5, 3.6;	+ sig
He, 2012a	Fast Food Outlets	Unhealthy	School	Density of fast food outlets	Density	Purchasing	Self purchasing of	All	OR 1.4, 95%CI 1.1, 1.7:	+ sig
He, 2012b	Convenience stores	Unhealthy	Home	Proximity: Distance from	Proximity	Diet	Diet Quality modified HEI-		? 1.80, SE 0.79; P0.03	+ sig
He, 2012b	Fast Food Outlets	Unhealthy	Home	Proximity: Distance from	Proximity	Diet	Diet Quality modified HEI-		? 1.10, SE 0.64;	+ non sig
He, 2012b	Fast Food Outlets	Unhealthy	Home	Density: 1-2 FFO within	Density	Diet	Diet Quality modified HEI-		? -0.95, se 1.01	-non sig
He, 2012b	Fast Food Outlets	Unhealthy	Home	Density: 0 FFO within 1km	Density	Diet	Diet Quality modified HEI-		? 0.80, se 0.75;	+ non sig
He, 2012b	Supermarket	Healthy	Home	Proximity: Distance from	Proximity	Diet	Diet Quality modified HEI-		? 0.25, SE 0.88,	- non sig
He, 2012b	Supermarket	Healthy	Home	Proximity: Distance from	Proximity	Diet	Diet Quality modified HEI-		? 0.16, 0.92, P (	- non sig
He, 2012b	Convenience stores	Unhealthy	School	Proximity: Distance from	Proximity	Diet	Diet Quality modified HEI-		? 2.00, SE 1.00,	+ sig
He, 2012b	Fast Food Outlets	Unhealthy	School	Proximity: Distance from	Proximity	Diet	Diet Quality modified HEI-		? 2.60, SE 0.98,	+ sig

He, 2012b	Fast Food Outlets	Unhealthy	School	Density: 1-2 FFO within	Density	Diet	Diet Quality modified HEI-		? 0.66, SE 1.14,	+ non sig
He, 2012b	Fast Food Outlets	Unhealthy	School	Density: 0 FFO within 1km	Density	Diet	Diet Quality modified HEI-		? 2.75, SE 1.06	+ non sig
Jago, 2007	Fast Food Outlets	Unhealthy	Home	Distance from home to FFO	Distance	Diet	fruit and fruit juice	Boys	? 0.00; p=0.008	- sig
Jago, 2007	Small Food Stores	Unhealthy	Home	Distance from home to small	Distance	Diet	fruit and fruit juice	Boys	? -0.00; p=0.01:	- sig
Jago, 2007	Small Food Stores	Unhealthy	Home	Distance from home to small	Distance	Diet	Low fat veg	Boys	? 0.00; p=0.06	-non sig
Jago, 2007	Fast Food Outlets	Unhealthy	Home	Distance from home to FFO	Distance	Diet	High fat veg	Boys	? -0.01; p=0.00	- sig
Jago, 2007	Small Food Stores	Unhealthy	Home	Distance from home to small	Distance	Diet	High fat veg	Boys	? 0.03; p<0.001	- sig
Kelly, 2019	Fast Food Outlets	Unhealthy	School	> 10% of food outlets in 1km	Density	Diet	Fruit	All	OR 0.78 (95%CI	+ sig
Kelly, 2019	Fast Food Outlets	Unhealthy	School	> 10% of food outlets in 1km	Density	Diet	Vegetables	All	OR 0.79 (95%CI	+ sig
Kelly, 2019	Fast Food Outlets	Unhealthy	School	> 10% of food outlets in 1km	Density	Diet	Sweets	All	OR 0.94 (95%CI	-non sig
Kelly, 2019	Fast Food Outlets	Unhealthy	School	> 10% of food outlets in 1km	Density	Diet	SSB	All	OR 1.00 (95%CI	incon
Kelly, 2019	Fast Food Outlets	Unhealthy	School	> 10% of food outlets in 1km	Density	Diet	Chips	All	OR 1.03 (95%CI	+ non sig
Khan, 2012	Fast food price	Consumer	Consumer	FF price index in local zip	Consumer: Price	Diet	FF consumption		? -0.527 (SE 0.2	+ sig
Laska, 2010	Fast Food Outlets	Unhealthy	;chool and hom	Number of FFO within	Density	Diet	SSB	All	? 0.25 (95%CI 0	+ sig
Laska, 2010	Restaurants	Neutral	;chool and hom	Distance to nearest	Distance	Diet	SSB	All	? -0.007 (95%C	n/a
Laska, 2010	Restaurants	Neutral	;chool and hom	Number of restaurants	Density	Diet	SSB	All	? 0.28 (95%CI (	n/a
Laska, 2010	Restaurants	Neutral	;chool and hom	Number of restaurants	Density	Diet	SSB	All	? 0.23 (95%CI 0	n/a
Laska, 2010	Convenience store:	Unhealthy	;chool and hom	Number of convenience	Density	Diet	SSB	All	? 0.24 (95%CI 0	+ sig

Laska, 2010	Grocery stores	Healthy	;chool and hom	Distance to nearest	Distance	Diet	SSB	All	? -0.005 (95%C	+ sig
Laska, 2010	Grocery stores	Healthy	;chool and hom	Number of grocery stores	Density	Diet	SSB	All	? 0.31 (95%CI 0	- sig
Laska, 2010	All retail	Total/Unhealthy	;chool and hom	Number of retail facilities	Density	Diet	SSB	All	? 0.24 (95%CI 0	+ sig
Laxer, 2013	Fast Food Outlets	Unhealthy	School	Density: Low density around school	Density	Diet	Excessive fast food consumption	All	OR 1.40 (95%CI 0.87, 2.26)	+ non sig
Laxer, 2013	Fast Food Outlets	Unhealthy	School	Density: Moderate density	Density	Diet	Excessive fast food consumption	All	OR 1.68 (95%CI 1.11, 2.54)	+ sig
Laxer, 2013	Fast Food Outlets	Unhealthy	School	Density: High density	Density	Diet	Excessive fast food	All	OR 1.70 (95%CI 1.12,	+ sig
Loh, 2022	All food outlets	Total/Unhealthy	Home	Living in area characterised by high variety	Count/density	Purchasing	Snack food (? once/week- to school)	All	OR 1.09 (95%CI 0.24, 4.76)	-non sig
Loh, 2022	All food outlets	Total/Unhealthy	Home	Living in area characterised by some	Count/density	Purchasing	Snack food (? once/week- to school)	All	OR 0.86 (95% CI 0.19, 4.00)	+ non sig
Loh, 2022	All food outlets	Total/Unhealthy	Home	Living in area characterised by limited	Count/density	Purchasing	Snack food (? once/week- to school)	All	OR 1.07 (95% CI 0.18, 6.25)	-non sig
Loh, 2022	All food outlets	Total/Unhealthy	Home	Living in area characterised by high variety and medium	Count/density	Purchasing	Snack food (? once/week- from school)	All	OR 1.21 (95% CI 0.40, 3.66)	-non sig
Loh, 2022	All food outlets	Total/Unhealthy	Home	Living in area characterised by some	Count/density	Purchasing	Snack food (? once/week- from school)	All	OR 1.38 (95% CI 0.44, 4.29)	-non sig
Loh, 2022	All food outlets	Total/Unhealthy	Home	Living in area characterised by limited variety and	Count/density	Purchasing	Snack food (? once/week- from school)	All	OR 1.52 (95% CI 0.41, 5.57)	-non sig
Longacre, 2012	Fast Food Outlets	Unhealthy	In home town	1-4 FF outlets in town	Count/density	Diet	FF consumption	All	ARR 1.07 (95%CI 0.89,	+ non sig
Longacre, 2012	Fast Food Outlets	Unhealthy	In home town	5+ FF outlets in town	Count/density	Diet	FF consumption	All	ARR 1.29, 95% CI 1.10, 1.51	+ sig

Powell, 2011	Fast food price	Consumer	Consumer	FF price index in local zip	Consumer: Price	Diet	Fruit	All	? 0.1582 (SE 0.3877)	- non sig
Powell, 2011	Fast food price	Consumer	Consumer	FF price index in local zip	Consumer: Price	Diet	Vegetables	All	? 0.0710 (SE 0.3479)	- non sig
Powell, 2011	Fast food price	Consumer	Consumer	FF price index in local zip	Consumer: Price	Diet	Sweets	All	? - 0.0117 (SE 0.3491)	- non sig
Powell, 2011	Fast Food Outlets	Unhealthy	per 10,000 capita per 10	Number of fast food	Count/density	Diet	Fruit	All	? -0.0089 (SE 0.0367)	+ non sig
Powell, 2011	Fast Food Outlets	Unhealthy	per 10,000 capita per 10	Number of fast food	Count/density	Diet	Vegetables	All	? -0.0323 (SE 0.365)	+ non sig
Powell, 2011	Fast Food Outlets	Unhealthy	per 10,000 capita per 10	Number of fast food	Count/density	Diet	Sweets	All	? -0.0117 (SE 0.0359)	-non sig
Powell, 2011	Restaurants	Neutral	per 10,000 capita per 10	Number of restaurants	Count/density	Diet	Fruit	All	? 0.0067 (SE0.0087)	n/a
Powell, 2011	Restaurants	Neutral	per 10,000 capita per 10	Number of restaurants	Count/density	Diet	Vegetables	All	? -0.0024 (SE 0.0074)	n/a
Powell, 2011	Restaurants	Neutral	per 10,000 capita per 10	Number of restaurants	Count/density	Diet	Sweets	All	? 0.0075 (SE 0.0059)	n/a
Powell, 2011	Supermarkets	Healthy	per 10,000 capita per 10	Number of supermarkets	Count/density	Diet	Fruit	All	? 0.0010 (SE 0.0125)	+ non sig
Powell, 2011	Supermarkets	Healthy	per 10,000 capita per 10	Number of supermarkets	Count/density	Diet	Vegetables	All	? 0.0146 (SE 0.0112)	+ non sig
Powell, 2011	Supermarkets	Healthy	per 10,000 capita per 10	Number of supermarkets	Count/density	Diet	Sweets	All	? -0.0093 (SE 0.0073)	- non sig
Powell, 2011	Convenience stores	Unhealthy	per 10,000 capita per 10	Number of convenience	Count/density	Diet	Fruit	All	? -0.0138 (SE 0.0396)	+ non sig
Powell, 2011	Convenience stores	Unhealthy	per 10,000 capita per 10	Number of convenience	Count/density	Diet	Vegetables	All	? -0.0351 (SE 0.0467)	+ non sig
Powell, 2011	Convenience stores	Unhealthy	per 10,000 capita per 10	Number of convenience	Count/density	Diet	Sweets	All	? 0.0128 (SE 0.0342)	-non sig
Sadler, 2016	Fast Food Outlets	Unhealthy	Journey to and from school	Time spent exposured to	Time exposed	Purchasing	Junk Food	All	OR 1.174; (95%	+ sig
Seliske, 2013	Multiple types of	Unhealthy	School	Denisty: 1-2 food outlets	Count/density	Purchasing	Lunchtime purchasing at	All	OR 1.10 (95%CI	+ non sig
Seliske, 2013	Multiple types of	Unhealthy	School	Denisty: 3-4 food outlets	Count/density	Purchasing	Lunchtime purchasing at	All	OR 1.45 (95%CI	+ non sig

Seliske, 2013	Multiple types of u	Unhealthy	School	Denisty: 5+ food outlets	Count/density	Purchasing	Lunchtime purchasing at	All	OR 2.94 (95%CI	+ sig
Seliske, 2013	Multiple types of u	Unhealthy	School	Denisty: 1-2 food outlets	Count/density	Purchasing	Lunchtime purchasing at	All	OR 1.20 (95%CI	+ non sig
Seliske, 2013	Multiple types of u	Unhealthy	School	Denisty: 3-4 food outlets	Count/density	Purchasing	Lunchtime purchasing at	All	OR 3.19 (95%CI	+ sig
Seliske, 2013	Multiple types of u	Unhealthy	School	Denisty: 5+ food outlets	Count/density	Purchasing	Lunchtime purchasing at	All	OR 3.54 (95%CI	+ sig
Shareck, 2017	Fast Food Outlets	Unhealthy	Home	Availability of FFO within	Count	Diet	Fast food consumption	All	RR 1.00, (95%C	incon
Shareck, 2017	Fast Food Outlets	Unhealthy	School	Availability of FFO within	Count	Diet	Fast food consumption	All	RR 0.99, (95%C	-non sig
Shareck, 2017	Fast Food Outlets	Unhealthy	School and home	Availability of FFO within	Count	Diet	Fast food consumption	All	RR 1.00, (95%C	incon
Shareck, 2017	Convenience stores	Unhealthy	Home	Availability of convenience	Count	Diet	SSB consumption	All	RR 1.00, (95%C	incon
Shareck, 2017	Convenience stores	Unhealthy	School	Availability of convenience	Count	Diet	SSB consumption	All	RR 1.00, (95%C	incon
Shareck, 2017	Convenience stores	Unhealthy	School and home	Availability of convenience	Count	Diet	SSB consumption	All	RR 1.00, (95%C	incon
Shareck, 2017	Fast Food Outlets	Unhealthy	Home	Relative availability of	Proportion	Diet	Fast food consumption	All	RR 1.30, (95% C	+ non sig
Shareck, 2017	Fast Food Outlets	Unhealthy	School	Relative availability of	Proportion	Diet	Fast food consumption	All	RR 0.65, (95% C	-non sig
Shareck, 2017	Fast Food Outlets	Unhealthy	School and home	Relative availability of	Proportion	Diet	Fast food consumption	All	RR 0.65, (95% C	-non sig
Shareck, 2017	Convenience stores	Unhealthy	Home	Relative availability of	Proportion	Diet	SSB consumption	All	RR 1.45, (95% C	+ sig
Shareck, 2017	Convenience stores	Unhealthy	School	Relative availability of	Proportion	Diet	SSB consumption	All	RR 1.18, (95% C	+ non sig
Shareck, 2017	Convenience stores	Unhealthy	School and home	Relative availability of	Proportion	Diet	SSB consumption	All	RR 1.69, (95% C	+ sig
Shearer, 2015	Fast Food Outlets	Unhealthy	Home	Average distance to	Distance	Diet	Calories	All	r -0.05	+ non sig
Shearer, 2015	Fast Food Outlets	Unhealthy	Home	Average distance to	Distance	Diet	Diet quality	All	r 0.03	+ non sig

Shearer, 2015	Fast Food Outlets	Unhealthy	Home	Average distance to	Distance	Diet	F&V consumpti	All	r -0.01	-non sig
Shearer, 2015	Fast Food Outlets	Unhealthy	Home	Average distance to	Distance	Diet	Fast food consu	All	r -0.04	+ non sig
Shearer, 2015	Fast Food Outlets	Unhealthy	Home	Average distance to	Distance	Diet	Ready made fo	All	r -0.08	+ non sig
Shearer, 2015	Restaurants	Neutral	Home	Average distance to	Distance	Diet	Calories	All	r -0.04	n/a
Shearer, 2015	Restaurants	Neutral	Home	Average distance to	Distance	Diet	Diet quality	All	r 0.04	n/a
Shearer, 2015	Restaurants	Neutral	Home	Average distance to	Distance	Diet	F&V consumpti	All	r 0.03	n/a
Shearer, 2015	Restaurants	Neutral	Home	Average distance to	Distance	Diet	Fast food consu	All	r -0.08	n/a
Shearer, 2015	Restaurants	Neutral	Home	Average distance to	Distance	Diet	Ready made fo	All	r -0.01	n/a
Shearer, 2015	Grocery stores	Healthy	Home	Average distance to	Distance	Diet	Calories	All	r -0.01	- non sig
Shearer, 2015	Grocery stores	Healthy	Home	Average distance to	Distance	Diet	Diet quality	All	r 0.09	- non sig
Shearer, 2015	Grocery stores	Healthy	Home	Average distance to	Distance	Diet	F&V consumpti	All	r 0.05	- non sig
Shearer, 2015	Grocery stores	Healthy	Home	Average distance to	Distance	Diet	Fast food consu	All	r -0.01	- non sig
Shearer, 2015	Grocery stores	Healthy	Home	Average distance to	Distance	Diet	Ready made fo	All	r -0.06	- non sig
Shearer, 2015	Convenience stores	Unhealthy	Home	Average distance to	Distance	Diet	Calories	All	r -0.03	+ non sig
Shearer, 2015	Convenience stores	Unhealthy	Home	Average distance to	Distance	Diet	Diet quality	All	r 0.05	+ non sig
Shearer, 2015	Convenience stores	Unhealthy	Home	Average distance to	Distance	Diet	F&V consumpti	All	r 0.03	+ non sig
Shearer, 2015	Convenience stores	Unhealthy	Home	Average distance to	Distance	Diet	Fast food consu	All	r 0.06	-non sig
Shearer, 2015	Convenience stores	Unhealthy	Home	Average distance to	Distance	Diet	Ready made fo	All	r -0.00	incon

Shearer, 2015	Fast Food Outlets	Unhealthy	;PS activity spac	Average distance to	Distance	Diet	Calories	All	r -0.03	+ non sig
Shearer, 2015	Fast Food Outlets	Unhealthy	;PS activity spac	Average distance to	Distance	Diet	Diet quality	All	r 0.09	+ non sig
Shearer, 2015	Fast Food Outlets	Unhealthy	;PS activity spac	Average distance to	Distance	Diet	F&V consumpti	All	r 0.10	+ non sig
Shearer, 2015	Fast Food Outlets	Unhealthy	;PS activity spac	Average distance to	Distance	Diet	Fast food consu	All	r -0.09	+ non sig
Shearer, 2015	Fast Food Outlets	Unhealthy	;PS activity spac	Average distance to	Distance	Diet	Ready made fo	All	r 0.04	-non sig
Shearer, 2015	Restaurants	Neutral	;PS activity spac	Average distance to	Distance	Diet	Calories	All	r -0.03	n/a
Shearer, 2015	Restaurants	Neutral	;PS activity spac	Average distance to	Distance	Diet	Diet quality	All	r 0.08	n/a
Shearer, 2015	Restaurants	Neutral	;PS activity spac	Average distance to	Distance	Diet	F&V consumpti	All	r 0.07	n/a
Shearer, 2015	Restaurants	Neutral	;PS activity spac	Average distance to	Distance	Diet	Fast food consu	All	r -0.04	n/a
Shearer, 2015	Restaurants	Neutral	;PS activity spac	Average distance to	Distance	Diet	Ready made fo	All	r 0.04	n/a
Shearer, 2015	Grocery stores	Healthy	;PS activity spac	Average distance to	Distance	Diet	Calories	All	r -0.01	- non sig
Shearer, 2015	Grocery stores	Healthy	;PS activity spac	Average distance to	Distance	Diet	Diet quality	All	r 0.06	- non sig
Shearer, 2015	Grocery stores	Healthy	;PS activity spac	Average distance to	Distance	Diet	F&V consumpti	All	r 0.10	- non sig
Shearer, 2015	Grocery stores	Healthy	;PS activity spac	Average distance to	Distance	Diet	Fast food consu	All	r -0.02	- non sig
Shearer, 2015	Grocery stores	Healthy	;PS activity spac	Average distance to	Distance	Diet	Ready made fo	All	r 0.06	+ non sig
Shearer, 2015	Convenience store:	Unhealthy	;PS activity spac	Average distance to	Distance	Diet	Calories	All	r -0.02	+ non sig
Shearer, 2015	Convenience store:	Unhealthy	;PS activity spac	Average distance to	Distance	Diet	Diet quality	All	r 0.12	+ non sig
Shearer, 2015	Convenience store:	Unhealthy	;PS activity spac	Average distance to	Distance	Diet	F&V consumpti	All	r 0.14 **$p<0.05$**	+ sig

Shearer, 2015	Convenience stores	Unhealthy	GPS activity spac	Average distance to	Distance	Diet	Fast food consu	All	r -0.07	+ non sig
Shearer, 2015	Convenience stores	Unhealthy	GPS activity spac	Average distance to	Distance	Diet	Ready made fo	All	r 0.08	-non sig
Shier, 2016	Fast Food Outlets	Unhealthy	Home	Number of fast food	Count	Diet	Fruit	All	? 0.014, SE 0.03	-non sig
Shier, 2016	Fast Food Outlets	Unhealthy	Home	Number of fast food	Count	Diet	Vegetables	All	? 0.068, SE 0.03	- sig
Shier, 2016	Fast Food Outlets	Unhealthy	Home	Number of fast food	Count	Diet	Soda	All	? 0.006, SE 0.02	+ non sig
Shier, 2016	Fast Food Outlets	Unhealthy	Home	Number of fast food	Count	Diet	Sweet snacks	All	? -0.040, SE 0.0	-non sig
Shier, 2016	Fast Food Outlets	Unhealthy	Home	Number of fast food	Count	Diet	Salty snacks	All	? -0.056, SE 0.0	- sig
Shier, 2016	Fast Food Outlets	Unhealthy	Home	Number of fast food	Count	Diet	Ready made di	All	? 0.003 , SE 0.0	+ non sig
Shier, 2016	Restaurants	Neutral	Home	Number of restaurants	Count	Diet	Fruit	All	? 0.001, SE 0.01	n/a
Shier, 2016	Restaurants	Neutral	Home	Number of restaurants	Count	Diet	Vegetables	All	? 0.016, SE 0.01	n/a
Shier, 2016	Restaurants	Neutral	Home	Number of restaurants	Count	Diet	Soda	All	? 0.005, SE 0.00	n/a
Shier, 2016	Restaurants	Neutral	Home	Number of restaurants	Count	Diet	Sweet snacks	All	? 0.023, SE0.01	n/a
Shier, 2016	Restaurants	Neutral	Home	Number of restaurants	Count	Diet	Salty snacks	All	? -0.014, SE 0.0	n/a
Shier, 2016	Restaurants	Neutral	Home	Number of restaurants	Count	Diet	Ready made di	All	? -0.003, SE 0.0	n/a
Shier, 2016	Convenience stores	Unhealthy	Home	Number of convenience	Count	Diet	Fruit	All	? -0.011, SE 0.1	+ non sig
Shier, 2016	Convenience stores	Unhealthy	Home	Number of convenience	Count	Diet	Vegetables	All	? 0.254, SE 0.19	-non sig
Shier, 2016	Convenience stores	Unhealthy	Home	Number of convenience	Count	Diet	Soda	All	? 0.035, SE 0.21	+ non sig
Shier, 2016	Convenience stores	Unhealthy	Home	Number of convenience	Count	Diet	Sweet snacks	All	? - 0.358, SE0.4	-non sig

Shier, 2016	Convenience stores	Unhealthy	Home	Number of convenience	Count	Diet	Salty snacks	All	? -0.255, SE 0.2	-non sig
Shier, 2016	Convenience stores	Unhealthy	Home	Number of convenience	Count	Diet	Ready made di	All	? 0.026, SE 0.06	+ non sig
Shier, 2016	Small Food Stores	Unhealthy	Home	Number of small grocery	Count	Diet	Fruit	All	? -0.014, SE 0.0	-non sig
Shier, 2016	Small Food Stores	Unhealthy	Home	Number of small grocery	Count	Diet	Vegetables	All	? 0.033, SE 0.08	+ non sig
Shier, 2016	Small Food Stores	Unhealthy	Home	Number of small grocery	Count	Diet	Soda	All	? 0.007, SE 0.05	-non sig
Shier, 2016	Small Food Stores	Unhealthy	Home	Number of small grocery	Count	Diet	Sweet snacks	All	? -0.110, SE 0.1	+ non sig
Shier, 2016	Small Food Stores	Unhealthy	Home	Number of small grocery	Count	Diet	Salty snacks	All	? -0.027, SE 0.0	+ non sig
Shier, 2016	Small Food Stores	Unhealthy	Home	Number of small grocery	Count	Diet	Ready made di	All	? -0.019, SE 0.0	+ non sig
Shier, 2016	Supermarket	Healthy	Home	Number of supermarkets	Count	Diet	Fruit	All	? -0.319, SE 0.1	- non sig
Shier, 2016	Supermarket	Healthy	Home	Number of supermarkets	Count	Diet	Vegetables	All	? 0.037, SE 0.15	+ non sig
Shier, 2016	Supermarket	Healthy	Home	Number of supermarkets	Count	Diet	Soda	All	? - 0.113, SE 0.0	+ non sig
Shier, 2016	Supermarket	Healthy	Home	Number of supermarkets	Count	Diet	Sweet snacks	All	? - 0.103, SE 0.3	+ non sig
Shier, 2016	Supermarket	Healthy	Home	Number of supermarkets	Count	Diet	Salty snacks	All	? -0.458, SE 0.1	+ sig
Shier, 2016	Supermarket	Healthy	Home	Number of supermarkets	Count	Diet	Ready made di	All	? -0.036, SE 0.0	+ non sig
Smith, 2013	Grocery stores	Healthy	School	Median distance to	Distance	Diet	Healthy diet sc	All	? 0.003 (95%CI	- sig
Smith, 2013	Fast Food Outlets	Unhealthy	School	Median distance to	Distance	Diet	Healthy diet sc	All	? 0.002 (95%CI	+ sig
Smith, 2013	Fast Food Outlets	Unhealthy	School	Median distance to	Distance	Diet	Unhealthy diet	All	? -0.003 (95%C	- sig
Smith, 2013	Grocery stores	Healthy	School	Minimum distance to	Distance	Diet	Healthy diet sc	All	? 0.003 (95%CI	- sig

Smith, 2013	Grocery stores	Healthy	School	Minimum distance to	Distance	Diet	Healthy diet sc	All	? 0.002 (95%CI	-	sig
Smith, 2013	Grocery stores	Healthy	School	Minimum distance to	Distance	Diet	Unhealthy diet	All	? -0.001 (95%C	-	sig
Smith, 2013	Fast Food Outlets	Unhealthy	School	Minimum distance to	Distance	Diet	Healthy diet sc	All	? 0.002 (95%CI	+	sig
Smith, 2013	Fast Food Outlets	Unhealthy	School	Minimum distance to	Distance	Diet	Unhealthy diet	All	? -0.003 (95%C	+	sig
Smith, 2013	Fast Food Outlets	Unhealthy	School	Minimum distance to	Distance	Diet	Unhealthy diet	All	? -0.002 (95%C	+	sig
Sturm, 2011	Price	Consumer	netropolitan are	Price index for F&V	Consumer: Pric	Diet	F&V	All	? -0.82 (SE0.29,	+	sig
Sturm, 2011	Price	Consumer	netropolitan are	Price index for FF	Consumer: Pric	Diet	FF	All	? 0.21 (SE 0.08)	-	sig
Sturm, 2011	Price	Consumer	netropolitan are	Price index for SSB	Consumer: Pric	Diet	SSB	All	? 0.10 (SE 0.17)	-	non sig
Svastisalee, 2012	Supermarket	Healthy	School	Low density (1 or less) VS	Density	Diet	Infrequent fruit	All	OR 1.17 (95%CI 0.89,	+	non sig
Svastisalee, 2012	Supermarket	Healthy	School	Low density (1 or less) VS	Density	Diet	Infrequent fruit	All	OR 1.17 (95%CI 0.97,	+	non sig
Svastisalee, 2012	Supermarket	Healthy	School	Low density (1 or less) VS	Density	Diet	Infrequent fruit	All	OR 1.08 (95%CI 0.80,	+	non sig
Svastisalee, 2012	Supermarket	Healthy	School	Low density (1 or less) VS	Density	Diet	Infrequent veg consumption	All	OR 1.33 (95%CI 0.92,	+	non sig
Svastisalee, 2012	Supermarket	Healthy	School	Low density (1 or less) VS	Density	Diet	Infrequent veg consumption	All	OR 1.19 (95%CI 0.92,	+	non sig
Svastisalee, 2012	Supermarket	Healthy	School	Low density (1 or less) VS	Density	Diet	Infrequent veg consumption	All	OR 1.04 (95%CI 0.80,	+	non sig
Svastisalee, 2012	Fast Food Outlets	Unhealthy	School	High (3+) vs Low density (2	Density	Diet	Infrequent fruit	All	OR 1.32 (95%CI 0.98,	+	non sig
Svastisalee, 2012	Fast Food Outlets	Unhealthy	School	High (3+) vs Low density (2	Density	Diet	Infrequent fruit	All	OR 1.18 (95%CI 0.97,	+	non sig
Svastisalee, 2012	Fast Food Outlets	Unhealthy	School	High (3+) vs Low density (2	Density	Diet	Infrequent fruit	All	OR 1.23 (95%CI 0.89,	+	non sig
Svastisalee, 2012	Fast Food Outlets	Unhealthy	School	High (3+) vs Low density (2	Density	Diet	Infrequent veg consumption	All	OR 1.17 (95%CI 0.80,	+	non sig

Svastisalee, 2012	Fast Food Outlets	Unhealthy	School	High (3+) vs Low density (2	Density	Diet	Infrequent veg consumption	All	OR 1.20 (95%CI 0.92,	+ non sig
Svastisalee, 2012	Fast Food Outlets	Unhealthy	School	High (3+) vs Low density (2	Density	Diet	Infrequent veg consumption	All	OR 1.26 (95%CI 0.95,	+ non sig
Svastisalee, 2012	Unsupportive Environment	Unhealthy	School	Low density of supermarkets	Density	Diet	Infrequent fruit	All	OR 1.60 (95%CI 1.02,	+ sig
Svastisalee, 2012	Unsupportive Environment	Unhealthy	School	Low density of supermarkets	Density	Diet	Infrequent fruit	All	OR 1.40 (95%CI 1.03,	+ sig
Svastisalee, 2012	Unsupportive Environment	Unhealthy	School	Low density of supermarkets	Density	Diet	Infrequent fruit	All	OR 1.45 (95%CI 0.89,	+ non sig
Svastisalee, 2012	Unsupportive Environment	Unhealthy	School	Low density of supermarkets	Density	Diet	Infrequent veg consumption	All	OR 1.79 (95%CI 0.99,	+ non sig
Svastisalee, 2012	Unsupportive Environment	Unhealthy	School	Low density of supermarkets	Density	Diet	Infrequent veg consumption	All	OR 1.14 (95%CI 0.76,	+ non sig
Svastisalee, 2012	Unsupportive Environment	Unhealthy	School	Low density of supermarkets	Density	Diet	Infrequent veg consumption	All	OR 1.27 (95%CI 0.84,	+ non sig
Svastisalee, 2012	All Food outlets	Total/Unhealthy	School	Low density of supermarkets	Density	Diet	Infrequent fruit	All	OR 1.10 (95%CI 0.78,	- non sig
Svastisalee, 2012	All Food outlets	Total/Unhealthy	School	Low density of supermarkets	Density	Diet	Infrequent fruit	All	OR 1.11 (95%CI 0.88,	- non sig
Svastisalee, 2012	All Food outlets	Total/Unhealthy	School	Low density of supermarkets	Density	Diet	Infrequent fruit	All	OR 0.91 (95%CI 0.64,	+ non sig
Svastisalee, 2012	All Food outlets	Total/Unhealthy	School	Low density of supermarkets	Density	Diet	Infrequent veg consumption	All	OR 1.11 (95%CI 0.71,	- non sig
Svastisalee, 2012	All Food outlets	Total/Unhealthy	School	Low density of supermarkets	Density	Diet	Infrequent veg consumption	All	OR 1.05 (95%CI 0.71,	- non sig
Svastisalee, 2012	All Food outlets	Total/Unhealthy	School	Low density of supermarkets	Density	Diet	Infrequent veg consumption	All	OR 0.95 (95%CI 0.69,	+ non sig
Svastisalee, 2012	All Food outlets	Total/Unhealthy	School	High density of supermarkets	Density	Diet	Infrequent fruit	All	OR 1.17 (95%CI 0.75,	+ non sig
Svastisalee, 2012	All Food outlets	Total/Unhealthy	School	High density of supermarkets	Density	Diet	Infrequent fruit	All	OR 1.09 (95%CI 0.81,	+ non sig
Svastisalee, 2012	All Food outlets	Total/Unhealthy	School	High density of supermarkets	Density	Diet	Infrequent fruit	All	OR 0.94 (95%CI 0.61,	-non sig
Svastisalee, 2012	All Food outlets	Total/Unhealthy	School	High density of supermarkets	Density	Diet	Infrequent veg consumption	All	OR 0.87 (95%CI 0.50,	-non sig

Svastisalee, 2012	All Food outlets	Total/Unhealthy	School	High density of supermarkets	Density	Diet	Infrequent veg consumption	All	OR 0.91 (95%CI 0.58,	-non sig
Svastisalee, 2012	All Food outlets	Total/Unhealthy	School	High density of supermarkets	Density	Diet	Infrequent veg consumption	All	OR 0.94 (95%CI 0.62,	-non sig
Svastisalee, 2015	Fast Food Outlets	Unhealthy	School	1 fast food outlet within	Density	Diet	Weekly fast food	All	OR 0.99 (95% CI 0.73, 1.36)	-non sig
Svastisalee, 2015	Fast Food Outlets	Unhealthy	School	2+ fast food outlets within	Density	Diet	Weekly fast food	All	OR 1.05 (95%CI 0.77,	+ non sig
Timperio, 2018	FFO & covenience stores	Unhealthy	Home	Living in a neighbourhoo	Density	Diet	Healthful dietary	All	? -0.03 (95%CI - 0.22, 0.15)	+ non sig
Timperio, 2018	All food outlets	Total/Unhealthy	Home	Living in a neighbourhoo	Density	Diet	Healthful dietary	All	? 0.16 (95%CI-(	+ non sig
Timperio, 2018	FFO & covenience stores	Unhealthy	Home	Living in a neighbourhoo	Density	Diet	Healthful dietary	All	? -0.20 (95%CI -	+ non sig
Timperio, 2018	All food outlets	Total/Unhealthy	Home	Living in a neighbourhoo	Density	Diet	Healthful dietary	All	? -0.29 (95%CI -	-non sig
Timperio, 2018	FFO & covenience stores	Unhealthy	Home	Living in a neighbourhoo	Density	Diet	Energy dense pattern		? 0.11 (95%CI -	+ non sig
Timperio, 2018	All food outlets	Total/Unhealthy	Home	Living in a neighbourhoo	Density	Diet	Energy dense pattern		? 0.09 (95%CI -	+ non sig
Timperio, 2018	FFO & covenience stores	Unhealthy	Home	Living in a neighbourhoo	Density	Diet	Energy dense pattern	All	? 0.13 (95%CI -(	+ non sig
Timperio, 2018	All food outlets	Total/Unhealthy	Home	Living in a neighbourhoo	Density	Diet	Energy dense pattern	All	? 0.19 (95%CI -	+ non sig
Trapp, 2021	Fast Food Outlets	Unhealthy	School	Frequency of FFO in 400m	Count/density	Purchasing	Snack (discretionary	All	OR 1.03 (95%CI	+ non sig
Trapp, 2021	Fast Food Outlets	Unhealthy	School	Frequency of FFO (top 4	Count/density	Purchasing	Snack (discretionary	All	OR 1.26 (95%CI	+ sig
Trapp, 2021	Supermarkets	Healthy	School	Frequency of supermarkets	Count/density	Purchasing	Snack (discretionary	All	OR 1.07 (95%CI	- non sig
Trapp, 2021	Convenience Stores	Unhealthy	School	Frequency of convenience	Count/density	Purchasing	Snack (discretionary	All	OR 0.96 (95%CI	-non sig
Trapp, 2021	Other snack outlet	Unhealthy	School	Frequency of other snack	Count/density	Purchasing	Snack (discretionary	All	OR 0.99 (95% C	-non sig
Trapp, 2021	it and vegetable ou	Healthy	School	Frequency of fruit and veg	Count/density	Purchasing	Snack (discretionary	All	OR 1.13 (95%CI	- non sig

Trapp, 2021	Fast Food Outlets	Unhealthy	School	Frequency of FFO in 800m	Count/density	Purchasing	Snack (discretionary	All	OR 1.00 (95%CI	incon
Trapp, 2021	Fast Food Outlets	Unhealthy	School	Frequency of FFO (top 4	Count/density	Purchasing	Snack (discretionary	All	OR 1.14 (95%CI	+ non sig
Trapp, 2021	Supermarkets	Healthy	School	Frequency of supermarkets	Count/density	Purchasing	Snack (discretionary	All	OR 1.09 (95% C	- non sig
Trapp, 2021	Convenience Store:	Unhealthy	School	Frequency of convenience	Count/density	Purchasing	Snack (discretionary	All	OR 0.99 (95%CI	-non sig
Trapp, 2021	Other snack outlet	Unhealthy	School	Frequency of other snack	Count/density	Purchasing	Snack (discretionary	All	OR 1.00 (95%CI	incon
Trapp, 2021	it and vegetable ou	Healthy	School	Frequency of fruit and veg	Count/density	Purchasing	Snack (discretionary	All	OR 1.10 (95%CI	- non sig
Trapp, 2021	Fast Food Outlets	Unhealthy	School	Frrequency of FFO in 1km	Count/density	Purchasing	Snack (discretionary	All	OR 1.00 (95%CI	incon
Trapp, 2021	Fast Food Outlets	Unhealthy	School	Frequency of FFO (top 4	Count/density	Purchasing	Snack (discretionary	All	OR 1.17 (95%CI	+ non sig
Trapp, 2021	Supermarkets	Healthy	School	Frequency of supermarkets	Count/density	Purchasing	Snack (discretionary	All	OR 1.07 (95%CI	- non sig
Trapp, 2021	Convenience Store:	Unhealthy	School	Frequency of convenience	Count/density	Purchasing	Snack (discretionary	All	OR 0.95 (95%CI	-non sig
Trapp, 2021	Other snack outlet	Unhealthy	School	Frequency of other snack	Count/density	Purchasing	Snack (discretionary	All	OR 1.00 (95%CI	incon
Trapp, 2021	it and vegetable ou	Healthy	School	Frequency of fruit and veg	Count/density	Purchasing	Snack (discretionary	All	OR 1.03 (95%CI	- non sig
van der Horst, 2008	Supermarket	Healthy	School	Presence of supermarket	Count/density	Diet	SSB consumptic	All	? 0.077	- non sig
van der Horst, 2008	Fast Food Outlets	Unhealthy	School	High presence of FFO within	Count/density	Diet	SSB consumptic	All	? -0.055	-non sig
van der Horst, 2008	Small Food Stores	Unhealthy	School	Medium numbers of	Count/density	Diet	SSB consumptic	All	? -0.322, p<0.0	+ sig
van der Horst, 2008	Small Food Stores	Unhealthy	School	High numbers of small food	Count/density	Diet	SSB consumptic	All	? -0.259, p<0.0	+ sig
van der Horst, 2008	All Food outlets	Total/Unhealthy	School	A food store present in 200-	Distance	Diet	SSB consumptic	All	? -0.376, p<0.0	+ non sig
van der Horst, 2008	All Food outlets	Total/Unhealthy	School	A food store present in	Distance	Diet	SSB consumptic	All	? -0.098	+ non sig

Virtanen, 2015	All Food outlets	Total/Unhealthy	School	Closest FFO or grocery store	Distance	Purchase	Snacks	All	OR 1.53 (95%CI + non sig
Virtanen, 2015	All Food outlets	Total/Unhealthy	School	Closest FFO or grocery store	Distance	Purchase	Snacks	All	OR 2.01 (95%CI + non sig

Supplementary Table 10: Vote counting results for intervention studies

Author, year	Food Outlet Type	Intervention type	Intervention classification	Outcome	Food type	Sex	Result	Direction
Lawman, 2015	Convenience stores	Increase availability of healthy foods plus social marketing and training	Healthy	Purchases	Energy (kcals)	Both	A non-significant increase in energy content of purchases (kcals) was observed between intervention BL and FU: (Change 10.12; ; p>0.05)	- non sig
Shin, 2015	Convenience stores and takeaways	Promotion and increased availability of healthy foods in 3 conveneince stores and/or takeaways within 0.5 miles of recreational centre	Healthy	Purchases	Healthy	Both	A non-significant decrease in purchasing frequency of healthful foods in both intervention (-0.32, p=0.13) and control groups (-0.62, p=0.14) between BL and FU	- non sig
Shin, 2015	Convenience stores and takeaways	Promotion and increased availability of healthy foods in 3 conveneince stores and/or takeaways within 0.5 miles of recreational centre	Healthy	Purchases	Unhealthy	Both	A non-significant decrease in purchasing frequency of unhealthful foods in both intervention (-0.32, p=0.13) and control groups (-0.62, p=0.14) between BL and FU	+ non sig
